# Radio-sensitizing effects of VE-821 and beyond: Distinct phosphoproteomic and metabolomic changes after ATR inhibition in irradiated MOLT-4 cells

**DOI:** 10.1371/journal.pone.0199349

**Published:** 2018-07-12

**Authors:** Barbora Šalovská, Hana Janečková, Ivo Fabrik, Radana Karlíková, Lucie Čecháková, Martin Ondrej, Marek Link, David Friedecký, Aleš Tichý

**Affiliations:** 1 Department of Radiobiology, Faculty of Military Health Sciences in Hradec Králové, University of Defence in Brno, Hradec Králové, Czech Republic; 2 Department of Genome Integrity, Institute of Molecular Genetics of the Czech Academy of Sciences, Prague, Czech Republic; 3 Laboratory for Inherited Metabolic Disorders, Faculty of Medicine and Dentistry, Palacký University Olomouc, Olomouc, Czech Republic; 4 Department of Clinical Biochemistry, University Hospital Olomouc, Olomouc, Czech Republic; 5 Department of Molecular Pathology and Biology, Faculty of Military Health Sciences in Hradec Králové, University of Defence in Brno, Hradec Králové, Czech Republic; 6 Biomedical Research Center, University Hospital, Hradec Králové, Czech Republic; 7 Institute of Molecular and Translational Medicine, Faculty of Medicine and Dentistry, Palacký University Olomouc, Olomouc, Czech Republic; ENEA Centro Ricerche Casaccia, ITALY

## Abstract

Current anti-cancer strategy takes advantage of tumour specific abnormalities in DNA damage response to radio- or chemo-therapy. Inhibition of the ATR/Chk1 pathway has been shown to be synthetically lethal in cells with high levels of oncogene-induced replication stress and in p53- or ATM- deficient cells. In the presented study, we aimed to elucidate molecular mechanisms underlying radiosensitization of T-lymphocyte leukemic MOLT-4 cells by VE-821, a higly potent and specific inhibitor of ATR. We combined multiple approaches: cell biology techniques to reveal the inhibitor-induced phenotypes, and quantitative proteomics, phosphoproteomics, and metabolomics to comprehensively describe drug-induced changes in irradiated cells. VE-821 radiosensitized MOLT-4 cells, and furthermore 10 μM VE-821 significantly affected proliferation of sham-irradiated MOLT-4 cells. We detected 623 differentially regulated phosphorylation sites. We revealed changes not only in DDR-related pathways and kinases, but also in pathways and kinases involved in maintaining cellular metabolism. Notably, we found downregulation of mTOR, the main regulator of cellular metabolism, which was most likely caused by an off-target effect of the inhibitor, and we propose that mTOR inhibition could be one of the factors contributing to the phenotype observed after treating MOLT-4 cells with 10 μM VE-821. In the metabolomic analysis, 206 intermediary metabolites were detected. The data indicated that VE-821 potentiated metabolic disruption induced by irradiation and affected the response to irradiation-induced oxidative stress. Upon irradiation, recovery of damaged deoxynucleotides might be affected by VE-821, hampering DNA repair by their deficiency. Taken together, this is the first study describing a complex scenario of cellular events that might be ATR-dependent or triggered by ATR inhibition in irradiated MOLT-4 cells. Data are available via ProteomeXchange with identifier PXD008925.

## Introduction

DNA damage induction by either radio- or chemo-therapy has been the most widely used approach in oncology. Since most of the cancer cells possess defects in one or more DNA damage response (DDR) pathways and suffer from elevated levels of replication stress [1], an effective approach is to target tumour-specific abnormalities in DDR based on the synthetic lethality principle. An appropriate example of such a strategy is targeting the S and G2/M DNA damage checkpoints in G1/S DNA damage checkpoint deficient cells [2]. In a recent study investigating mutational profiles in 3,281 tumours across 12 tumour types [3], genes from the ATM/Chk2/p53 pathway were affected by mutations in almost a half of the investigated cancer cells. As this pathway is essential for maintaining the G1/S DNA damage checkpoint after irradiation, the results of this study suggested that targeting the remaining DNA damage checkpoints might be a promising strategy in a considerable proportion of solid tumours conventionally treated using radiotherapy. Another promising strategy is targeting proteins and protein kinases involved in replication stress response. Cancer cells deficient in G1/S checkpoint or with mutations deregulating replication origin firing suffer from premature entry into S-phase, and thus DNA replication can start before the necessary resources have been generated [4,5].

Inhibition of the ATR/Chk1 pathway has been shown to be synthetically lethal in both above-mentioned scenarios. It has been shown selectively toxic in cells with high levels of oncogene-induced replication stress [4,6–11], and ATR inhibition might be also efficient in combination with genotoxic therapy in p53- or ATM-deficient cells [12–16]. Importantly, two highly potent and selective inhibitors are currently being evaluated in clinical trials: VE-822 (or VX-970; [12]) and AZD6738 [16]. Taken together, selective targeting of the ATR/Chk1 pathway offers a promising therapeutic approach for cancer treatment in a broad range of tumours in both monotherapy and for the purpose of selectively sensitizing cancer cells to current genotoxic treatment.

The effects of ionizing radiation (IR) and other DNA damage inducing agents in MOLT-4 (p53-wildtype, T-cell acute lymphoblastic leukemia; T-ALL) cells have been previously studied [17–28]. Our team addressed the response of these cells to ionizing radiation extensively and we described IR-induced cell death [18,29], cell signalling [17,21,24], and suggested possible defect in DNA repair pathways promoting their radiosensitivity [22]. The presented study was driven by the fact that MOLT-4 cells suffer from multiple heterozygous mutations in several DDR genes (such as *ATM*, *BRCA1*, *FANCA*, and *TP53*) [30,31], implying the cell line might be sensitive to ATR inhibitor treatment.

In this study, we aimed to elucidate molecular mechanisms underlying radiosensitization of the MOLT-4 cell line by specific inhibition of ATR using a highly potent and selective inhibitor–VE-821. To do so, we adopted a unique strategy combining multiple approaches: i) cell biology techniques to investigate the inhibitor-induced phenotypes, ii) shotgun proteomics and phosphoproteomics to study protein phosphorylation triggered by irradiation and its modulation by kinase inhibitors, and iii) targeted metabolomics to reveal drug-induced changes in the metabolome of irradiated cells.

## Materials and methods

### Cell culture and cell culture conditions

MOLT-4 cells (European Collection of Authenticated Cell Cultures, Porton Down, Salisbury, UK) were cultured in IMDM (Thermo Fisher Scientific, MA, USA) containing 20% foetal bovine serum (Thermo Fisher Scientific, Waltham, MA, USA), 2 mM glutamine, 100 UI/mL penicillin, and 0.1 mg/mL streptomycin (all Sigma-Aldrich, St. Louis, MO, USA) at 37°C under a controlled 5% CO_2_ and humidified atmosphere. The cultures were split every other day by dilution to a concentration of 2x10^5^ cells/mL. Cell counts were assessed by a haemocytometer, and cell membrane integrity was assessed by the Trypan Blue exclusion technique.

For the quantitative proteomic and phosphoproteomic experiments, MOLT-4 cells well cultured in IMDM for SILAC (Thermo Fisher Scientific, Waltham, MA, USA), containing 20% dialyzed foetal bovine serum (Sigma-Aldrich, St. Louis, MO, USA). Media were further supplemented with either unlabelled l-lysine (100 mg/L, K0) and l-arginine (84 mg/L, R0) or equimolar amounts of l-^13^C_6_-lysine (K6) and l-^13^C_6_-arginine (R6; all Sigma-Aldrich, St. Louis, MO, USA). l-proline (300 mg/L; Sigma-Aldrich, St. Louis, MO, USA) was added to cell culture media to avoid metabolic conversion of arginine to proline [32]. For complete incorporation of labelled amino acids, cells were cultured for more than six cell doublings, usually 10 [33].

### Cell treatment (kinase inhibition and gamma-irradiation)

A selective inhibitor of ATR kinase, 3-amino-6-(4-(methylsulfonyl)phenyl)-*N*-phenylpyrazine-2-carboxamide (VE-821; APIs Chemical Co., Ltd., Shanghai, China) was dissolved in dimethyl sulfoxide (DMSO; Sigma-Aldrich, St. Louis, MO, USA) to a concentration of 10 mM, and the aliquots were stored at −20°C. Additional dilutions (1 mM, 2 mM, 5 mM, and inhibitor combinations as indicated) were also prepared in DMSO to maintain the same concentration of DMSO in all inhibitor-treated samples. In all experiments presented in this study, the inhibitors were added to the cell culture 30 minutes prior to gamma irradiation. The inhibitors were washed out after one hour or 24 hours when indicated. Cells were irradiated using a ^60^Co gamma-ray source (VF, Cerna Hora, Czech Republic) with a dose rate of 0.5–0.45 Gy/min. Since MOLT-4 cells are relatively sensitive to gamma irradiation, the doses of irradiation were carefully chosen based on our previous publications [34–36] to only induce either cytostatic or sub-lethal damage.

### Electrophoresis and western blotting

Cells were pre-treated with different concentrations of inhibitors or DMSO (control) and irradiated by an indicated dose of IR. One or six hours after irradiation, cells were washed with cold phosphate buffer saline (PBS; Thermo Fisher Scientific, MA, USA), and whole cell extracts were prepared by lysis in 500 μL of lysis buffer per 1x10^7^ cells (137 mM NaCl; 10% glycerol; 50 mM NaF; 20 mM Tris-HCl, pH = 8; 1% n-octyl-β-glucopyranoside; 1:100 Phosphatase inhibitor cocktail 2 and 3 –all from Sigma-Aldrich, St. Louis, MO, USA; 1 tablet of protease inhibitors Complete™ Mini/ 10 mL–Roche, Mannheim, Germany). The lysate was clarified by centrifugation, and the protein concentration measured by bicinchoninic acid protein assay. The lysates containing equal amounts of protein (30 μg) were loaded on to a 12% SDS polyacrylamide gel. After electrophoresis, proteins were transferred to a polyvinylidene difluoride membrane and hybridized with an appropriate antibody: anti-Chk1 (1:500; no. 2360, Cell signalling Technology®), anti-phospho-Chk1 (Ser345, 1:500; no. 2348, Cell signalling Technology®), anti-Chk2 (1:250; no. 2662, Cell signalling Technology®), anti-phospho-Chk2 (Thr68, 1: 250; no. 2197, Cell signalling Technology®), anti-p70s6k (1:1000; no. 2708, Cell signalling Technology®), anti-phospho p70s6k (Thr389; 1:1000; no. 9234, Cell signalling Technology®) and beta actin (1: 20,000; no. A5316, Sigma-Aldrich, St. Louis, MO, USA). After washing, the membranes were incubated with secondary peroxidase-conjugated antibody (no. P0447 and P039901, Dako, Glostrup, Denmark), and the signal was developed using a BM Chemiluminescence Western Blotting Kit (Roche, Mannheim, Germany).

### Cell proliferation/viability WST-1 assay

Proliferation of MOLT-4 cells was evaluated by WST-1 (4-[3-(4-iodophenyl)-2-(4-nitrophenyl)-2H-5-tetrazolio]-1,3-benzene disulphonate) colorimetric assay (Roche Diagnostics, Mannheim, Germany). The assay is based on cleavage of WST-1 by mitochondrial dehydrogenases, and the absorbance of the cleavage product correlates with the number of viable cells. Prior to treatment, MOLT-4 cells were placed in 96-well microplates; the number of cells plated into each well were 3x10^4^, 1x10^4^, and 2.5x10^3^ for 24, 72, and 144 hour long experiments, respectively. After the addition of inhibitors and subsequent irradiation, the microplates were placed in an incubator and cultivated at 37°C. Finally, WST-1 was added, and the plates were incubated for three hours at 37°C. Absorbance of samples was then measured at 440 nm using a PARADIGM™ Detection Platform (Beckman Coulter, Brea, CA, USA). Controls were normalized to 100% for each assay, and all values were expressed as a percentage of the normalized controls.

### Cell cycle analysis and apoptosis detection by flow cytometry

For cell cycle analysis, cells were collected 24 hours after irradiation by a dose of 3 Gy (± inhibitor pre-treatment), washed with ice-cold PBS, and fixed with ice-cold 70% ethanol. Prior to flow cytometry, the cells were washed with ice-cold PBS, and DNA was stained using Vindelov’s solution (10 mM Tris-HCl pH = 7.6, 0.6 mg/mL NaCl, 0.01 mg/mL Ribonuclease A, 0.05 mg/mL PI; all Sigma-Aldrich, St. Louis, MO, USA).

For apoptosis detection, an Apoptest-FITC kit was used (DakoCytomation, Copenhagen, Denmark). The kit contains Annexin V, which binds to phosphatidylserine at the surface of apoptotic cells, and propidium iodide (PI) to detect cells with increased cell membrane permeability. Cells were pre-treated with the inhibitors and irradiated by a dose of 1 Gy. The proportion of apoptotic cells in each condition was measured 24 hours and 72 hours after IR according to the manufacturer’s instructions.

Flow cytometric analysis was performed on a FACS analyser CyAn DakoCytomation (Beckman Coulter, Miami, FL, USA). At least 20,000 cells were analysed per sample. Listmode data were analysed using Summit v4.3 software (Beckman Coulter, Miami, FL, USA).

### Sample preparation for quantitative proteomic and phosphoproteomic experiment

MOLT-4 cells well cultured in IMDM for SILAC containing 20% of dialyzed foetal calf serum for at least six cell doublings as described before [33,37]. Thirty minutes before irradiation, VE-821 was added to the “heavy” cells (K6/R6) at a concentration of 10 μM; the “light” cells (K0/R0) were mock treated with DMSO, whose final concentration in culture was lower than 0.1%. Both groups were irradiated using a ^60^Co gamma-ray source by a dose of 1.5 Gy. After irradiation, the flasks were placed into an incubator. Three biological replicates were analysed.

One hour after irradiation, the cells were washed with ice-cold PBS and lysed as was published [38] with minor modifications. Briefly, the cells were thoroughly resuspended in 2 mL ice-cold lysis buffer/1x10^7^ cells (50 mM ammonium bicarbonate, 1% sodium deoxycholate, 1:100 Phosphatase inhibitor cocktail 2 and 3, all Sigma-Aldrich, St. Louis, MO, USA). The lysate was immediately placed into a boiling water bath, and after 5 min incubation, the samples were cooled down to room temperature on ice. To cleave nucleic acids and decrease the viscosity of the lysate, bensonase nuclease (2.5 U/μL) and MgCl_2_ (1.5 mM) were added to the samples (both Sigma-Aldrich, St. Louis, MO, USA). The lysate was then clarified by centrifugation at 14,000 rpm, and protein concentration was measured by bicinchoninic acid assay. Sample volumes corresponding to 2 mg of “light” proteins and 2 mg of “heavy” proteins were pooled together to make a 1:1 protein sample.

The protein samples for phosphoproteomic analyses were diluted in lysis buffer and reduced with 10 mM DTT, alkylated with 20 mM IAA (both Sigma-Aldrich, St. Louis, MO, USA), and digested with trypsin (Sequencing grade modified, Promega Corporation, Madison, WI, USA) overnight at an enzyme-to-substrate ratio of 1:60 (sequence grade modified trypsin). Sodium deoxycholate was then extracted by ethyl acetate as described earlier [39]; tryptic peptides were desalted via 500 mg Supelco C18 SPE cartridges according to the manufacturer’s instructions and dried using SpeedVac.

Sample preparation for the proteome analysis followed the protocol described above with minor modifications to adjust the volume and concentration of chemicals to a smaller sample amount (10+10 μg of the “heavy” and “light” samples).

### Hydrophilic interaction liquid chromatography fractionation

Dried peptide samples were fractionated by HILIC according to a protocol that has been published previously [40] using a 4.6 × 25 cm TSKgel® Amide-80 HR 5 μm particle column with a TSKgel® Amide-80 HR 5 μm 4.6 × 1 cm guard column (Tosoh Biosciences, Stuttgart, Germany) operated with Waters Separations Module e2695 (Waters, Milford, MA, USA) at 0.5 mL/min. Briefly, 3.5 mg of evaporated samples were reconstituted in 80% B and loaded on to the HILIC column. Peptides were then separated by a gradient of A over B from 80% to 60% B in 40 min and from 60% to 0% B in 5 min. Across the gradient, 22 fractions were collected (2 × 2 and 20 × 1 mL) from each replicate. Mobile phase B consisted of 98% acetonitrile (Acn)/0.1% TFA; mobile phase A consisted of 2% Acn/0.1% TFA (all solvents purchased from Sigma-Aldrich, St. Louis, MO, USA).

### Phosphopeptide enrichment

Each HILIC fraction was enriched for phosphopeptides using titanium dioxide chromatography [41] using a protocol optimized in previous experiments [42–44]. At first, each fraction was supplemented with TFA and glutamic acid to reach final concentrations of 2% TFA and 100 mM glutamic acid. Titanium dioxide particles (Titansphere® 5 μm particles, GL Sciences, Japan) were suspended in a loading solution (65% Acn, 2% TFA, 100 mM glutamic acid; all Sigma-Aldrich, St. Louis, MO, USA), and a volume of titanium dioxide suspension depending on the expected proportion of peptides and phosphopeptides in a particular fraction (based on previous experiments) was added to each sample. Microparticles with bound phosphopeptides were washed with 200 μL of loading solution, 200 μL of washing solution 1 (65% Acn/0.5% TFA), 200 μL of washing solution 2 (65% Acn/0.1% TFA) and 100 μL of washing solution 2. Phosphopetides were eluted by 150 μL of elution solution (20% Acn/NH_4_OH, pH 11.5) in two sequential elutions. Late fractions were subjected to a second enrichment. Eluates from the first and second enrichment were pooled together, acidified with 100% formic acid, and placed in a SpeedVac until all crystals of ammonium formate were evaporated.

### Mass spectrometric analysis

Liquid chromatography coupled with tandem mass spectrometry (LC-MS/MS) analyses were performed on a Thermo Scientific Dionex Ultimate™ 3000 RSLCnano system (Thermo Scientific, Bremen, Germany) coupled through Nanospray Flex ion source with Q Exactive mass spectrometer (Thermo Scientific, Bremen, Germany). TiO_2_-enriched HILIC fractions were dissolved in 18 μL of 2% Acn/0.05% TFA and 3–8 μL according to the estimated peptide sample concentration were injected into the RSLCnano system. Peptides were loaded on a capillary trap column (C18 PepMap100, 3 μm, 100 Å, 0.075 × 20 mm) by 2% Acn/0.05% TFA mobile phase at flow rate 5 μL/min for 5 min and then eluted and separated on capillary column C18 PepMap RSLC, 2 μm, 100 Å, 0.075 × 150 mm (Dionex, Thermo Scientific™, Bremen, Germany). Elution was performed by step linear gradient of mobile phase B (80% Acn/0.1% FA) over mobile phase A (0.1% FA) from 4% to 34% B in 48 min and from 34% to 55% B in 15 min at a flow rate of 300 nl/min. The temperature of the column was 40°C and eluent was monitored at 215 nm during the separation. Spraying voltage was 1.7 kV and heated capillary temperature was 250°C. The mass spectrometer was operated in the positive ion mode performing a survey MS (range 300 to 1800 *m*/*z*) and data-dependent MS/MS scans performed on the six most intense precursors with dynamic exclusion window of 40 s. MS scans were acquired with 70,000 resolution at 200 *m/z* from 1 × 10^6^ accumulated charges (maximum fill time was 100 ms). Intensity threshold for triggering MS/MS was set at 5 × 10^4^ for ions with z ≥ 2 with a 1.6 Da isolation window. Precursor ions were accumulated with AGC of 1 × 10 (maximum fill time was 100 ms) and the normalized collisional energy for HCD fragmentation was 27 units. MS/MS spectra were acquired with 17,500 resolution (at 200 *m/z*). The mass spectrometry proteomics data have been deposited to the ProteomeXchange Consortium via the PRIDE [45] partner repository with the dataset identifier PXD008925.

### Mass spectrometry data processing and bioinformatic analysis

#### Raw data processing

Raw data files acquired by LC-MS/MS were processed with MaxQuant *v1*.*5*.*2*.*8* [46]. Peak lists were searched against the human SwissProt database (November 2015) using Andromeda search engine [47]. Minimum peptide length was set to seven amino acids, and two missed cleavages were allowed. Carbamidomethylation of cysteine was set as a fixed modification while oxidation of methionine, protein N-terminal acetylation, and phosphorylation of serine, threonine, and tyrosine residues were used as variable modifications. Additionally, appropriate SILAC labels were selected (R6, K6), and the labelled amino acid filtering was disabled. Mass tolerances of 10 and 20 ppm were allowed for MS and MS^2^ peaks, respectively. Only proteins, peptides, and phosphorylation sites with false discovery rate (FDR) lower than 0.01 were accepted. For modified peptides, a minimal score (40) and minimal delta score (6) were set as additional cut-offs. For protein quantification, only unmodified peptides, peptides oxidized at methionine residues, or acetylated at the *N*-terminus were accepted; both razor and unique peptides were used for calculation of protein H/L ratios. The Re-quantify function was disabled, whereas Match between runs was enabled during the search.

#### MaxQuant output data filtering and identification of significantly changed phosphorylation sites

Potential contaminants and hits from the reversed database were removed before further data processing, and data were further manually inspected to look for possible misquantifications caused by the labelling status (K6/R6) and disabled “Filter labelled amino acids” option in MaxQuant. Global rank test (GRT) was used to find differentially regulated phosphorylation sites. Only phosphorylation sites quantified in all three replicates were subjected to GRT, and FDR was estimated non-parametrically as described by Zhou et al. [48]. The significance cut-off for differentially regulated sites was set to FDR < 0.005.

#### Gene ontology and signalling pathways over-representation analyses

Gene ontology (GO) and signalling pathways over-representation analyses were performed using the ConsensusPathDB over-representation analysis web tool [49,50]. For the GO terms over-representation, proteins containing differentially regulated phosphorylation sites (both up- and down-) were tested against a custom background reference set comprising all phosphoproteins with at least one quantified phosphorylation site that was subjected to GRT (*i*.*e*. all phosphoproteins with at least one phosphorylation site quantified in all three biological replicates). Statistical significance of the over-representation analysis was estimated using hypergeometric testing with Benjamini-Hochberg FDR correction of calculated p-values, and the cut-off was set to FDR < 0.05. For the signalling pathways over-representation analysis, phosphoproteins with at least one regulated phosphorylation site were mapped to signalling pathways from three different databases: KEGG [51,52], REACTOME [53], and PID [54]. Pathway-coverage was calculated for overrepresented pathways (FDR < 0.05, tested against a default background reference set comprising all Uniprot proteins included in at least one signalling pathway from a corresponding database).

#### Network analysis

Log_2_ transformed SILAC H/L ratios of phosphorylation sites quantified in all three biological replicates were normalized (z-score calculation) and subjected to SubExtractor algorithm [55]. Human protein-protein interactions with STRING score above 900 were used as the second input for the algorithm, and regulated subnetworks were extracted (FDR = 0.005, α = 0.2, σ = 4). Extracted networks were visualized using Cytoscape *v3*.*2*.*1* [56].

#### Sequence motif analyses

To analyse and visualize sequence motifs surrounding phosphorylation sites identified and quantified in our study, we employed the iceLogo tool [57] and motif-x algorithm [58]. In both motif analyses, amino acid sequences (± 7 residues) surrounding either significantly up- or down-regulated phosphorylation sites were tested against a background reference set composed of sequences surrounding non-regulated phosphorylation sites detected in our study (as evaluated using GRT) that reached a minimal localization probability of 0.75 (*i*.*e*., “class I phosphosites” [59]). Motif-x was employed to extract significantly enriched linear motifs. Search parameters were set to at least 10 occurrences of a motif, and the significance level to *p* < 0.00003 (which approximately corresponds to a *q*-value of 0.01 after Bonferroni correction for multiple hypothesis testing).

#### Identification of protein kinases responsible for the observed protein phosphorylations and evaluation of global changes in their activities

Class I phosphorylation sites quantified in all three biological replicates were annotated with previously known kinase-substrate relationships downloaded from the PhosphoSitePlus database [60]. To increase the number of annotated phosphorylation sites, two kinase predictors were employed: NetworKIN 3 [61] and iGPS *v1*.*0* [62]. Both predictors combine motif scoring with contextual information (*i*.*e*. protein-protein interaction scoring downloaded from the STRING database [63]). The kinase predictions were further filtered to reach NetworKIN score > 3 or “medium” significance threshold in iGPS (*i*.*e*. FDR of 6% for S/T kinases and FDR of 9% for Y kinases). The significance of global changes in kinase activities was evaluated using the “1D annotation enrichment” tool available in Perseus software *v1*.*5*.*2*.*6* [64].

To further facilitate the interpretation of the results we also downloaded the “Regulatory sites” dataset from the PhosphoSitePlus database, which summarizes the current up-to-date knowledge about the known functions of specific phosphorylation sites. In the text, these sites are referred as “regulatory” sites.

### Targeted metabolomic analysis

For the targeted metabolomic analysis, MOLT-4 cells were pre-treated either with VE-821 (10 μM) or DMSO (control groups) 30 minutes prior to IR (1.5 Gy). Six and twelve hours after irradiation, the cells were counted using a haemocytometer, and a cell suspension volume containing 2x10^6^ cells was pipetted into five sample volumes of a pre-cooled (-40°C) quenching solution (60% methanol, 0.85% ABC, pH 7.4) as described previously [65]. The samples were gently mixed, and the cells were pelleted by centrifugation (1,000 g for 3 min). The supernatant was removed and the cells were in one step resuspended, lysed and deproteinated in 150 μL of pre-cooled (-80°C) methanol, and freeze dried. Three replicates were prepared for each condition. Prior the LC-MS/MS analysis the lyophilisates were dissolved in 100 μl of 80% methanol and centrifuged (14,000 g for 10 min).

A quality control sample (QC) was prepared by pooling equal volumes (10 μl) of cell extract samples. It was analysed randomly throughout the run to provide a measurement not only of the stability of the system and performance, but also of the reproducibility of the sample preparation.

The metabolite profiling of cell extracts and the QC samples were performed by the high-performance LC-MS/MS method modified from Asara [66]. The method enabled the analysis of 354 intermediary metabolites (acylcarnitines, amino acids, organic acids, saccharides, etc.; [67]). Data processing, treatment, and statistical analysis of data were described in detail previously [67].

## Results and discussion

### VE-821 treatment abrogated the radiation-induced phosphorylation of checkpoint kinase-1 and did not affect ATM-mediated signalling

**Fig 1:**
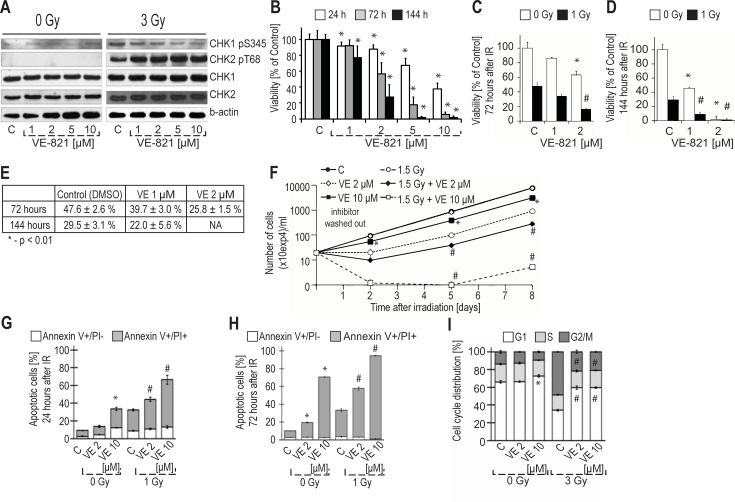
Radiosensitization of MOLT-4 cells by ATR kinase inhibitor VE-821. (A) Effect of VE-821 on activation of ATM and ATR kinases by gamma-irradiation. Cells were collected 1 hour after IR (3 Gy). Activation of both kinases and its suppression in the presence of VE-821 was monitored via detection of their specific phosphorylations targets. (B) Characterization of VE-821 treatment effects on proliferation of MOLT-4 cells. Viability was examined by WST-1 assay after 24, 72, and 144 hours of continuous inhibitor treatment. Data are expressed as percentage of viability (metabolic activity) of controls for each time interval. Mean values ± SD from at least five measurements are presented. *p ≤ 0.01 vs. corresponding controls. (C, D, E, F) Radio-sensitization of MOLT-4 cells using VE-821. Cells were irradiated by the indicated dose. Viability (metabolic activity) was examined by WST-1 assay after 72 hours (C) and 144 hours (D) of continuous inhibitor treatment. (E) Table shows an irradiated group/sham-irradiated group ratio for each treatment condition depicted in (C) and (D). (F) VE-821 was washed out 24 hours after irradiation and the number of viable cells was assessed by a haemocytometer 2, 5, and 8 days after irradiation with subsequent dilution to a concentration of 2x10^5^ cells/ml. Mean values ± SD from five measurements are presented. (G, H) Apoptosis induction in VE-821 treated irradiated MOLT-4 cells. Cell death was detected by Annexin V/PI staining 24 (G) and 72 hours (H) after IR (1 Gy). Mean values ± SD from three measurements are presented. (I) Cell cycle effects of ATR inhibition in irradiated MOLT-4 cells: Cell cycle perturbation was examined using propidium iodide staining of DNA detected by flow cytometry 24 hours after IR (3 Gy). Data are expressed as relative proportion of viable cells in different phases of the cell cycle. Mean values ± SD from three measurements are presented. In (A-I), * indicates statistical significance (p < 0.01) of comparison to non-irradiated control; **#** indicates statistical significance (p < 0.01) of comparison to irradiated control group. P-values were calculated using two-sample t-test. In all experiments, the cells were pre-treated with inhibitors at indicated concentrations or DMSO in control groups (C) 30 minutes prior to IR.

To confirm the inhibitory effect of VE-821 on ATR in our experimental model, we assessed phosphorylation of its effector kinase: checkpoint kinase-1 (Chk1) Ser 345 by immunoblotting (Fig 1A). As expected, the monitored phosphorylation site was upregulated upon irradiation (3 Gy, one hour after irradiation), and it was proportionally inhibited by increasing concentration of VE-821. Additionally, we observed that VE-821 did not inhibit radiation-induced phosphorylation of checkpoint kinase-2 (Chk2) Thr 68, a marker of IR-triggered ATM activity. Therefore, we confirmed that in our cell line model pre-incubation with VE-821 inhibited ATR kinase activity upon irradiation while ATM activity was not affected.

### VE-821 is a potent inhibitor of MOLT-4 cell growth both in single treatment and in combination with irradiation

To asses the effects of VE-821 on MOLT-4 cells viability, we first analysed whether the inhibitor affects proliferation of MOLT-4 cells after single treatment. We observed that VE-821 significantly inhibited proliferation of MOLT-4 cells at 1 μM concentration (Fig 1B). This effect was further accentuated in correlation with increasing inhibitor concentration. Thus, in MOLT-4 cells, inhibition of ATR by VE-821 has a strong effect on proliferation.

In combination with IR, VE-821 significantly abated the number of viable cells in both irradiated groups just 72 hours after irradiation (Fig 1C and 1D). As both concentrations used in our experiments directly affected the proliferation of sham-irradiated cells, we further calculated a ratio between the irradiated and sham-irradiated group for each inhibitor-treated condition to resolve if the combination of irradiation and the inhibitor has a more profound effect on proliferation than the inhibitor itself (Fig 1E). Whereas the irradiation caused nearly 50% decrease of control cell viability after 72 hours of treatment, the irradiation of pre-treated cells (2 μM VE-821) led to almost 75% loss of viability in comparison to inhibitor-treated cells, suggesting an additive effect of the VE-821 and IR combination. Additionally, we also observed that VE-821 in 2 μM and 10 μM concentrations modulated the proliferation of irradiated MOLT-4 cells when the inhibitor was present in cell culture media only transiently, for the first 24 hours of the treatment, and was then washed out (Fig 1F). In contrast to continuous treatment, 2 μM VE-821 did not significantly affect the proliferation of sham-irradiated cells when washed out after 24 hours; however, it still enhanced the antiproliferative effects of IR. Taken together, these data showed that VE-821 strongly affected the proliferation of p53-wt MOLT-4 cells, and in combination with IR, the proliferation was influenced even when the inhibitor was present only transiently.

### VE-821 increased the ionizing radiation-induced cell death in MOLT-4 cells and disrupted ionizing radiation-induced G2/M arrest in MOLT-4 cells

We further assessed the impact of VE-821 on the viability of MOLT-4 cells after IR. As shown in Fig 1G 10 μM VE-821 significantly affected the viability of MOLT-4 cells 24 hours after the addition of the treatment and/or irradiation. The effect induced by 10 μM VE-821 was comparable to 1 Gy of IR; almost 40% of PI-positive cells were detected under both treatment conditions. When combined with IR, VE-821 in both concentrations significantly increased the IR-induced cell death. Three days after irradiation, in the VE-821 treated groups the percentage of cells with compromised viability further decreased despite the absence of the inhibitor in the cultivation medium (Fig 1H). Importantly, VE-821 increased the sensitivity of MOLT-4 cells to IR. The strongest effect was observed when cells were treated with 10 μM VE-821. Application of these conditions led to more than 90% decrease in viability of MOLT-4 cells 72 hours after irradiation. In conclusion, application of the inhibitor led to increased cell death when combined with IR and thus radiosensitized MOLT-4 cells.

As one of the proposed mechanisms of radiosensitization using ATR inhibitors is the disruption of the G2/M checkpoint in G1 checkpoint-deficient cells [15], we investigated modulation of the cell cycle by ATR inhibition (Fig 1I). None of the conditions we applied affected the cell cycle in sham-irradiated cells except for 10 μM VE-821, which repeatedly caused a significant increase in the number of cells in G1 (p < 0.01). In concordance with our previous results [68], irradiation by a dose of 3 Gy led to significant G2/M arrest in viable MOLT-4 cells 24 hours after irradiation. Pre-incubation with VE-821 led to a significant disruption of the G2/M checkpoint in the viable fraction of cells. The abrogation of the G2/M checkpoint by VE-821 10 μM was also achieved when a lower dose of irradiation was applied (1.5 Gy; data not shown). In summary, these results confirmed that ATR is the principal kinase controlling the G2/M DNA damage checkpoint after irradiation in MOLT-4 cells.

### Phosphoproteomic analysis of VE-821-modulated cellular response to ionizing radiation identified and quantified thousands of phosphorylation sites

**Fig 2:**
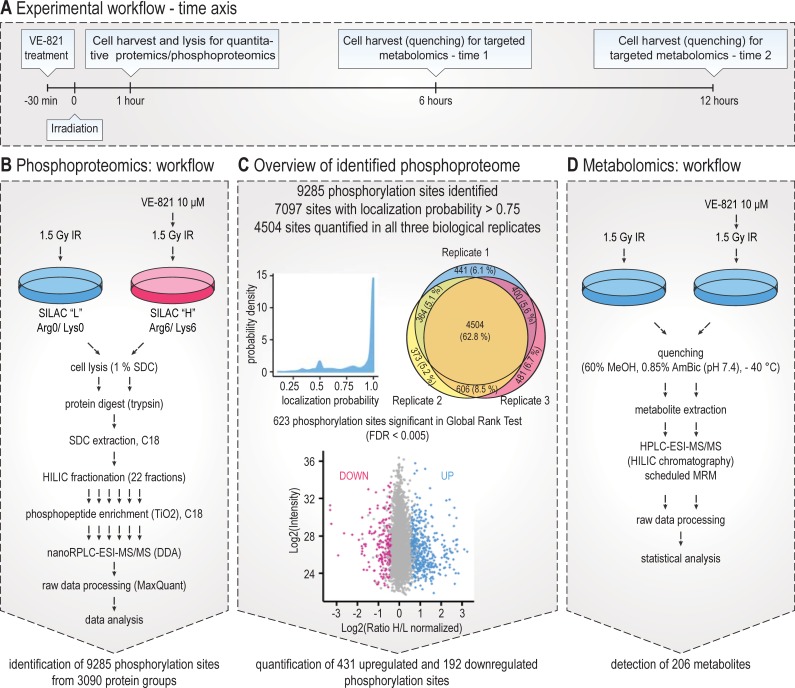
Identification and quantification of the VE-821-regulated phosphoproteome and metabolome in irradiated MOLT-4 cells using tandem mass spectrometry. (A) Overview of time intervals used for both phosphoproteomic and metabolomic analyses of VE-821 perturbed cellular response to ionizing radiation (IR). (B) Overview of experimental design and workflow used for quantitative SILAC-based phosphoproteomics. (C) Summary of the identified and quantified phosphoproteome. (D) Overview of experimental design and workflow used for targeted metabolomics screening.

**Fig 3:**
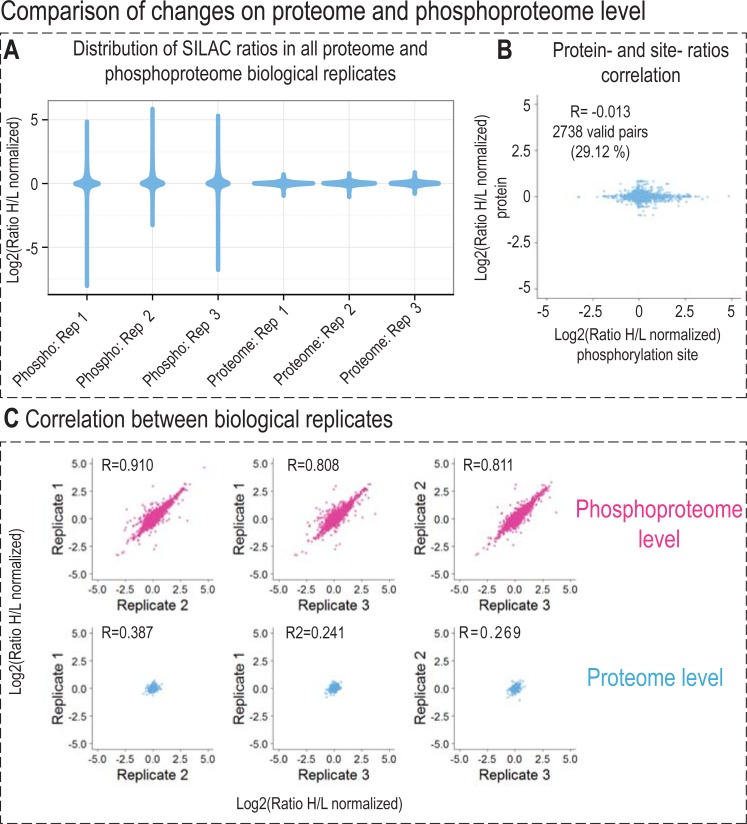
Comparison and correlation of changes on proteome and phosphoproteome level and their quantitative reproducibility. (A) Violin plots depict log_2_ transformed normalized SILAC H/L ratios distribution in all proteome and phosphoproteome biological replicates. (B) Pearson correlation between phosphosite and protein ratios. H/L ratios of 2738 phosphorylation sites (29% percent of the identified phosphorylation sites) were plotted against H/L ratios of corresponding proteins and the Pearson correlation value (R) was calculated. (C) Pearson correlation (R) between biological replicates of both proteome and phosphoproteome experiment.

To describe cellular mechanisms underlying the VE-821-mediated radiosensitization of MOLT-4 cells, we employed high-resolution MS to identify and quantify changes in the proteome and phosphoproteome of irradiated VE-821-treated cells (Fig 2B). In total, we identified 9,285 phosphorylation sites from 3,090 protein groups at a site FDR level of 0.01, amongst which 4,504 were quantified in all three biological replicates (nearly 63% of the data set, Fig 2C). The phosphosite ratios correlated very strongly between the replicates (Pearson correlation coefficient was between 0.8 and 0.9, Fig 3C). Only those sites quantified in all replicates were subjected to a non-parametric version of a global rank test ([48]) to identify sites with significantly up- or down-regulated phosphorylation consistently regulated in all three biological replicates. The sites subjected to GRT, their quantification, and basic annotation are given in S1 Table. In GRT, we identified 623 regulated phosphorylation sites (from 455 phosphoproteins), the majority of which were upregulated (431). As discussed previously [43], the higher number of phosphorylation sites upregulated than downregulated in response to a kinase inhibitor treatment was probably caused by the length of the treatment (one hour), which was chosen rather to identify signalling pathways rewired by ATR inhibition in irradiated cells than phosphorylations directly dependent on ATR kinase activity in response to IR.

Analysis of all the proteome samples prepared using the same treatment and time interval resulted in quantification of the unmodified fraction of the proteome (phosphorylated peptides were not considered for proteome quantification) and confirmed our expectations that there would be no significant changes of proteome one hour after irradiation combined with VE-821 treatment. As depicted in Fig 3A, the distribution of normalized log_2_ SILAC H/L ratios corresponding to quantification of unmodified proteins was very narrow, with most values distributed close to zero. Moreover, Pearson correlation between the replicates was weak (R 0.24–0.387; Fig 3C), which is more likely caused by non-existing trends in an unperturbed system rather than by an irreproducibility of the analysis.

Pearson correlation between normalized log_2_ SILAC H/L ratios of phosphopeptides and log_2_ normalized SILAC H/L ratios of corresponding proteins was also calculated (the calculation was based on 2,738 phosphorylation sites which represent almost 30% of the data set), and the low correlation coefficient (*R* was -0.013) further confirmed that the observed changes on the phosphoproteome level were not systematically dependent on changes of the abundance of corresponding proteins (Fig 3B).

Taken together, we showed that VE-821 co-treatment significantly affected the phosphorylation response in cells treated with IR, and that the observed changes were not induced by the changes on the proteome level as the proteome remained unperturbed.

### Gene ontology and signalling pathway enrichment analysis of regulated phosphoproteins provided a general description of the VE-821 modulated phosphoproteome

**Fig 4:**
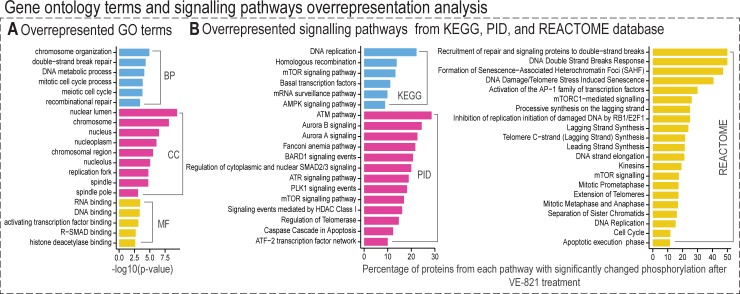
Selected results from gene ontology level 4 terms and signalling pathways overrepresentation analysis. Functional annotation of proteins with phosphorylation sites significantly affected by VE-821 treatment and over-representation analysis were done using ConsensusPathDB overrepresentation-analysis online tool. (A) Overrepresented GO terms (FDR < 0.05, all proteins quantified in our study were used as a statistical background). (B) Regulated phosphoproteins were mapped to signalling pathways from 3 different databases (KEGG, REACTOME, and PID) and pathway-coverage was calculated for overrepresented pathways (FDR < 0.05, all proteins comprised in detected pathways were used as a statistical background).

To functionally classify phosphoproteins quantified in our study, we performed a functional annotation and over-representation analysis using the ConsensusPathDB over-representation analysis web tool [49,50]. A list of 455 phosphoproteins containing VE-821-regulated sites was tested against a custom background reference set derived from our data comprising all phosphoproteins with at least one phosphorylation site quantified in all three biological replicates. As shown in Fig 4A, regulated phosphoproteins were over-represented in the nucleus, specifically in chromosomes, the mitotic spindle, and the replication fork, and were involved in chromatin organization, DNA repair and metabolism, the cell cycle, and regulation of transcription factors.

Using the ConsensusPathDB tool, we also mapped regulated phosphoproteins to signalling pathways from three different pathways databases; pathway coverage was calculated for selected pathways (Fig 4B). The list of pathways containing proteins with VE-821-regulated phosphorylation sites, and thus pathways potentially modulated by VE-821 treatment contained pathways involved in DNA repair, replication, telomere synthesis, apoptosis, regulation of mitosis, transcription factors regulation, chromatin regulation via histones modification, and also pathways primarily related to cellular metabolism.

### Network analysis revealed the complexity of cellular response and multiple functionally-related clusters affected by VE-821

**Fig 5:**
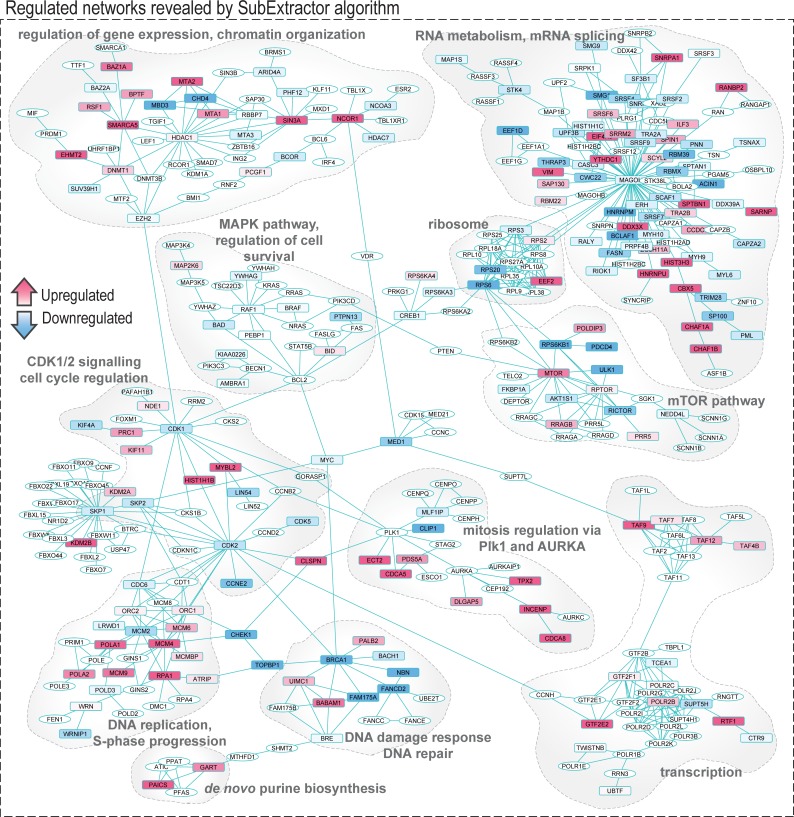
Protein network extracted using SubExtractor algorithm and visualized in Cytoscape. Protein-protein interactions with STRING score above 900 were used as an input for the algorithm and regulated subnetworks were extracted (FDR < 0.005). Proteins in rectangles were identified and quantified in our study; proteins in ellipses were added to the network by the algorithm. Proteins with downregulated phosphorylation are depicted in blue; proteins with upregulated phosphorylations are depicted in pink. The colour intensity corresponds to the degree of regulation (combined z-score).

Using the SubExtractor algorithm [55], we extracted multiple subnetworks of interconnected nodes with the most prominent changes in phosphorylation after VE-821 treatment. The largest subnetwork is depicted in Fig 5. For each group of interconnected nodes, we further performed functional annotation enrichment analysis to better understand its functions. To simplify the interpretation, the clusters were divided into two groups.

The first one can be concisely described as a multi-level stress-induced regulation of gene expression: regulation of RNA biogenesis, its modification and stability, and translation. Specifically, SubExtractor extracted distinct clusters that contain chromatin-modifying enzymes, transcription initiation and elongation complexes, and proteins from the mRNA splicing machinery. VE-821 treatment also affected phosphorylation of ribosomal proteins, suggesting that it might also alter protein synthesis. Furthemore, the ribosomal cluster is tightly linked to a cluster that mostly contains members of the mTOR pathway, indicating that this pathway might be dysregulated by VE-821 treatment, consequently resulting in affected cell growth and proliferation.

The second group mostly contains clusters corresponding to DDR and stress-induced cell cycle regulation. As shown in Fig 5 we detected a densely interconnected cluster composed of members of the minichromosome maintenance complex (MCMs), origin recognition complex subunits (ORCs), and DNA polymerases; this cluster also includes several known ATR-interacting proteins, and corresponds to the regulation of origin firing and S-phase progression by ATR in response to stress. Further hubs in the extracted network comprised four cell cycle regulating kinases–cyclin dependent kinase 1 (CDK1), cyclin dependent kinase 2 (CDK2), serine/threonine kinase PLK1 (PLK1), and aurora kinase A (AURKA)–and their regulated substrates extracted as their interactors. These kinases refer to the disruption of cell cycle checkpoints associated with IR-induced DNA damage and dysregulation of mitosis and cell division by VE-821 treatment as detected by the DNA content analysis. Interestingly, the algorithm also extracted a small cluster containing enzymes from the *de novo* pyrimidine synthesis metabolic pathway connected to the DNA repair cluster suggesting that metabolism of nucleotides might be also affected by ATR inhibition.

Moreover, we identified multiple protein kinases in the centre of extracted modules indicating their possible dysregulation by the treatment. To investigate kinome dysregulation induced by VE-821 treatment, we performed sequence motif analysis and kinase activity analysis together with statistical evaluation of the possible changes.

### Sequence motif and kinase activity analyses confirmed the downregulation of PIKK substrates after VE-821 treatment

**Fig 6:**
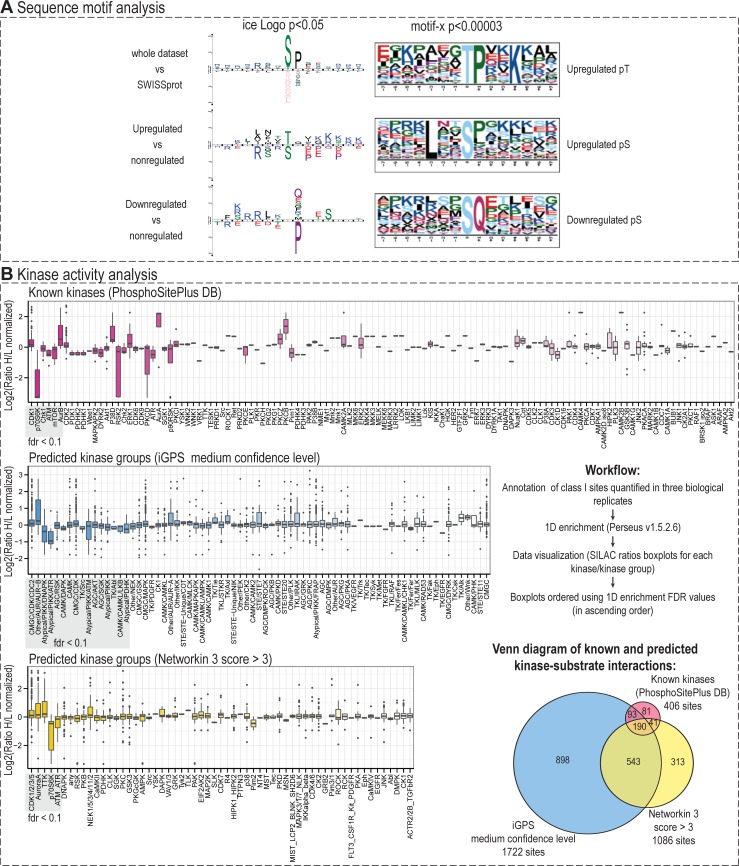
Sequence motif and kinase activity analyses. (A) Sequence motif analysis was performed using IceLogo and motif-x. Amino acid sequences of differentially up- or down- regulated phosphorylation sites were analysed against a statistical background comprising all class I sites quantified in our study. Depicted motifs were found enriched at indicated significance levels. In IceLogo analysis, amino acids that were more frequently observed in the proximity of a regulated phosphorylation site are indicated over the middle line, whereas the amino acids with lower frequency are indicated below the line; phosphorylated amino acid is located at position 0. (B) Kinase activity analysis. Phosphorylation sites were annotated with their known kinases using PhosphoSitePlus database if available. iGPS v1.0 and Networkin 3 were used to predict kinases for all class I sites, and the predictions were filtered as indicated. Venn diagram shows the overlap between phosphorylation sites annotated by each method and applying desired filtering criteria. Boxplots depict SILAC H/L ratios distribution of phosphorylation sites assigned to each kinase/group. The distribution, median, and/or outlier-shifts to positive values indicate a possible upregulation of kinase activity; trends toward negative ratio values show a possible downregulation of kinase activity. Statistical analysis was performed using 1D enrichment analysis in Perseus (v 1.5.2.6), and the test FDR value for each kinase/group was used to rank the boxplots in ascending order. The higher color intensity corresponds to the lower 1D enrichment FDR value and vice versa.

To identify global trends in VE-821 induced phosphoproteome changes, we first analysed and visualized sequence motifs using the iceLogo tool [57] and motif-x algorithm [58]. From the sequences surrounding downregulated sites, an SQ motif was extracted using motif-x, and Q at position +1 was significantly overrepresented among downregulated sites in iceLogo analysis, providing a confirmation of the downregulation of PI3K-related kinases ATM, ATR, and DNA-PK (Fig 6A). Additionaly, 1D enrichment analysis of known and predicted kinase motifs repeatedly indicated that the SILAC H/L ratio distribution of the ATM/ATR group substrates shows a significant declining trend (Fig 6B; an overview of all phosphorylation sites with known kinase and kinase predictions is given in S2 Table; an overview of all sites assigned to PIKKs is given in S3 Table). From these ratio shifts, the activity of the ATM/ATR group might be inferred as downregulated. Even though both kinases share the same phosphorylation motif, both prediction algorithms clearly favour ATM over ATR. We argue that this bias is probably introduced by a better annotation of ATM substrates and protein-protein interactions in the databases.

Nevertheless, using antibody against Chk2 phoshorylated at Thr 68, which is a widely-used marker of ATM kinase activation in response to ATM activating stress, we showed that 10 μM VE-821 did not inhibit ATM signalling in our experiments (Fig 1A). Furthermore, we found additional pieces of evidence of unaffected ATM signalling in our phosphoproteomic data: ATM Ser 2996 autophosphorylation site, which has been previously shown to be rapidly induced by IR [69], was not affected by the treatment. Similarly, two IR-induced autophosphorylation sites of a downstream ATM target Chk2, Ser 260 and Ser 379 [70,71], were also not significantly changed.

On the contrary, known ATR targets located within the Chk1 regulatory domain were observed to be downregulated upon VE-821 treatment: Ser 345 phosphorylation was detected by western blotting (Fig 1A), and Ser 317 phosphorylation was quantified in our phosphoproteomic experiment. An important marker of Chk1 activity, the autophosphorylation site Ser 296, was not detected in our study. Chk1 itself was evaluated as significantly downregulated in the 1D enrichment analysis of the known substrates, providing further evidence of the downregulated ATR/Chk1 pathway. Considering the high selectivity of VE-821 towards ATR kinase (IC50 (ATR): 26 nM, Ki (ATR): 13 nM, Ki (DNA-PK): 2.2 μM, Ki (ATM): 16 μM), we can assume that significant changes within SQ/TQ phosphorylations motifs assigned to the ATM/ATR group can be explained by specifically inhibited ATR kinase activity.

DNA-PK, which is also known to share the glutamine directed phosphorylation motif, was evaluated to be significantly downregulated using the iGPS predictions. However, we did not find any known site of DNA-PK that would be downregulated in our study (the only known DNA-PK phosphorylation site was Ser 430 on Vimentin, which was unchanged). Moreover, 10 μM VE-821 was proven to leave DNA-PK signalling unaffected in a cell-based assay [15], and hence we assume that DNA-PK inhibition is rather unlikely.

In all three experimental replicates, we identified and quantified 21 downregulated SQ/TQ class I phosphorylation sites, eleven of which were known substrates of ATM/ATR kinases or predicted by iGPS or Networkin 3. Most of the downregulated sites were located on proteins with confirmed roles in DDR; however, potential functions of some of them have not yet been elucidated. Importantly, VE-821 treatment altered phosphorylation of MRN and BRCA1-BRCT/Abraxas complexes and several other proteins with a known role in DNA damage response. Examples of the most interesting phosporylation sites and their functions are discussed in detail in the S1 File.

### Dysregulation of cyclin-dependent kinases and aurora kinases was responsible for disruption of the G2/M checkpoint and faster progression through mitosis

In the DNA content analysis, we showed that VE-821 disrupted IR-induced G2/M arrest in MOLT-4 cells (Fig 1I). Cells treated with ATR inhibitor were not able to activate the G2/M checkpoint, which in turn led to faster progression into mitosis with unrepaired IR-induced DNA damage. Such progression has been shown to cause mitotic catastrophe and apoptotic cell death in haematological malignant cell lines [36,72,73]. Using phosphoproteomics, we aimed to describe which kinases were dysregulated by ATR inhibition and thus may contribute to the G2/M checkpoint disruption. In the sequence motif analyses (Fig 6A), we observed proline-directed motifs followed by basic amino acids significantly over-represented in the upregulated dataset, which corresponds well to the known sequence logo of CDK 1 and 2. Futhermore, as depicted in Fig 6B, the kinase activity analysis revealed a possible upregulation of G2/M checkpoint controlling CDK1 kinase, mitotic kinases aurora A and B, and kinases from the NEK family, particularly serine/threonine protein kinase NEK2 (NEK2). An overview of detected substrates of mitotic kinases is given in S3 Table.

The kinase activity analysis confirmed a significant upregulation of cyclin dependent kinases, predominantly CDK1 (CDC2), with a median ratio only slightly deviated to positive values, but numerous outliers strongly upregulated after VE-821 pre-treatment (Fig 6B). Many of these outliers were classified as so-called “regulatory” sites (*i*.*e*. sites with previously discovered and experimentally validated effects on the modified protein) with either a known function in mitosis, or whose function has not yet been elucidated, but whose corresponding phosphoproteins have been shown to have important roles in the onset of mitosis and mitotic progression. Examples of the most interesting CDK1-phosphorylated sites and their functions are given in the S1 File. Additionally, unscheduled CDK1 activity in G1 phase has been shown to trigger apoptosis in X-irradiated MOLT-4 cells [74]. In our data, VE-821 significantly affected viability of MOLT-4 cells. It is possible that in addition to G2/M checkpoint disruption and induction of post-mitotic cell death, the dysregulation of CDK1 by ATR inhibition contributes to the increased rate of apoptosis in MOLT-4 cells.

Kinases from the Aurora kinase family—Aurora kinases A and B—are master regulators of mitosis progression and onset of cytokinesis, whose activities require tight spatial and temporal regulation, and their dysregulation could cause errors in mitosis, faster progression through the abscission checkpoint, and affect postmitotic genome surveillance. In our analysis, we found that the activities of these two kinases might be upregulated by VE-821 treatment (Fig 6B). Examples of the most interesting phosphorylation sites are given in the S1 File.

Taken together, our data indicate that ATR inhibition induced dysregulation of the main mitotic kinases. The results are in concordance with our previous study on HL-60 leukemic cells [43], in which we observed dysregulation of CDK1, PLK1, and NEK2.

### The key regulator of cellular metabolism, Serine/threonine protein kinase mTOR and its downstream effector, p70S6K, were inhibited by an off-target effect of 10 μM VE-821 treatment, possibly contributing to the growth inhibition observed in the proliferation assays

**Fig 7:**
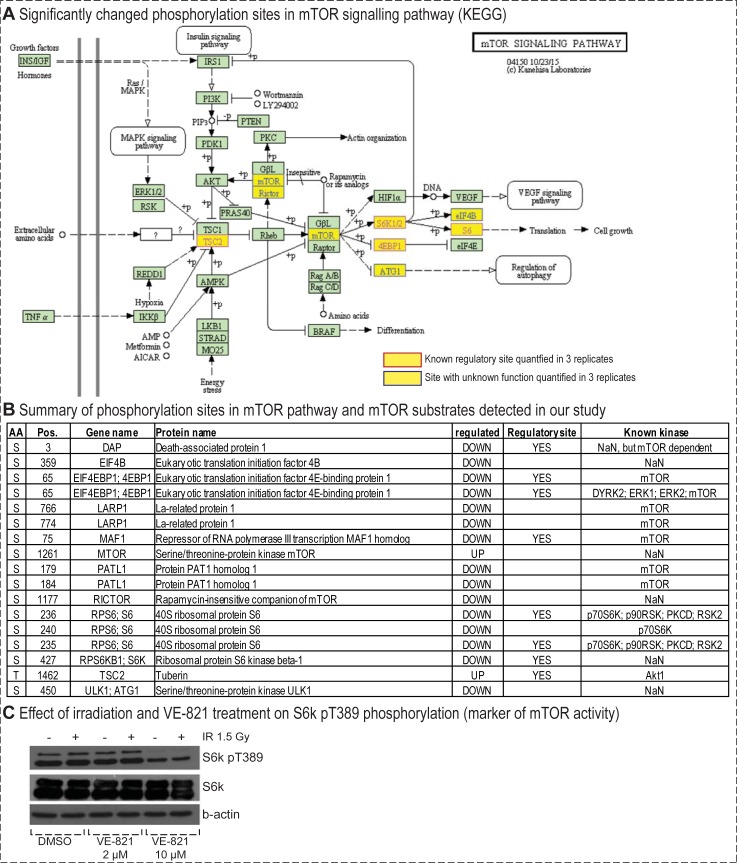
Significant changes in mTOR signalling pathway. (A) Proteins with significantly changed phosphorylation sites were mapped onto KEGG signalling pathways. (B) Summary of all phosphorylation sites comprised in “mTOR signalling pathway” pathway in KEGG database and phosphorylation sites that are known mTOR substrates or known to be mTOR-dependent. (C) Immunoblotting analysis of mTOR activity. Cells were collected 2 hour after IR (1.5 Gy). Activity of mTOR was monitored via the detection of its target pT389 of p70S6K (S6K, RPS6KB1). The expression state of p70S6K was also evaluated. β-actin expression was analysed as a loading control.

As the previous data analyses already indicated, we found a significant downregulation of the serine/threonine protein kinase mTOR (mTOR) in the kinase activity analysis. The protein kinase mTOR is the principle regulator of cellular metabolism promoting anabolic processes and inhibiting catabolic processes such as autophagy [75]. In total, we found seven known direct mTOR targets downregulated in our study and several other proteins included in the mTOR signalling pathway from the KEGG pathway database (depicted in Fig 7A and summarized in Fig 7B). Detailed description of the functions of the most interesting mTOR related phosphorylation sites and their functions are given in the S1 File. Most of the mTOR-phosphorylated or downstream regulated proteins are involved in transcription and translation regulation and thus contribute to regulation of cell growth and proliferation: Eukaryotic translation initiation factor 4E-binding protein 1 (EIF4EBP1; Ser 65), protein PAT1 homolog 1 (PATL1; Ser 179 and Ser 184) and la-related protein 1 (LARP1; Ser 766 and Ser 774), and repressor of RNA polymerase III transcription MAF1 homolog (MAF1). In addition to proteins involved in translation regulation, we also identified significantly changed phosphorylation of two phosphoproteins linked to autophagy: death-associated protein 1 (DAP1) and serine/threonine-protein kinase ULK1 (ULK1). These observations might indicate that mTOR inhibition by 10 μM could further affect the phenotype observed after irradion via suppression of autophagy [76].

To further validate the VE-821-mediated mTOR inhibition, we performed a confirmation experiment using an antibody detection of a regulatory ribosomal protein S6 kinase beta-1 (p70s6K) Thr 389 phosphorylation site that is commonly used to examine the activity of mTOR. As shown in Fig 7C, while irradiation by a dose of 1.5 Gy did not affect p70S6K phosphorylation, mTOR-dependent p70S6K phosphorylation was almost diminished after 10 μM VE-821 treatment in both irradiated and sham-irradiated cells. However, 2 μM VE-821 did not have any significant effect on the Thr 389 phosphorylation site.

The inhibition of mTOR by 10 μM VE-821 but not by 2 μM treatment is coherent with the results of our proliferation and DNA analysis experiments, and explains the difference we observed between the 2 μM and 10 μM VE-821 treated groups. While both concentrations of the inhibitor radiosensitized MOLT-4 cells, 10 μM had much stronger inhibitory effect on the number of viable sham-irradiated cells (Fig 1B and 1F). Likewise, while both concentrations caused the disruption of the IR-induced G2/M arrest, it was only the higher concentration which caused a significant accumulation in G1 phase in sham-irradiated cells (Fig 1I). These conclusions are supported by the fact that mTOR inhibition triggers the G1 metabolic checkpoint and inhibits cell growth and proliferation [77]. The mTOR inhibition by 10 μM VE-821 might also explain the high sensitivity of the MOLT-4 cell line to VE-821 treatment. Consistently with MOLT-4 homozygous deletion of *PTEN* [30], it has been shown that *PTEN*-deficient tumours were more likely to be sensitive to mTOR inhibition [78,79].

In addition to the downregulation of mTOR, we also observed a significant downregulation of the p70S6K (or RPS6KB1) in all kinase activity analyses (Fig 6B). P70S6K is an important kinase that acts downstream of mTOR to promote protein synthesis and cellular proliferation [80,81]. In addition to the western blotting detection of changes in Thr 389 phosphorylation (Fig 7C) induced by VE-821 treatment, we also detected another downregulated mTOR-dependent “regulatory” phosphorylation site (Ser 427) in our phosphoproteomic data. From the known p70S6K substrates, we detected three strongly downregulated phosphorylation sites on 40S ribosomal protein S6 (RPS6)–Ser 235, Ser 236, and Ser 240. These phosphorylation sites are phosphorylated by ribosomal kinases to initiate translation [82], and thus the inhibition of their phosphorylation causes inhibition of protein synthesis. Moreover, iGPS and Networkin 3 predicted two more members of the translation machinery to be downregulated substrates of RSKs—eukaryotic translation initiation factor 3 subunit A (EIF3A) Ser 584 and eukaryotic translation initiation factor 4B (EIF4B) Ser 119, providing further evidence for translation inhibition caused by inhibitor treatment.

### The downregulation of dihydroorotase phosphorylation, a substrate of p70S6K, showed a possible link to nucleotide synthesis alteration induced by VE-821 treatment

Surprisingly, on the list of known/predicted p70S6K substrates detected in our study, we did not find phosphoproteins involved solely in protein synthesis. Dihydroorotase (CAD) is an enzyme required for the first steps of *de novo* pyrimidine synthesis. Phosphorylation on Ser 1859 by p70S6K downstream of mTOR stimulates dihydroorotase activity of CAD to induce pyrimidine synthesis [83]. Ser 1859 was detected to be downregulated in our study, suggesting that VE-821 treatment might also hamper synthesis of nucleotides, which are necessary for both cellular proliferation and DNA repair after DNA damage induction.

Possible VE-821-induced nucleotide synthesis dysregulation was striking enough to encourage us towards further data examination and to search for regulated phosphorylation sites located on enzymes included in nucleotide synthesis pathways. Indeed, in all three replicates, we identified two additional regulated phosphorylation sites on proteins from the *de novo* purine synthesizing pathway: phosphoribosylglycinamide formyltransferase (GART) Tyr 348 and phosphoribosylaminoimidazole carboxylase (PAICS) Ser 27; the latter has been identified as a potential substrate of CDKs in two high-throughput phosphoproteomic screenings [84,85]. However, none of these enzymes has been proven to be regulated by phosphorylation, and thus we can only speculate whether these two phosphorylation events might have some effect on *de novo* purine synthesis or not.

### Targeted metabolomic analysis of VE-821 treated MOLT-4 cells resulted in detection of 206 intermediary metabolites

In the phosphoproteomic analysis of VE-821-treated and irradiated cells, changes in the mTOR activity were detected as a result of an off-target effect of the inhibitor. As mTOR is the principal regulator of cellular metabolism [75], this finding raised an intriguing question–whether the inhibitor treatment modulates cellular metabolism after irradiation. We further examined our data and found several metabolic enzymes containing phosphorylation sites affected by VE-821. In addition to the aforementioned enzymes involved in the metabolism of purine and pyrimidine, we detected changes in enzymes from the metabolism of glucose, namely phosphofructokinase 1 (Ser 775), triosephosphate isomerase (Thr 28), phosphoglycerate mutase 1 (Ser 189), and pyruvate dehydrogenase E1 component subunit alpha (Ser 293 and Ser 300).

The metabolic responses of different cell lines to various doses of IR have been investigated in several studies, for instance [86–88]. Briefly, these studies showed that IR-triggered production of oxygen free radicals by radiolysis of water induced changes in glutathione level, disturbed energy metabolism resulting in a rapid decrease of cellular ATP level, and increased levels of free amino acids, choline and lipids, which are produced by degradation of damaged proteins and membranes. However, to our best knowledge, no study has yet been published investigating the modulation of this response by small molecular kinase inhibitors of any of the PIKK family. Therefore, we performed a metabolomic analysis of irradiated MOLT-4 cells, whose response to IR was modulated by inhibition of ATR (and, as shown above, mTOR) using 10 μM VE-821.

**Fig 8:**
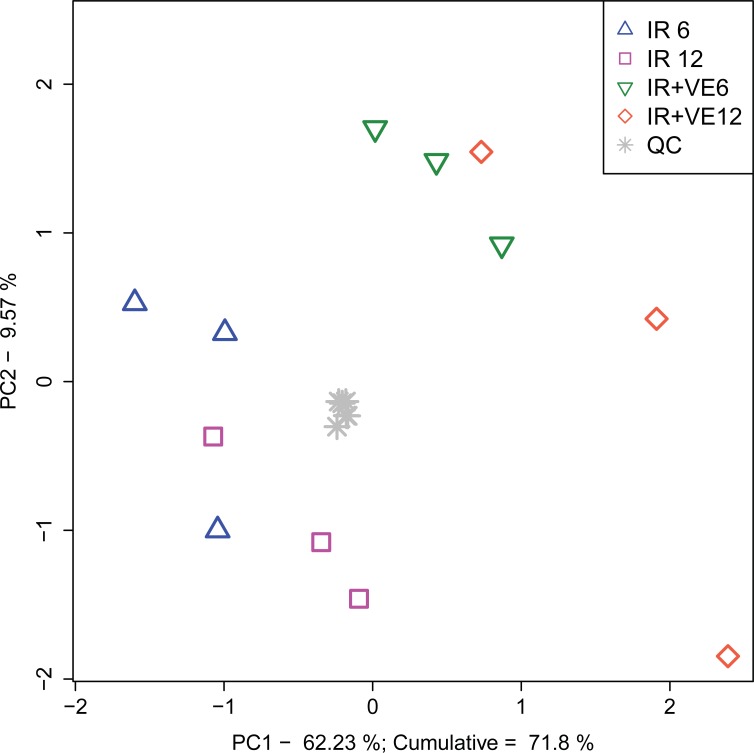
Principal component analysis score plot of VE-821 induced metabolome changes in irradiated MOLT-4 cells. IR–irradiated cells pre-treated with DMSO, IR+VE–irradiated cells pre-treated with 10 μM VE-821, both groups were analysed 6 and 12 hours after irradiation. Grey stars represent quality control samples.

Metabolite profiling performed by a label-free targeted analysis of cellular extracts collected 6 and 12 h after irradiation (Fig 2A) resulted in detection of 206 intermediary metabolites (Fig 2D). As shown in Fig 8, PCA revealed clear separation in metabolic profiles between both treatment groups and controls with explained variance of 72%. Localization of samples in PCA indicated that the inhibitor had a more significant impact on the group clustering than the incubation time.

The detected metabolites were further mapped on KEGG pathways to visualize metabolic pathways affected by the inhibitor 6 and 12 hours after irradiation (S2 File). This analysis showed that the main changes in the metabolome of the VE-821-treated MOLT-4 cell line occurred in a group of metabolites involved in the cellular antioxidant system, glycolysis and the citrate cycle. Differences in levels of nucleosides, nucleotides, deoxynucleotides, free amino acids, and acylcarnitines were also observed.

### Treatment with VE-821 potentiated changes in cellular antioxidant defence, degradation of proteins and membranes, and mitochondrial damage induced by IR

**Fig 9:**
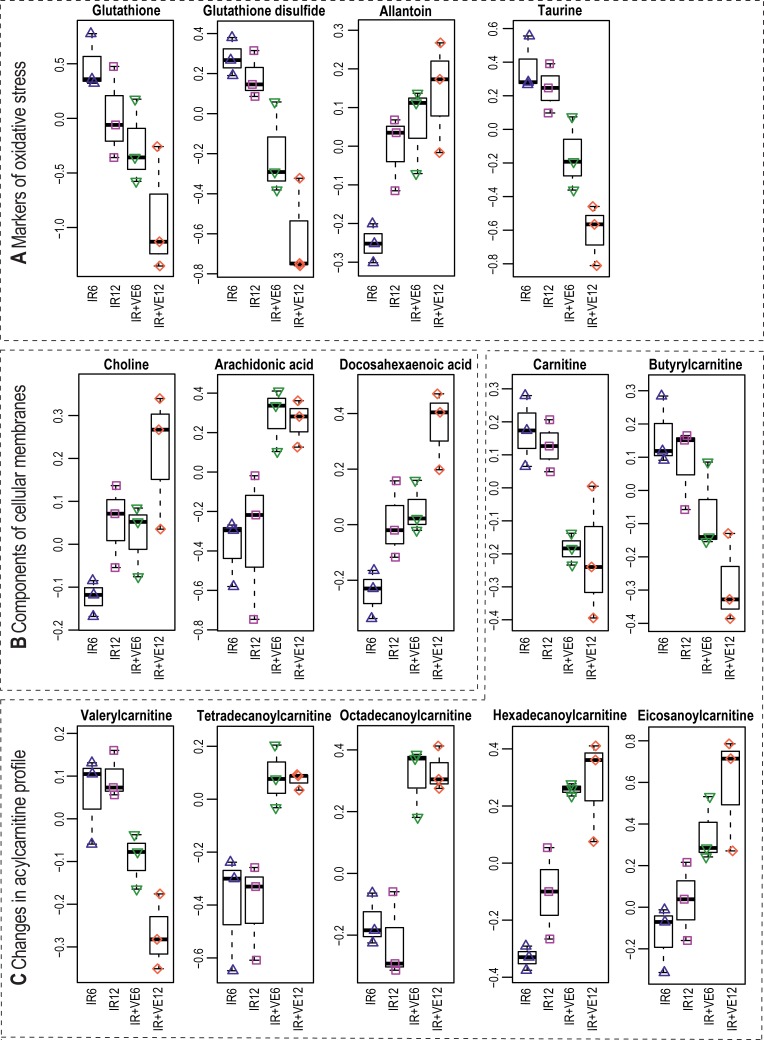
VE-821 induced changes in cellular response to oxidative stress, degradation of membranes, and acylcarnitine profile. (A) Boxplots of selected markers of oxidative stress, (B) selected components of cellular membranes, and (C) selected changes in acylcarnitine profile are depicted. IR–irradiated cells pre-treated with DMSO, IR+VE–irradiated cells pre-treated with 10 μM VE-821; both groups were analysed 6 and 12 hours after irradiation.

**Fig 10:**
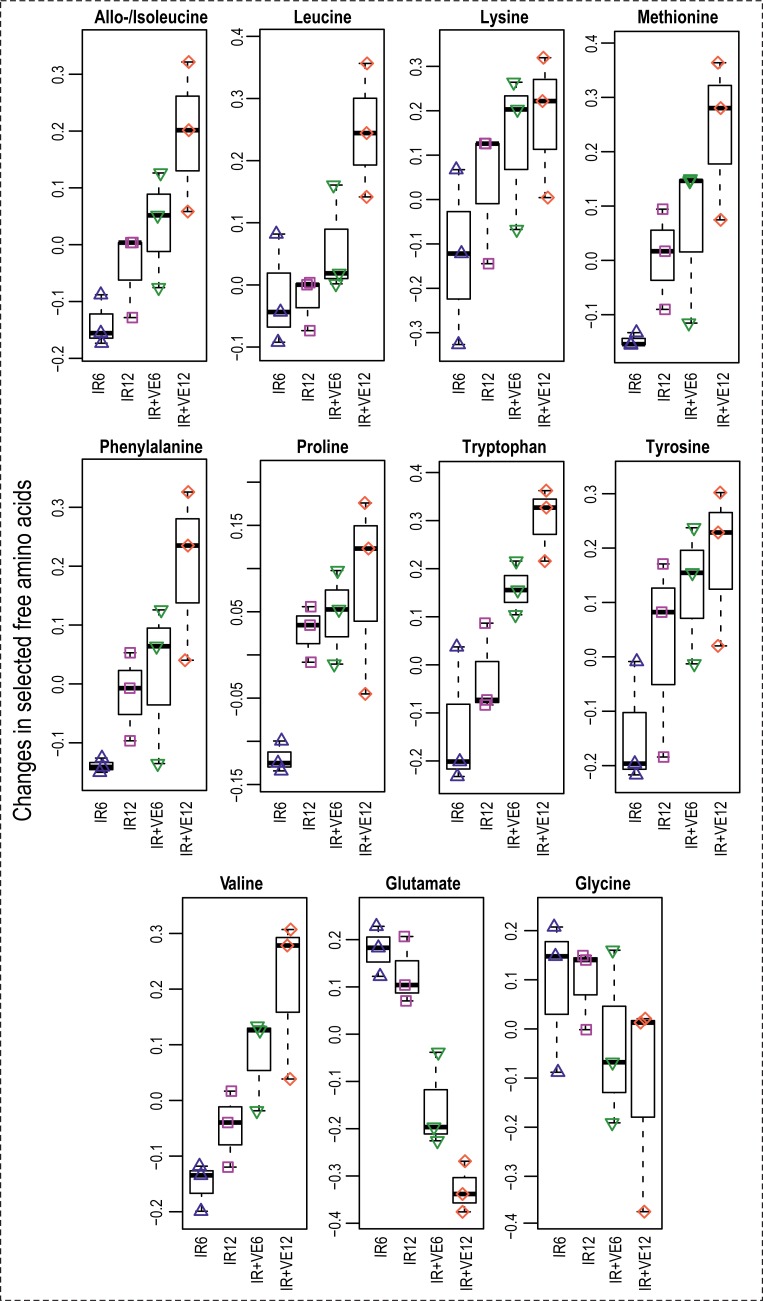
Changes in free amino acids levels in irradiated and VE-821 treated cells. Boxplots of selected amino acids in irradiated cells pre-treated with DMSO (IR) and irradiated cells pre-treated with 10 μM VE-821 (IR+VE); both groups were analysed 6 and 12 hours after irradiation.

When cells are exposed to IR, cellular structures can be damaged by ionization directly by deposition of energy, but also indirectly by water radiolysis and stimulation of nitric oxide synthases and NADPH oxidases [89]. Reduced glutathione (GSH) is an important cellular antioxidant and the reduced/oxidized glutathione ratio (GSH/GSSG) is a sensitive biomarker of oxidative stress [90]. It has been reported previously that IR induced a significant decrease in cellular glutathione level [86,87]. In our study, we observed a decrease in GSH over time and this decrease was amplified by inhibitor treatment (Fig 9A). However, GSSG was also depleted, as well as important precursors of GSH biosynthesis (glutamate and glycine, Fig 10). Therefore, we assume that the overall decrease in the GSH/GSSG pair was caused by increased turnover of GSH and simultaneously missing precursors. Additionally, we observed increased levels of allantoin and decreased levels of taurine (Fig 9A). Allantoin can be formed in the presence of ROS by non-enzymatic reactions, and similarly to GSH, it can be used as a marker of oxidative stress [91]. Taurine is an intermediate in metabolism of methionine and an anti-oxidant, and its decrease might indicate its elevated consumption in VE-821 treated cells as a part of preventive mechanisms counteracting oxidative stress. Taken together, our data provide indirect evidence that the cellular response to oxidative stress induced by IR might have been affected by the pre-incubation with VE-821.

In addition to the changes in the cellular redox system, direct effects on proteins and membranes were also observed. Increased levels of several amino acids (phenylalanine, proline, tryptophan, tyrosine, valine, isoleucine, glutamate, glycine, and methionine) can indicate increased turnover of proteins which were damaged by ROS/RNS exposure (Fig 10). Elevated levels of docosahexaenoic acid, arachidonic acid, and choline may be evidence for increased membrane degradation induced by the inhibitor treatment (Fig 9B). IR induces autophagy by inhibition of mTOR [92] probably via a pathway involving ATM and AMPK [93]. VE-821 10 μM, but not 2 μM inhibited mTOR in both sham-irradiated and irradiated MOLT-4 cells (Fig 7C), and we also detected several significantly changed phosphorylations in autophagy-regulating proteins. Thus, we can hypothesize that the treatment might have induced degradation of damaged structures (presumably via autophagy), resulting in increased accumulation of degradation products in cells.

We further observed prominent changes in the acylcarnitine profile (Fig 9C), which might indicate mitochondrial disturbances induced by VE-821. Increased levels of long-chain acylcarnitines (tetradecanoyl-, palmitoyl-, and stearoylcarnitine) and decreased levels of free carnitine, isovaleryl-/ methylbutyrylcarnitine, and malonyl-/ 3-hydroxybutyrylcarnitine were observed. Acylcarnitine changes have been already observed in two independent studies investigating metabolic effects of IR [88,94]. Moreover, it has been shown that l-carnitine acts as a free-radical scavenger protecting irradiated mice from oxidative damage in response to IR [95]. Our observation of the lipid changes in cellular membranes could be explained by lipid peroxidation induced by increased ROS generation in VE-821 treated cells, which was evidenced above as decreased GSH and altered allantoin and taurine levels. However, it would be necessary to perform a lipidomic analysis to specifically address oxidation of lipids induced by IR and VE-821 [96].

### VE-821 potentiated the energy metabolism disruption induced by IR

**Fig 11:**
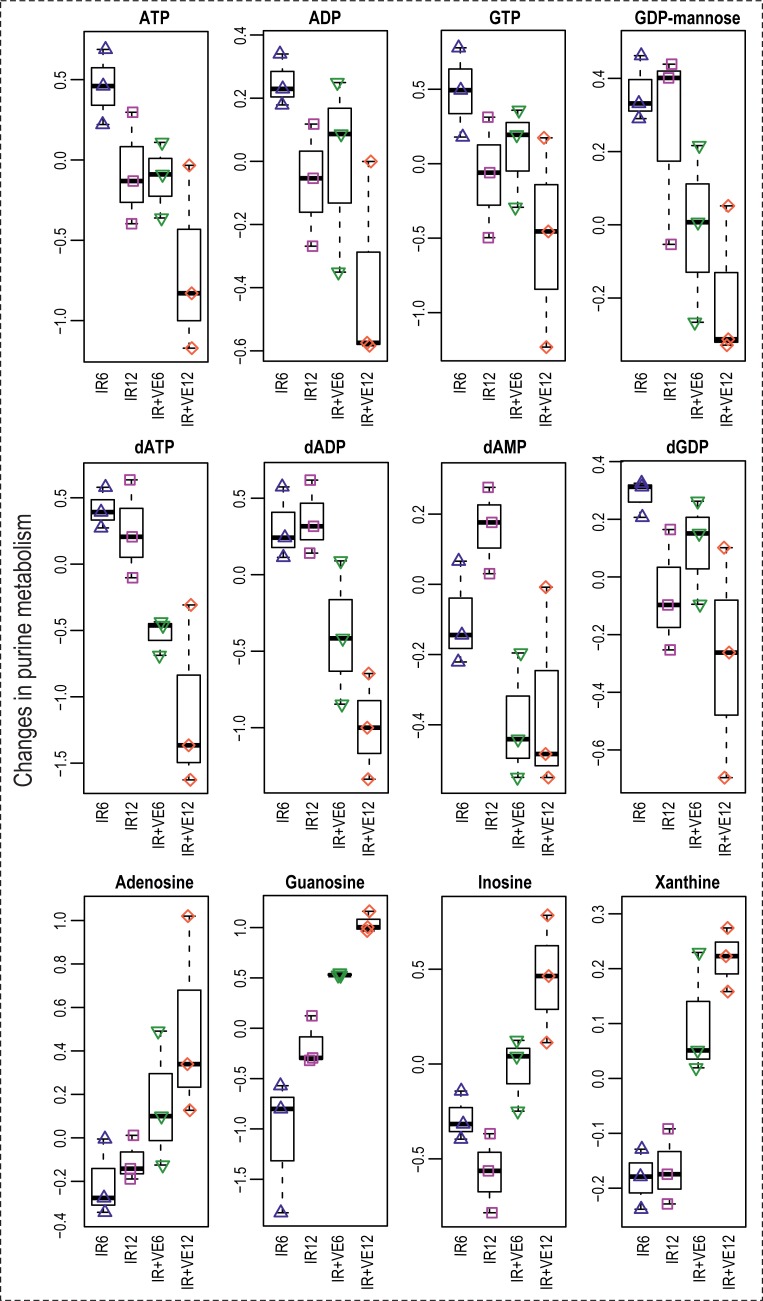
Changes in purine metabolism induced by VE-821 in irradiated cells. Boxplots of selected nucleoside and deoxynucleoside triphosphates, diphosphates, diphosphate derivatives ribonucleosides, and bases from the metabolism of purine are shown. IR–irradiated cells pre-treated with DMSO, IR+VE–irradiated cells pre-treated with 10 μM VE-821; both groups were analysed 6 and 12 hours after irradiation.

**Fig 12:**
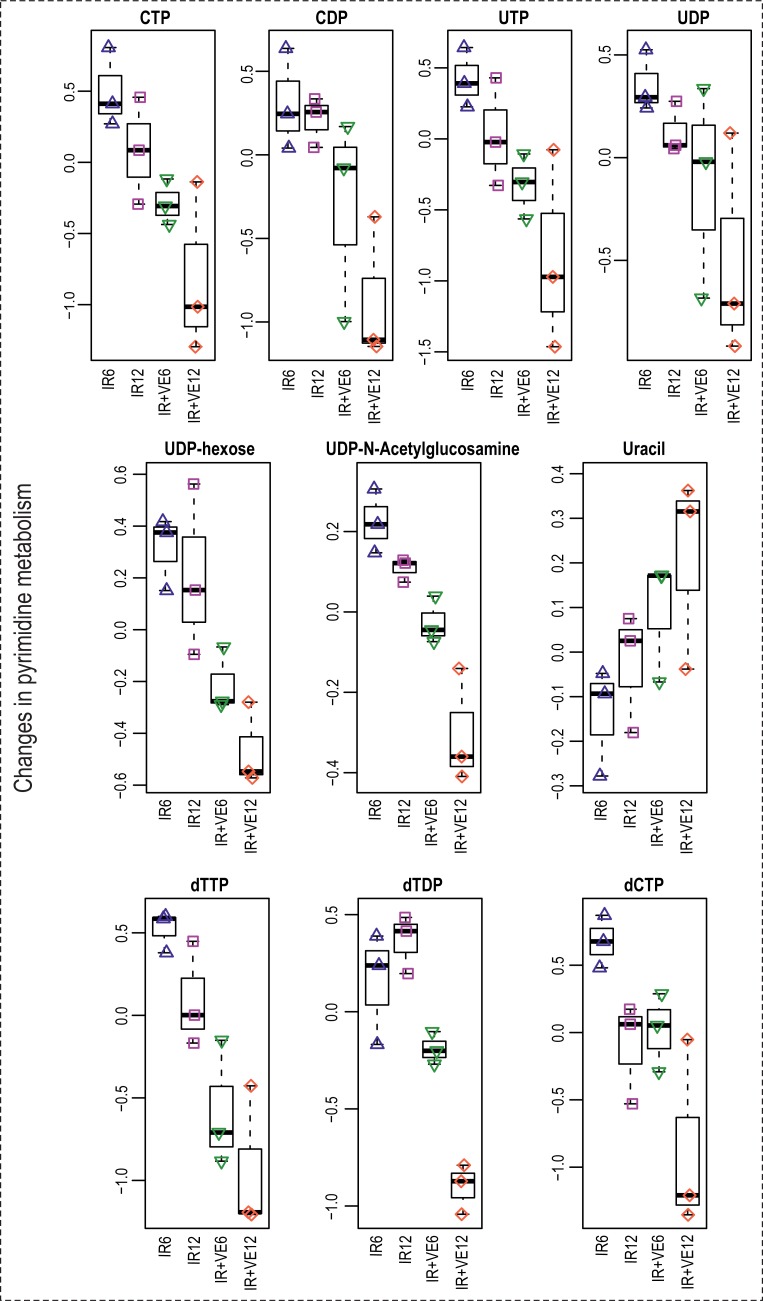
Changes in metabolism of pyrimidine induced by VE-821 in irradiated MOLT-4 cells. Boxplots of selected nucleoside and deoxynucleoside triphosphates, diphosphates, diphosphate derivatives ribonucleosides, and bases from the metabolism of purine are shown. IR–irradiated cells pre-treated with DMSO, IR+VE–irradiated cells pre-treated with 10 μM VE-821; both groups were analysed 6 and 12 hours after irradiation.

IR has been shown to disturb energy metabolism, which was demonstrated by a rapid decrease of cellular ATP levels in irradiated cells [86,97] and serum glucose levels in irradiated mice [98]. In our study, we found decreased levels of nucleoside triphosphates (NTPs; ATP, GTP, CTP, and UTP), diphosphates (NDPs; ADP and UDP), and diphosphate derivatives (UDP-hexoses, GDP-hexoses, and UDP-N-acetylglucosamine), and by contrast increased levels of ribonucleosides (adenosine, guanosine, and pseudouridine) and bases (uracil). The overview of changes in purine and pyrimidine metabolism induced by VE-821 is given in Figs 11 and 12.

Decreased levels of high-energy phosphorylated compounds might indicate disruption in energy metabolism induced by exposure to IR. Importantly, VE-821 amplified the energy depletion caused by IR. It is not clear from our data whether the observed time-dependent decrease is due to increased utilization of NTPs needed as “molecular fuel” for cellular signalling, reparation processes, and programmed cell death following DNA damage induction by IR, or to their decreased synthesis following damaged ATP synthesis in mitochondria.

**Fig 13:**
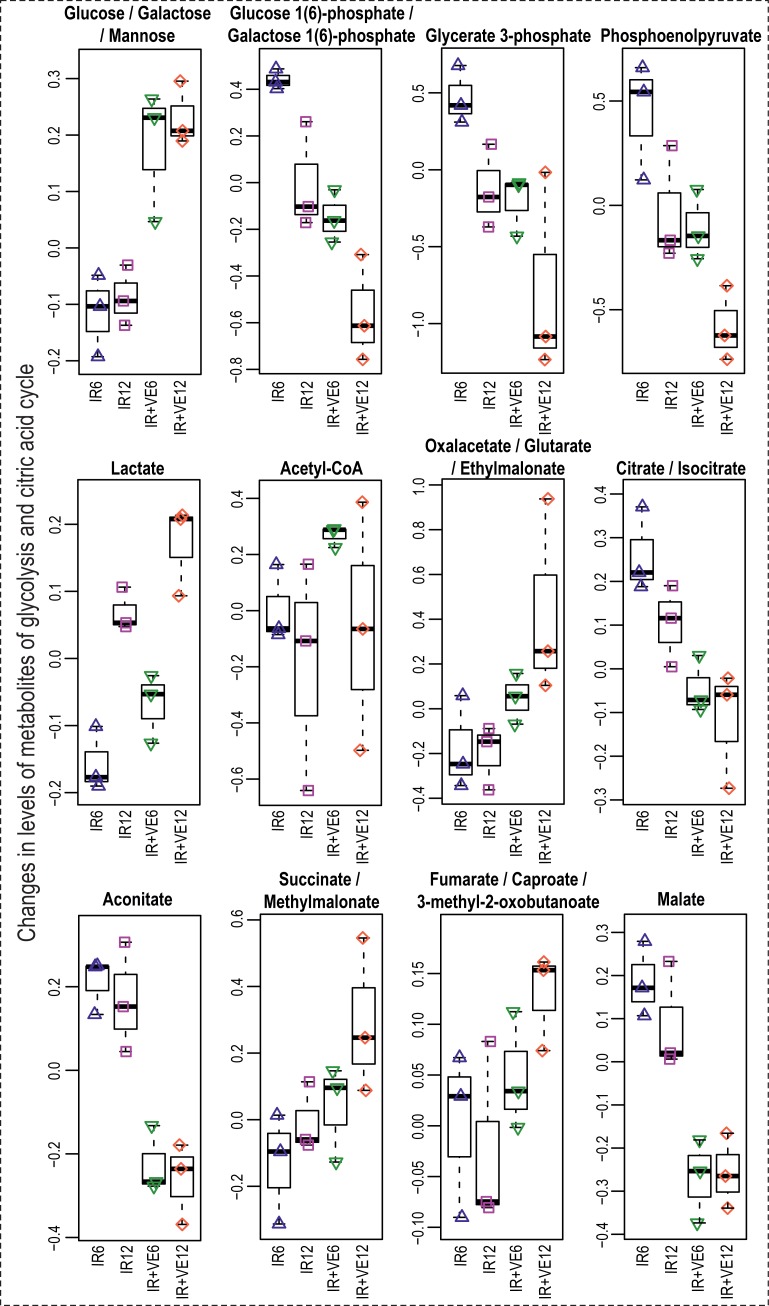
Boxplots of selected metabolites included in metabolism of glucose and citric acid cycle. IR–irradiated cells pre-treated with DMSO, IR+VE–irradiated cells pre-treated with 10 μM VE-821; both groups were analysed 6 and 12 hours after irradiation.

**Fig 14:**
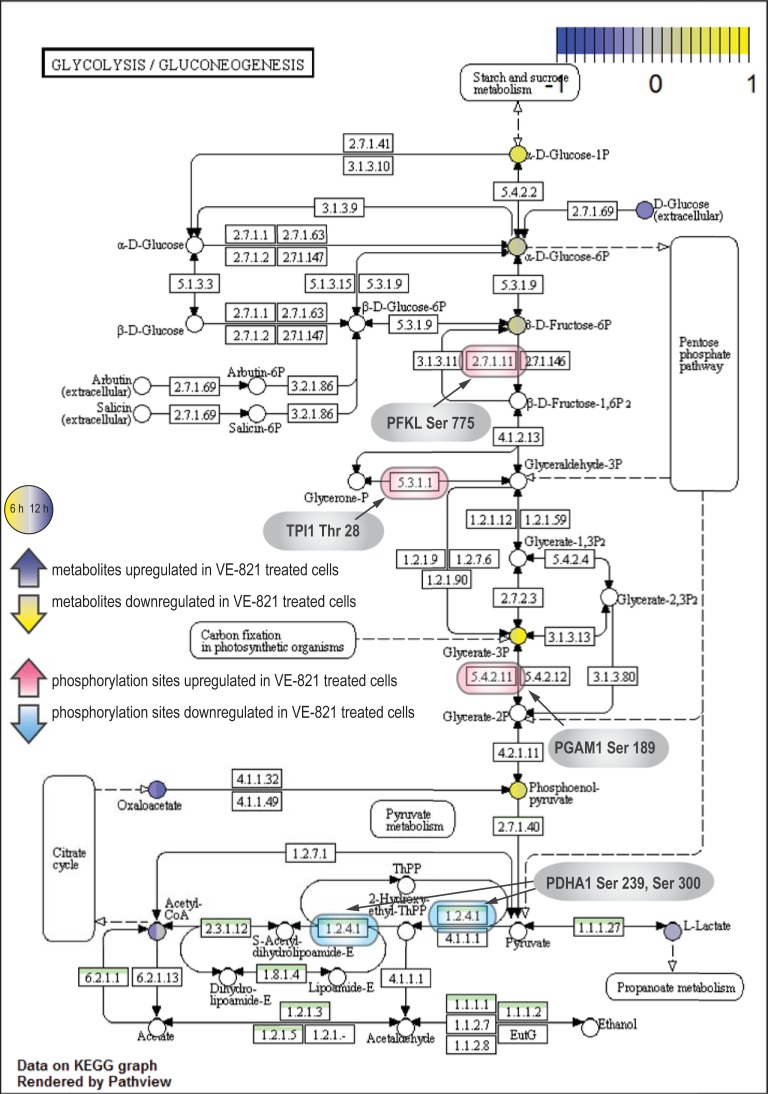
Metabolomic and phosphoproteomic changes detected in metabolism of glucose. Metabolite changes were visualized in KEGG pathway maps. Purple and yellow circles represent metabolites upregulated and downregulated, respectively, in response to VE-821 treatment. Left and right halves of the indicated metabolites show changes 6 and 12 hours after irradiation, respectively. Enzymes containing phosphorylation sites affected by VE-821 treatment are indicated by red and blue rounded rectangles.

Significant changes were also found in glycolysis (Figs 13 and 14). Decreased intermediates of glycolysis can be interpreted as preferential utilization of glycolysis. This hypothesis could be supported by pronounced time-dependent increase in lactate in irradiated control and VE-821-treated cells. We also detected an activating phosphorylation on phosphofructokinase 1 (PFKL) Ser 775 in two experimental replicates of our phosphoproteomic analysis, which has been previously shown to be responsive to insulin treatment and activating glycolysis [99]. This suggests that the metabolic flux through glycolysis might be increased one hour after irradiation.

We further observed significant modulation of citric acid cycle metabolites (Fig 13). Markedly lower levels of aconitate, malate, and citrate/isocitrate were found in VE-821 treated cells; moreover, citrate/isocitrate showed a time-dependent decrease in irradiated cells without any inhibitor treatment. On the contrary, succinate and fumarate were slightly increased in VE-821 treated cells. We assume that these intermediates could have been replenished by anaplerotic reaction from the amino acids obtained by protein degradation.

### VE-821 affected deoxynucleotide pools in irradiated cells

Decreased levels of deoxynucleoside diphosphates (dNDPs; dADP and dTDP) and triphosphates (dNTPs; dATP, dGTP, and dTTP) were found in VE-821 treated irradiated cells. dCTP was also decreased as a result of VE-821 pre-incubation; moreover, time-dependent reduction was observed in irradiated control cells. We also found several purine and pyrimidine bases and ribosides (xanthine, inosine, cytidine, uridine, and thymidine) elevated in VE-821 treated cells. Besides that, nucleoside monophosphates (AMP and GMP) were slightly reduced (Figs 11 and 12).

Deoxyribonucleotides arise either via *de novo* pathways or preferably, by salvage pathways involving reutilization of nucleosides and nucleobases [100]. Thus, in principle, the deoxynucleotide levels might have decreased either by downregulation of their *de novo* synthesis or due to their degradation and downregulation of the salvage pathways. Importantly, dNTPs damaged by IR and oxidised due to increased levels of ROS in irradiated cells are degraded by hydrolysis, largely contributing to dNTP depletion upon irradiation.

As discussed above, in our study, we observed a time-dependent decrease in NDPs and NTPs, i.e. the precursors of dNDPs and dNTPs in *de novo* pathways, which was further amplified by VE-821 inhibitor, supporting the hypothesis that the decrease in dNTPs may have been induced by insufficiency of the precursors for their *de novo* synthesis. Furthermore, we also detected a significantly decreased stimulating phosphorylation on CAD Ser 1859, an important regulator of *de novo* pyrimidine synthesis, and two phosphorylations on two enzymes involved in *de novo* purine biosynthesis (GART and PAICS) with unknown functions. The salvage pathways could have been affected also by the inhibitor treatment; this idea correlates with a recent study showing that activity of deoxycytidine kinase (dCK) is ATR-dependent after stress induction [101]. dCK catalyses the first and rate limiting step of the deoxyribonucleotide salvage pathway as a consequence of its broad substrate specificity [102,103]. Beyaert *et al*. investigated the dCK activity dependency on ATM and ATR after different stress-inducing stimuli. VE-821 significantly reduced upregulation of dCK induced by various genotoxic agents. Importantly, after irradiation, dCK activity was dependent on ATR only in ATM-deficient cells [101]. However, it is necessary to note that the authors examined short time-intervals after irradiation (two hours), when ATM signalling is predominant. It is possible that after longer incubation with the inhibitor, VE-821 might inhibit dCK even in irradiated cells with a functional ATM pathway. Notably, the basal activity of dCK has been shown to be significantly downregulated by ATR silencing [101].

Taken together, it is obvious that several mechanisms could contribute to dNTP depletion upon IR in VE-821 treated cells. Further validation experiments would be necessary to reveal the exact mechanism.

## Conclusion

**Fig 15:**
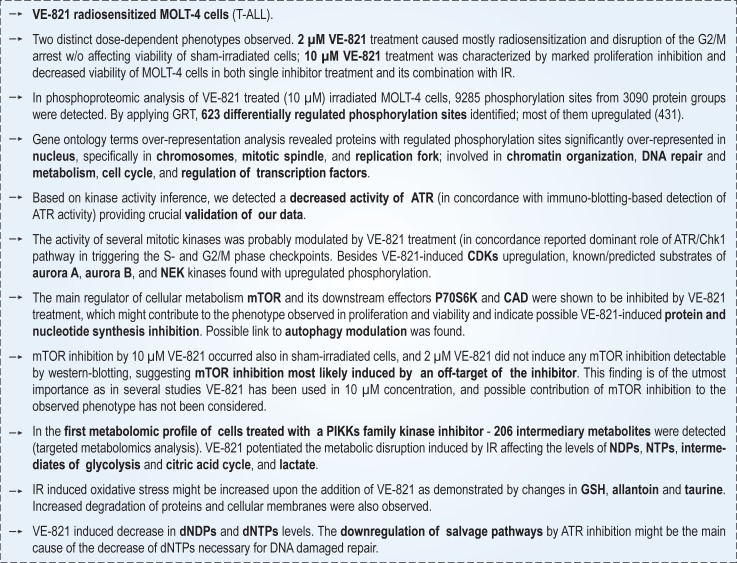
Summary of the most important findings revealed in the presented study.

In recent years, much effort has been focused on discovery and development of tumour specific treatment, which would specifically target only cancer cells and not affect the normal tissues, taking advantage of the tumour specific abnormalities in DDR. In the presented study, we aimed to describe molecular mechanisms underlying radiosensitization of the MOLT-4 cell line (T-ALL) via inhibition of ATR by a potent and specific small molecule inhibitor–VE-821. A brief overview of the most important findings is given in Fig 15.

We employed high-resolution MS to identify and quantify changes in the proteome, phosphoproteome, and metabolome of irradiated VE-821-treated cells. In response to the inhibitor treatment, we revealed changes not only in DDR related pathways and kinases, but also in pathways and kinases involved in maintaining cellular metabolism. Notably, we found downregulation of mTOR, the main regulator of cellular metabolism, which was also confirmed by antibody detection. Although there are multiple signalling pathways interconnecting IR-induced DDR and mTOR regulation that might be potentially affected by ATR inhibition, we assumed that in our case the downregulation was most likely caused by an off-target effect of VE-821 at 10 μM concentration. We further concluded that mTOR inhibition could be one of the factors contributing to the phenotype we observed after treating MOLT-4 cells with 10 μM VE-821, which was different from that observed after treatment with an mTOR non-inhibitory 2 μM concentration.

Targeted metabolomic analysis showed that VE-821 potentiated both IR-induced metabolic disruption and oxidative stress. Our data also indicate that in response to IR, recovery of dNTPs might be affected by VE-821, leading to dNTP deficiency possibly hampering DNA repair.

To our best knowledge, no study has yet been published investigating the modulation of cellular metabolism by a small molecular kinase inhibitor of any of the PIKK family kinases. Nevertheless, even using a highly specific inhibitor might lead to a complex response and similar MS-based phosphoproteomic and metabolomic studies are well-suited to reveal additional information on off-target effects and provide insights into other, possibly non-reported, regulatory mechanisms.

## Supporting information

S1 File Supplemetary discussion. The most interesting substrates of kinases discussed in this paper are described.
(PDF)

S2 File KEGG pathway maps visualization. Metabolite changes were visualized in KEGG pathway maps. Purple and yellow circles represent metabolites upregulated and downregulated, respectively, in response to VE-821 treatment. Left and right halves of the metabolites show changes 6 and 12 hours after irradiation, respectively.
(PDF)

S1 Table Overview of phosphorylation sites identified in all three replicates. (XLSX)

S2 Table Overview of known kinase-substrate interactions and kinase prediction results. (XLSX)

S3 Table Overview of phosphorylations sites assigned to selected kinases or kinase groups. (XLSX)
