# Supplementary material for: Radio-sensitizing effects of VE-821 and beyond: Distinct phosphoproteomic and metabolomic changes after ATR inhibition in irradiated MOLT-4 cells
Source: PLoS One. 2018 Jul 12;13(7):e0199349. doi: 10.1371/journal.pone.0199349 (PMC6042708; doi:10.1371/journal.pone.0199349)
Supplement: S1 File — The most interesting substrates of kinases discussed in this paper are described. (PDF) [file pone.0199349.s001.pdf]

Overview of CDK1 substrates significantly affected by VE-821 treatment in irradiated MOLT-4 cells

Sequence motif analysis showed overrepresentation of phosphorylated amino acids followed by proline and basic amino acids – a well-known motif of cyclin dependent kinases. And indeed, the kinase activity analysis confirmed a significant upregulation of cyclin dependent kinases, predominantly CDK1 (or CDC2), with a median ratio only slightly deviated to positive values, but numerous outliers strongly upregulated after VE-821 pre-treatment. Many of these outliers were classified as so-called "regulatory" sites (*i.e.* sites with previously discovered and experimentally validated effects on the modified protein) with either a known function in mitosis, or whose function has not yet been elucidated, but whose corresponding phosphoproteins have been shown to have important roles in the onset of mitosis and mitotic progression. Examples of the most interesting CDK1-phosphorylated sites and their functions are given.

In our study, seven phosphorylation sites were detected on **stathmin (STMN1)**; three of them were upregulated (Ser 16, Ser 25, and Ser 63). Multiple kinases are known or predicted to phosphorylate the upregulated sites; CDK1 dysregulation might be responsible for elevated Ser 25 phosphorylation. These STMN1 sites have been shown to be phosphorylated in a cell cycle specific manner and to be essential for G2/M transition and proper spindle formation [1]. Nevertheless, increased Ser 16 and Ser 63 phosphorylation have been also observed after apoptosis induction [2].

Protein regulator of cytokinesis 1 (PRC1) is a key regulator of cytokinesis shown to be phosphorylated by CDK1 in early mitosis. We detected increased phosphorylation of "regulatory" site Thr 481, previously described as regulating PRC1 interaction with another mitotic kinase, Polo-like kinase 1 (PLK1) [3].

Thr 926 phosphorylation of **kinesin-like protein KIF11 (KIF11)** is a mitosis-specific phosphorylation conducted by CDK1 which regulates KIF11 interaction with the mitotic spindle, and thus regulates mitosis [4].

Another example of an elevated mitosis-specific phosphorylation detected in our study is Ser 1213 phosphorylation of **DNA topoisomerase 2-alpha (TOP2A).** Ser 1213 has been shown to be phosphorylated by proline-directed kinases (CDK1 or ERKs) inducing the localization of TOP2A to mitotic chromosomes [5].

Sororin (CDCA5) is CDK1-phosphorylated in mitosis (Ser 75 and Ser 79), and this modification causes its release from chromatin while affecting sister chromatid cohesion [6].

Protein phosphorylation of the N-terminal domain of **nucleolin (NCL)** has been shown to be essential for normal proliferation [7]; Thr 121 detected in our study has been already detected as a substrate of CDK1 in a high-throughput phosphoproteomic screen for CDK1 substrates [8].

Nuclear ubiquitous casein and cyclin-dependent kinase substrate 1 (NUCKS1) – Ser 181 phosphorylation – is another example of a CDK1 substrate identified in a high-throughput experiment [8]. Furthermore, Bensimon et al. showed that phosphorylation of NUCKS1 Ser 181 after neocarzinostatin (NCS) treatment was ATM-dependent [9], and it has been shown recently that NUCKS1 plays a role in homology directed repair of damaged DNA [10].

Lamina-associated polypeptide 2, isoform alpha (TMPO) has been shown to be phosphorylated in a mitosis-specific manner and might be important for post-mitotic nuclear assembly [11]. However, Ser 424 upregulated in our study does not have any known function.

Components of nuclear lamina are known to be hyper-phosphorylated to induce reversible disintegration of the nuclear envelope. We detected four upregulated

phosphorylation sites of nuclear lamins induced by VE-821 pre-treatment of irradiated cells: **lamin-B1 (LMNB1;** Ser 23, Thr 20, and Thr 5) and **lamin-B2 (LMNB2;** Ser 37).

Overview of Aurora A and B substrates significantly affected by VE-821 treatment in irradiated MOLT-4 cells.

Kinases from the Aurora kinase family - Aurora kinases A and B - are master regulators of mitosis progression and onset of cytokinesis, whose activities require tight spatial and temporal regulation, and their dysregulation could cause errors in mitosis, faster progression through the abscission checkpoint, and affect postmitotic genome surveillance. In our analysis, we found that the activities of these two kinases might be upregulated by VE-821 treatment. As in the case of CDKs, examples of the most interesting phosphorylation sites are given.

Targeting protein for Xklp2 (TPX2) is a spindle assembly factor, whose activity is tightly interconnected with Aurora kinase A activity. TPX2 contributes to Aurora A activation [12], while on the other hand Aurora A has been shown to phosphorylate TPX2 on Ser 121 and Ser 125 to regulate mitotic spindle length and control microtubule flux [13]. In addition to these two known “regulatory” sites we detected three more upregulated phosphorylation sites on this protein that were predicted to be possible substrates of Aurora A (Ser 486, Thr 369, and Ser 738).

Kinesin-like protein KIF2C (KIF2C) Ser 115 has previously been shown to be phosphorylated by Aurora B, and this phosphorylation regulates KIF2C interaction with centromeres and kinetochores and its microtubule depolymerisation activity. Thus, it regulates the turnover of microtubules at the kinetochore and chromosome segregation during mitosis [14].

Another known Aurora B substrate found upregulated in our study was **chromobox protein homolog 5 (CBX5)** that has been previously shown to be hyper-phosphorylated during mitosis [15].

Notably, we found hyper-phosphorylation of **antigen KI-67 (MKI67)** (from 22 sites detected, 12 were upregulated in GRT), with most of the sites predicted to be substrates of Aurora A, CDK1, or NEK kinases by Networkin 3. MKI67 is a protein essential for normal cellular proliferation [16] and has been shown to interact with known mitotic proteins [17].

Taken together, our data indicate that ATR inhibition induced dysregulation of the main mitotic kinases. Data mining in published studies confirmed that many of the substrates assigned to each one of these mitotic kinases have already been described as the essential players in cell cycle and mitosis control.

Overview of mTOR downstream substrates significantly affected by VE-821 treatment in irradiated MOLT-4 cells.

The protein kinase **mTOR** is the principle regulator of cellular metabolism promoting anabolic processes and inhibiting catabolic processes such as autophagy. It integrates signals from different upstream pathways triggered by a wide variety of agents including nutrients, hormones, growth factors, and also cellular stresses to regulate cell growth, metabolism, cell survival, protein synthesis, and transcription (reviewed in [18]). In total, we found seven known direct mTOR targets downregulated in our study and several other proteins included in the mTOR signalling pathway from the KEGG pathway database.

Four of the mTOR-phosphorylated proteins are involved in transcription and translation regulation and thus contribute to regulation of cell growth and proliferation. **Eukaryotic translation initiation factor 4E-binding protein 1 (EIF4EBP1)** Ser 65 is a known “regulatory” site targeted by mTOR or MAPK-driven pathways. Together with phosphorylation of Thr 70 and Ser 65, phosphorylation affects translation initiation – when hypo-phosphorylated, EIF4EBP1 strongly binds to EIF4E, an essential member of the translation initiation EIF4 complex [19].

Furthermore, we found four downregulated phosphorylation sites on **protein PAT1 homolog 1 (PATL1;** Ser 179 and Ser 184) and **la-related protein 1 (LARP1;** Ser 766 and Ser 774); both proteins have been described as downstream mTOR targets involved in regulating mRNA stability and degradation [20,21]. Both of them were also identified as rapamycin sensitive phosphorylation sites in a high throughput phosphoproteomic screening for potential mTOR substrates [22].

Moreover, we also detected downregulation of an mTOR mediated phosphorylation of **repressor of RNA polymerase III transcription MAF1 homolog (MAF1),** a previously described regulator of transcription; Ser 75 phosphorylation has been described as a “regulatory” protein modification decreasing RNA polymerase III (Pol III) transcription repressing the activity of MAF1 [23].

In addition to proteins involved in translation regulation, we also identified significantly changed phosphorylation of two phosphoproteins linked to autophagy, an evolutionarily conserved process that enables degradation and recycling of proteins or whole organelles to maintain cellular homeostasis under both normal and stress conditions (reviewed in [24]). **Death-associated protein 1 (DAP1)** is a suppressor of autophagy in growing cells that is functionally silenced through mTOR-dependent phosphorylations on Ser 3 (downregulated in our study) and Ser 51. Inactivation of mTOR has been shown to rapidly reduce phosphorylation of these sites and to activate the repressor function of DAP1 [25].

Serine/threonine-protein kinase ULK1 (ULK1) is a protein kinase that plays a key role in starvation-induced autophagy. Under normal growth conditions, ULK1 is phosphorylated and negatively regulated by mTOR. Serine 450, detected in our study and predicted to be a substrate of mTOR by the iGPS prediction algorithm, has been shown to be dephosphorylated upon starvation [26]. Unfortunately, we did not detect Ser 638 and Ser 758

that have been further assigned as functional phosphorylation sites suppressing autophagy induction by ULK1 under normal cell growth conditions [26].

1. Larsson N, Marklund U, Gradin HM, Brattsand G, Gullberg M. Control of microtubule dynamics by oncoprotein 18: dissection of the regulatory role of multisite phosphorylation during mitosis. Mol Cell Biol. 1997;17: 5530–5539.

2. Vancompernolle K, Boonefaes T, Mann M, Fiers W, Grooten J. Tumor Necrosis Factor-induced Microtubule Stabilization Mediated by Hyperphosphorylated Oncoprotein 18 Promotes Cell Death. J Biol Chem. 2000;275: 33876–33882. doi:10.1074/jbc.M004785200

3. Abe Y, Takeuchi T, Kagawa-Miki L, Ueda N, Shigemoto K, Yasukawa M, et al. A Mitotic Kinase TOPK Enhances Cdk1/cyclin B1-dependent Phosphorylation of PRC1 and Promotes Cytokinesis. Journal of Molecular Biology. 2007;370: 231–245. doi:10.1016/j.jmb.2007.04.067

4. Slangy A, Lane HA, d'Hérin P, Harper M, Kress M, Niggt EA. Phosphorylation by p34cdc2 regulates spindle association of human Eg5, a kinesin-related motor essential for bipolar spindle formation in vivo. Cell. 1995;83: 1159–1169. doi:10.1016/0092-8674(95)90142-6

5. Ishida R, Takashima R, Koujin T, Shibata M, Nozaki N, Seto M, et al. Mitotic Specific Phosphorylation of Serine-1212 in Human DNA Topoisomerase IIα. Cell Structure and Function. 2001;26: 215–226. doi:10.1247/csf.26.215

6. Dreier MR, Bekier ME, Taylor WR. Regulation of sororin by Cdk1-mediated phosphorylation. J Cell Sci. 2011;124: 2976–2987. doi:10.1242/jcs.085431

7. Xiao S, Caglar E, Maldonado P, Das D, Nadeem Z, Chi A, et al. Induced Expression of Nucleolin Phosphorylation-Deficient Mutant Confers Dominant-Negative Effect on Cell Proliferation. PLoS One. 2014;9. doi:10.1371/journal.pone.0109858

8. Blethrow JD, Glavy JS, Morgan DO, Shokat KM. Covalent capture of kinase-specific phosphopeptides reveals Cdk1-cyclin B substrates. Proc Natl Acad Sci U S A. 2008;105: 1442–1447. doi:10.1073/pnas.0708966105

9. Bensimon A, Schmidt A, Ziv Y, Elkon R, Wang S-Y, Chen DJ, et al. ATM-dependent and -independent dynamics of the nuclear phosphoproteome after DNA damage. Sci Signal. 2010;3: rs3. doi:10.1126/scisignal.2001034

10. Parplys AC, Zhao W, Sharma N, Groesser T, Liang F, Maranon DG, et al. NUCKS1 is a novel RAD51AP1 paralog important for homologous recombination and genome stability. Nucleic Acids Res. 2015;43: 9817–9834. doi:10.1093/nar/gkv859

11. Dechat T, Gotzmann J, Stockinger A, Harris CA, Talle MA, Siekierka JJ, et al. Detergent-salt resistance of LAP2alpha in interphase nuclei and phosphorylation-dependent association with chromosomes early in nuclear assembly implies functions in nuclear structure dynamics. EMBO J. 1998;17: 4887–4902. doi:10.1093/emboj/17.16.4887

12. Bird AW, Hyman AA. Building a spindle of the correct length in human cells requires the interaction between TPX2 and Aurora A. J Cell Biol. 2008;182: 289–300. doi:10.1083/jcb.200802005

13. Fu J, Bian M, Xin G, Deng Z, Luo J, Guo X, et al. TPX2 phosphorylation maintains metaphase spindle length by regulating microtubule flux. J Cell Biol. 2015;210: 373–383. doi:10.1083/jcb.201412109

14. Andrews PD, Ovechkina Y, Morrice N, Wagenbach M, Duncan K, Wordeman L, et al. Aurora B Regulates MCAK at the Mitotic Centromere. Developmental Cell. 2004;6: 253–268. doi:10.1016/S1534-5807(04)00025-5

15. Minc E, Allory Y, Worman HJ, Courvalin JC, Buendia B. Localization and phosphorylation of HP1 proteins during the cell cycle in mammalian cells. Chromosoma. 1999;108: 220–234.

16. Schlütter C, Duchrow M, Wohlenberg C, Becker M, Key G, Flad H, et al. The cell proliferation-associated antigen of antibody Ki-67: a very large, ubiquitous nuclear protein with numerous repeated elements, representing a new kind of cell cycle-maintaining proteins. J Cell Biol. 1993;123: 513–522.

17. Sueishi M, Takagi M, Yoneda Y. The Forkhead-associated Domain of Ki-67 Antigen Interacts with the Novel Kinesin-like Protein Hklp2. J Biol Chem. 2000;275: 28888–28892. doi:10.1074/jbc.M003879200

18. Shimobayashi M, Hall MN. Making new contacts: the mTOR network in metabolism and signalling crosstalk. Nat Rev Mol Cell Biol. 2014;15: 155–162. doi:10.1038/nrm3757

19. Lekmine F, Sassano A, Uddin S, Smith J, Majchrzak B, Brachmann SM, et al. Interferon-γ engages the p70 S6 kinase to regulate phosphorylation of the 40S S6 ribosomal protein. Experimental Cell Research. 2004;295: 173–182. doi:10.1016/j.yexcr.2003.12.021

20. Ozgur S, Chekulaeva M, Stoecklin G. Human Pat1b Connects Deadenylation with mRNA Decapping and Controls the Assembly of Processing Bodies. Mol Cell Biol. 2010;30: 4308–4323. doi:10.1128/MCB.00429-10

21. Tcherkezian J, Cargnello M, Romeo Y, Huttlin EL, Lavoie G, Gygi SP, et al. Proteomic analysis of cap-dependent translation identifies LARP1 as a key regulator of 5′TOP mRNA translation. Genes Dev. 2014;28: 357–371. doi:10.1101/gad.231407.113

22. Hsu PP, Kang SA, Rameseder J, Zhang Y, Ottina KA, Lim D, et al. The mTOR-regulated phosphoproteome reveals a mechanism of mTORC1-mediated inhibition of growth factor signaling. Science. 2011;332: 1317–1322. doi:10.1126/science.1199498

23. Shor B, Wu J, Shakey Q, Toral-Barza L, Shi C, Follettie M, et al. Requirement of the mTOR Kinase for the Regulation of Maf1 Phosphorylation and Control of RNA Polymerase III-dependent Transcription in Cancer Cells. J Biol Chem. 2010;285: 15380–15392. doi:10.1074/jbc.M109.071639

24. Ondrej M, Cechakova L, Durisova K, Pejchal J, Tichy A. To live or let die: Unclear task of autophagy in the radiosensitization battle. Radiotherapy and Oncology. 2016; doi:10.1016/j.radonc.2016.02.028

25. Koren I, Reem E, Kimchi A. DAP1, a Novel Substrate of mTOR, Negatively Regulates Autophagy. Current Biology. 2010;20: 1093–1098. doi:10.1016/j.cub.2010.04.041

26. Shang L, Chen S, Du F, Li S, Zhao L, Wang X. Nutrient starvation elicits an acute autophagic response mediated by Ulk1 dephosphorylation and its subsequent dissociation from AMPK. Proc Natl Acad Sci U S A. 2011;108: 4788–4793. doi:10.1073/pnas.1100844108
