# Supplementary material for: Radio-sensitizing effects of VE-821 and beyond: Distinct phosphoproteomic and metabolomic changes after ATR inhibition in irradiated MOLT-4 cells
Source: PLoS One. 2018 Jul 12;13(7):e0199349. doi: 10.1371/journal.pone.0199349 (PMC6042708; doi:10.1371/journal.pone.0199349)
Supplement: S2 File — Metabolite changes were visualized in KEGG pathway maps. Purple and yellow circles represent metabolites upregulated and downregulated, respectively, in response to VE-821 treatment. Left and right halves of the metabolites show changes 6 and 12 hours after irradiation, respectively. (PDF) [file pone.0199349.s002.pdf]

S5 KEGG pathway maps visualization. Metabolite changes were visualized in KEGG pathway maps. Purple and yellow circles represent metabolites upregulated and downregulated, respectively, in response to VE-821 treatment. Left and right halves of the metabolites show changes 6 and 12 hours after irradiation, respectively.

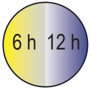

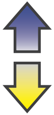

metabolites upregulated in VE-821 treated cells

metabolites downregulated in VE-821 treated cells

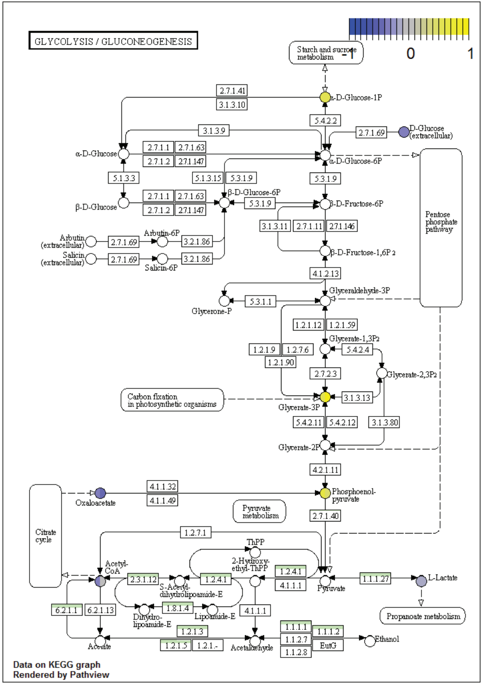

GLYCOLYSIS / GLUCONEOGENESIS
-1
0
1
Starch and sucrose metabolism
α-D-Glucose-1P
2.7.1.41
3.1.3.10
5.4.2.2
3.1.3.9
2.7.1.69
D-Glucose (extracellular)
α-D-Glucose
2.7.1.1
2.7.1.63
2.7.1.2
2.7.1.147
α-D-Glucose-6P
5.1.3.3
5.1.3.15
5.3.1.9
β-D-Glucose
β-D-Glucose-6P
β-D-Fructose-6P
Pentose phosphate pathway
3.1.3.11
2.7.1.11
2.7.1.146
Arbutin (extracellular)
Arbutin-6P
3.2.1.86
Salicin (extracellular)
Salicin-6P
β-D-Fructose-1,6P2
4.1.2.13
Glyceraldehyde-3P
5.3.1.1
Glycerone-P
1.2.1.12
1.2.1.59
Glycerate-1,3P2
1.2.1.9
1.2.7.6
5.4.2.4
1.2.1.90
2.7.2.3
Glycerate-2,3P2
Carbon fixation in photosynthetic organisms
3.1.3.13
Glycerate-3P
5.4.2.11
5.4.2.12
3.1.3.80
Glycerate-2P
4.2.1.11
4.1.1.32
4.1.1.49
Oxaloacetate
Phosphoenol-pyruvate
Citrate cycle
Pyruvate metabolism
2.7.1.40
1.2.7.1
ThPP
2-Hydroxy-ethyl-ThPP
Acetyl-CoA
2.3.1.12
S-Acetyl-dihydrolipoamide-E
1.2.4.1
4.1.1.1
Pyruvate
1.1.1.27
L-Lactate
Propanoate metabolism
6.2.1.1
6.2.1.13
Dihydro-lipoamide-E
1.8.1.4
Lipoamide-E
1.2.1.3
1.2.1.5
1.2.1.-
Acetate
Acetaldehyde
1.1.1.1
1.1.1.2
1.1.2.7
EutG
1.1.2.8
Ethanol
Data on KEGG graph
Rendered by Pathview

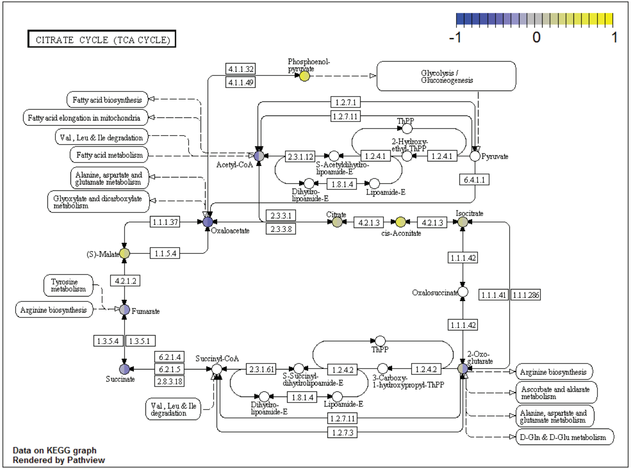
CITRATE CYCLE (TCA CYCLE)
-1
0
1
Phosphoenol-pyruvate
4.1.1.32
4.1.1.49
Glycolysis / Gluconeogenesis
Fatty acid biosynthesis
Fatty acid elongation in mitochondria
Val, Leu & Ile degradation
Fatty acid metabolism
Alanine, aspartate and glutamate metabolism
Glyoxylate and dicarboxylate metabolism
1.2.7.1
1.2.7.11
ThPP
2-Hydroxy-ethyl-ThPP
Acetyl-CoA
2.3.1.12
S-Acetyldihydro-lipoamide-E
1.2.4.1
1.2.4.1
Pyruvate
1.8.1.4
Dihydro-lipoamide-E
Lipoamide-E
6.4.1.1
2.3.3.1
2.3.3.8
Citrate
4.2.1.3
cis-Aconitate
4.2.1.3
Isocitrate
1.1.1.37
Oxaloacetate
(S)-Malate
1.1.5.4
4.2.1.2
Tyrosine metabolism
Arginine biosynthesis
Fumarate
1.1.1.42
Oxalosuccinate
1.1.1.41
1.1.1.286
1.1.1.42
1.3.5.4
1.3.5.1
6.2.1.4
6.2.1.5
2.8.3.18
Succinate
Succinyl-CoA
2.3.1.61
S-Succinyl-dihydrolipoamide-E
1.2.4.2
3-Carboxy-1-hydroxypropyl-ThPP
1.2.4.2
2-Oxo-glutarate
Arginine biosynthesis
Ascorbate and aldarate metabolism
Alanine, aspartate and glutamate metabolism
D-Gln & D-Glu metabolism
Val, Leu & Ile degradation
1.8.1.4
Dihydro-lipoamide-E
Lipoamide-E
1.2.7.11
1.2.7.3
Data on KEGG graph
Rendered by Pathview

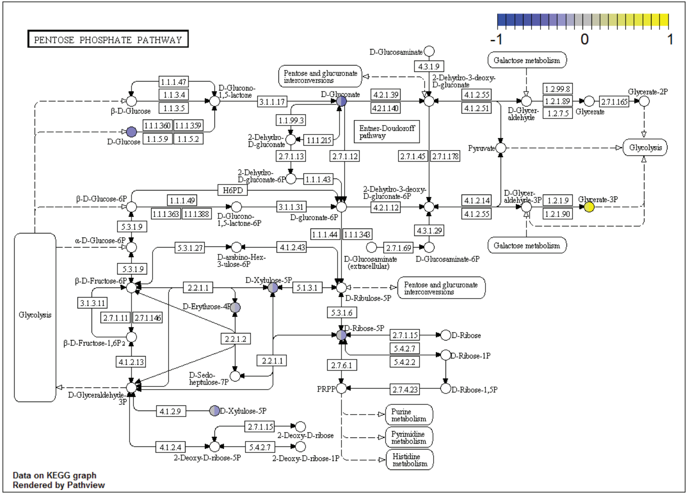

PENTOSE PHOSPHATE PATHWAY
-1
0
1
D-Glucosamine
4.3.1.9
Galactose metabolism
Pentose and glucuronate interconversions
2-Dehydro-3-deoxy-D-gluconate
1.1.1.47
D-Glucono-1,5-lactone
1.1.3.4
1.1.3.5
3.1.1.17
D-Gluconate
4.2.1.39
4.2.1.140
4.1.2.55
4.1.2.51
1.2.99.8
1.2.1.89
1.2.7.5
2.7.1.165
Glycerate-2P
Glycerate
D-Glyceraldehyde
β-D-Glucose
1.1.99.3
1.1.1.360
1.1.1.359
1.1.5.9
1.1.5.2
D-Glucose
2-Dehydro-D-gluconate
1.1.1.215
Entner-Doudoroff pathway
Pyruvate
Glycolysis
2.7.1.13
2.7.1.12
2.7.1.45
2.7.1.178
2-Dehydro-D-gluconate-6P
1.1.1.43
H6PD
2-Dehydro-3-deoxy-D-gluconate-6P
D-Glyceraldehyde-3P
4.1.2.14
4.1.2.55
1.2.1.9
1.2.1.90
Glycerate-3P
β-D-Glucose-6P
1.1.1.49
1.1.1.363
1.1.1.388
D-Glucono-1,5-lactone-6P
3.1.1.31
D-gluconate-6P
4.2.1.12
5.3.1.9
1.1.1.44
1.1.1.343
4.3.1.29
α-D-Glucose-6P
5.3.1.27
4.1.2.43
2.7.1.69
D-arabino-Hex-3-ulose-6P
D-Glucosamine (extracellular)
D-Glucosamine-6P
5.3.1.9
β-D-Fructose-6P
2.2.1.1
D-Xylulose-5P
5.1.3.1
D-Ribulose-5P
3.1.3.11
D-Erythrose-4P
Glycolysis
2.7.1.11
2.7.1.146
5.3.1.6
D-Ribose-5P
2.7.1.15
D-Ribose
β-D-Fructose-1,6P2
2.2.1.2
5.4.2.7
D-Ribose-1P
5.4.2.2
4.1.2.13
2.2.1.1
2.7.6.1
D-Sedo-heptulose-7P
D-Glyceraldehyde-3P
PRPP
2.7.4.23
D-Ribose-1,5P
4.1.2.9
D-Xylulose-5P
Purine metabolism
2.7.1.15
2-Deoxy-D-ribose
Pyrimidine metabolism
4.1.2.4
5.4.2.7
2-Deoxy-D-ribose-5P
2-Deoxy-D-ribose-1P
Histidine metabolism
Data on KEGG graph
Rendered by Pathview

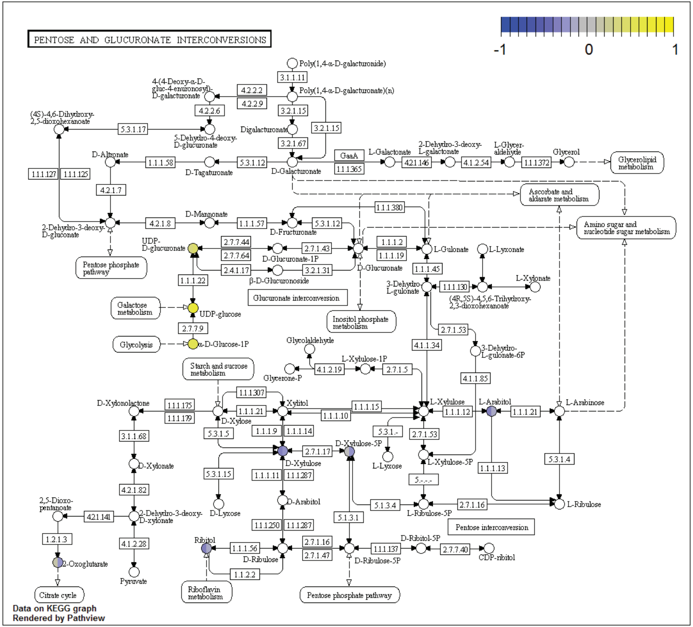
PENTOSE AND GLUCURONATE INTERCONVERSIONS
-1
0
1
Poly(1,4-α-D-galacturonide)
3.1.1.11
Poly(1,4-α-D-galacturonate)(n)
4-(4-Deoxy-α-D-gluc-4-enuronosyl)-D-galacturonate
4.2.2.2
4.2.2.9
4.2.2.6
3.2.1.15
3.2.1.15
Digalacturonate
3.2.1.67
(4S)-4,6-Dihydroxy-2,5-dioxohexanoate
5.3.1.17
5-Dehydro-4-deoxy-D-glucuronate
D-Altronate
1.1.1.58
D-Tagaturonate
5.3.1.12
D-Galacturonate
GaaA
1.1.1.365
L-Galactonate
4.2.1.146
2-Dehydro-3-deoxy-L-galactonate
4.1.2.54
L-Glyceraldehyde
1.1.1.372
Glycerol
Glycerolipid metabolism
1.1.1.127
1.1.1.125
4.2.1.7
Ascorbate and aldarate metabolism
1.1.1.380
D-Mannonate
2-Dehydro-3-deoxy-D-gluconate
4.2.1.8
1.1.1.57
D-Fructuronate
5.3.1.12
Amino sugar and nucleotide sugar metabolism
UDP-D-glucuronate
2.7.7.44
2.7.7.64
D-Glucuronate-1P
2.7.1.43
1.1.1.2
1.1.1.19
L-Gulonate
L-Lyxonate
Pentose phosphate pathway
2.4.1.17
3.2.1.31
β-D-Glucuronoside
D-Glucuronate
1.1.1.45
1.1.1.22
L-Xylonate
3-Dehydro-L-gulonate
1.1.1.130
(4R,5S)-4,5,6-Trihydroxy-2,3-dioxohexanoate
Glucuronate interconversion
Galactose metabolism
UDP-glucose
Inositol phosphate metabolism
2.7.7.9
2.7.1.53
Glycolysis
α-D-Glucose-1P
Glycolaldehyde
4.1.1.34
3-Dehydro-L-gulonate-6P
L-Xylulose-1P
Starch and sucrose metabolism
4.1.2.19
2.7.1.5
Glycerone-P
4.1.1.85
1.1.1.307
D-Xylonolactone
1.1.1.175
1.1.1.179
Xylitol
1.1.1.15
L-Xylulose
L-Arabitol
L-Arabinose
1.1.1.21
1.1.1.10
1.1.1.12
1.1.1.21
D-Xylose
3.1.1.68
5.3.1.5
1.1.1.9
1.1.1.14
5.3.1.-
2.7.1.53
D-Xylulose-5P
2.7.1.17
D-Xylonate
D-Xylulose
L-Lyxose
L-Xylulose-5P
5.3.1.15
1.1.1.11
1.1.1.287
5.-.-.-
1.1.1.13
5.3.1.4
4.2.1.82
D-Arabitol
D-Lyxose
5.1.3.4
2.7.1.16
L-Ribulose
2,5-Dioxopentanoate
4.2.1.141
2-Dehydro-3-deoxy-D-xylonate
L-Ribulose-5P
1.1.1.250
1.1.1.287
5.1.3.1
Pentose interconversion
1.2.1.3
4.1.2.28
Ribitol
1.1.1.56
2.7.1.16
2.7.1.47
D-Ribulose
1.1.1.137
D-Ribitol-5P
2.7.7.40
CDP-ribitol
2-Oxoglutarate
D-Ribulose-5P
Pyruvate
1.1.2.2
Riboflavin metabolism
Citrate cycle
Pentose phosphate pathway
Data on KEGG graph
Rendered by Pathview

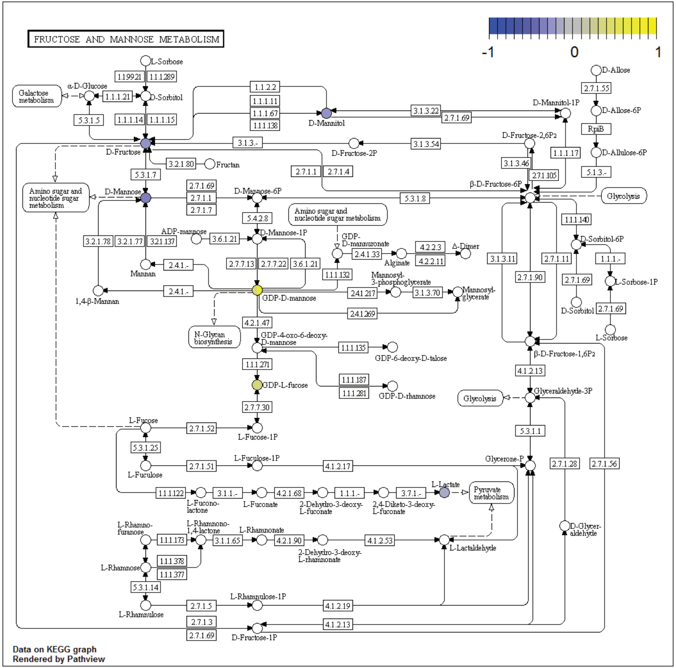
FRUCTOSE AND MANNOSE METABOLISM
-1
0
1
L-Sorbose
1.1.99.21
1.1.1.289
Galactose metabolism
α-D-Glucose
1.1.1.21
D-Sorbitol
5.3.1.5
1.1.1.14
1.1.1.15
1.1.2.2
1.1.1.11
1.1.1.67
1.1.1.138
D-Mannitol
3.1.3.22
2.7.1.69
D-Mannitol-1P
D-Allose
2.7.1.55
D-Allose-6P
RpiB
D-Allulose-6P
5.1.3.-
3.1.3.-
D-Fructose
D-Fructose-2P
3.1.3.54
D-Fructose-2,6P2
3.1.3.46
2.7.1.105
1.1.1.17
3.2.1.80
Fructan
2.7.1.1
2.7.1.4
5.3.1.7
β-D-Fructose-6P
Glycolysis
Amino sugar and nucleotide sugar metabolism
D-Mannose
2.7.1.69
2.7.1.1
2.7.1.7
D-Mannose-6P
5.3.1.8
1.1.1.140
5.4.2.8
D-Mannose-1P
ADP-mannose
3.6.1.21
3.2.1.78
3.2.1.77
3.2.1.137
GDP-D-mannuronate
2.4.1.33
Alginate
4.2.2.3
4.2.2.11
Δ-Dimer
D-Sorbitol-6P
3.1.3.11
2.7.1.11
1.1.1.-
Mannan
2.4.1.-
2.7.7.13
2.7.7.22
3.6.1.21
1.1.1.132
Mannosyl-3-phosphoglycerate
2.7.1.90
2.7.1.69
L-Sorbose-1P
1,4-β-Mannan
2.4.1.-
GDP-D-mannose
2.4.1.217
3.1.3.70
Mannosyl-glycerate
D-Sorbitol
2.7.1.69
2.4.1.269
4.2.1.47
L-Sorbose
N-Glycan biosynthesis
GDP-4-oxo-6-deoxy-D-mannose
1.1.1.135
GDP-6-deoxy-D-talose
β-D-Fructose-1,6P2
1.1.1.271
4.1.2.13
1.1.1.187
1.1.1.281
GDP-L-fucose
GDP-D-rhamnose
Glycolysis
Glyceraldehyde-3P
2.7.7.30
5.3.1.1
L-Fucose
2.7.1.52
L-Fucose-1P
5.3.1.25
L-Fuculose-1P
Glycerone-P
2.7.1.28
2.7.1.56
L-Fuculose
2.7.1.51
4.1.2.17
L-Lactate
1.1.1.122
3.1.1.-
4.2.1.68
1.1.1.-
3.7.1.-
Pyruvate metabolism
L-Fucono-lactone
L-Fuconate
2-Dehydro-3-deoxy-L-fuconate
2,4-Diketo-3-deoxy-L-fuconate
L-Rhamno-furanose
L-Rhamnono-1,4-lactone
L-Rhamnonate
D-Glyceraldehyde
1.1.1.173
3.1.1.65
4.2.1.90
4.1.2.53
2-Dehydro-3-deoxy-L-rhamnonate
L-Lactaldehyde
1.1.1.378
L-Rhamnose
1.1.1.377
5.3.1.14
L-Rhamnulose-1P
2.7.1.5
4.1.2.19
L-Rhamnulose
2.7.1.3
4.1.2.13
2.7.1.69
D-Fructose-1P
Data on KEGG graph
Rendered by Pathview

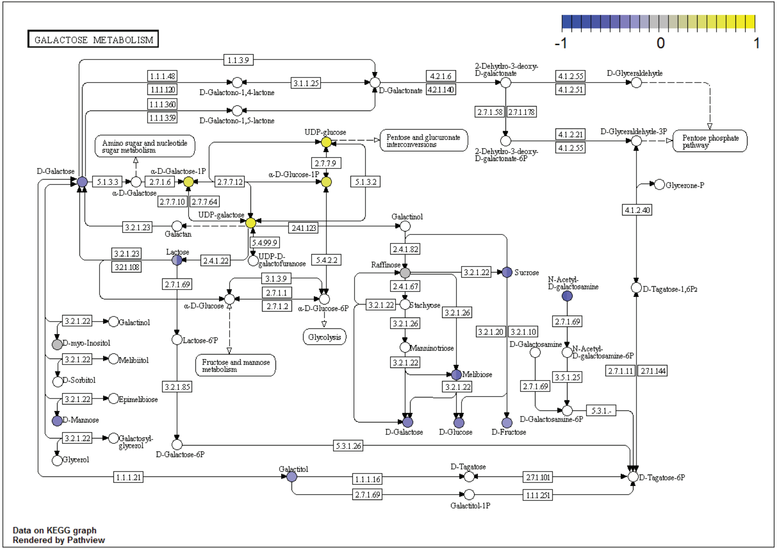
GALACTOSE METABOLISM
-1
0
1
D-Galactose
α-D-Galactose
α-D-Galactose-1P
UDP-glucose
α-D-Glucose-1P
UDP-galactose
Galactan
Lactose
UDP-D-galactofuranose
α-D-Glucose
α-D-Glucose-6P
Lactose-6P
D-Galactose-6P
D-Galactono-1,4-lactone
D-Galactono-1,5-lactone
D-Galactonate
2-Dehydro-3-deoxy-D-galactonate
2-Dehydro-3-deoxy-D-galactonate-6P
D-Glyceraldehyde
D-Glyceraldehyde-3P
Glycerone-P
D-Tagatose-1,6P2
Amino sugar and nucleotide sugar metabolism
Pentose and glucuronate interconversions
Pentose phosphate pathway
Glycolysis
Fructose and mannose metabolism
Galactinol
Raffinose
Sucrose
Stachyose
Manninotriose
Melibiose
D-Galactose
D-Glucose
D-Fructose
N-Acetyl-D-galactosamine
N-Acetyl-D-galactosamine-6P
D-Galactosamine
D-Galactosamine-6P
D-myo-Inositol
Melibiitol
D-Sorbitol
Epimelibiose
D-Mannose
Galactosyl-glycerol
Glycerol
Galactitol
D-Tagatose
Galactitol-1P
D-Tagatose-6P
1.1.3.9
1.1.1.48
1.1.1.120
1.1.1.360
1.1.1.359
3.1.1.25
4.2.1.6
4.2.1.140
4.1.2.55
4.1.2.51
2.7.1.58
2.7.1.178
4.1.2.21
4.1.2.55
5.1.3.3
2.7.1.6
2.7.7.10
2.7.7.64
2.7.7.12
2.7.7.9
5.1.3.2
3.2.1.23
2.4.1.123
5.4.99.9
3.2.1.23
3.2.1.108
2.4.1.22
5.4.2.2
2.7.1.69
3.1.3.9
2.7.1.1
2.7.1.2
4.1.2.40
2.4.1.82
3.2.1.22
2.4.1.67
3.2.1.22
3.2.1.26
3.2.1.26
3.2.1.22
3.2.1.22
3.2.1.20
3.2.1.10
2.7.1.69
3.5.1.25
2.7.1.69
5.3.1.-
2.7.1.11
2.7.1.144
3.2.1.22
3.2.1.22
3.2.1.22
3.2.1.22
3.2.1.85
5.3.1.26
1.1.1.21
1.1.1.16
2.7.1.101
2.7.1.69
1.1.1.251
Data on KEGG graph
Rendered by Pathview

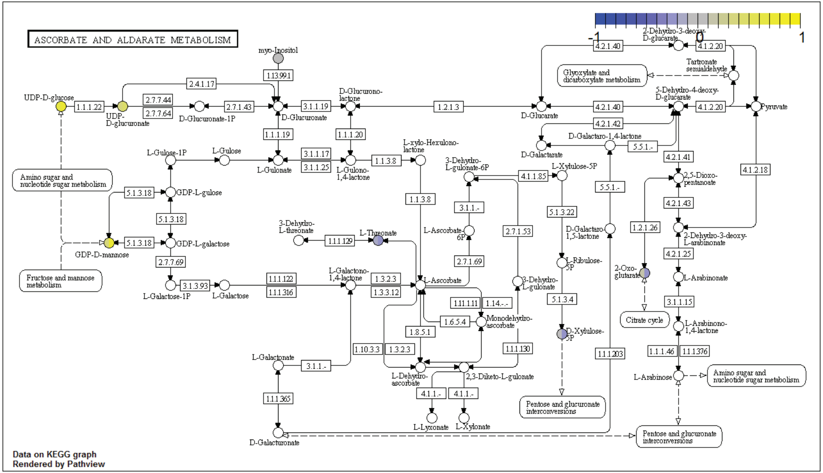

ASCORBATE AND ALDARATE METABOLISM
-1
0
1
myo-Inositol
1.13.99.1
2.4.1.17
UDP-D-glucose
1.1.1.22
UDP-D-glucuronate
2.7.7.44
2.7.7.64
D-Glucuronate-1P
2.7.1.43
D-Glucuronate
3.1.1.19
D-Glucurono-lactone
1.2.1.3
D-Glucarate
4.2.1.40
2-Dehydro-3-deoxy-D-glucarate
4.1.2.20
Tartronate semialdehyde
Glyoxylate and dicarboxylate metabolism
5-Dehydro-4-deoxy-D-glucarate
Pyruvate
4.2.1.42
D-Galactaro-1,4-lactone
5.5.1.-
D-Galactarate
4.2.1.41
4.1.2.18
2,5-Dioxo-pentanoate
4.2.1.43
1.2.1.26
2-Dehydro-3-deoxy-L-arabinonate
4.2.1.25
L-Arabinonate
3.1.1.15
L-Arabinono-1,4-lactone
1.1.1.46
1.1.1.376
L-Arabinose
Amino sugar and nucleotide sugar metabolism
Pentose and glucuronate interconversions
2-Oxo-glutarate
Citrate cycle
1.1.1.19
1.1.1.20
L-Gulose-1P
L-Gulose
L-Gulonate
3.1.1.17
3.1.1.25
L-Gulono-1,4-lactone
1.1.3.8
L-xylo-Hexulono-lactone
3-Dehydro-L-gulonate-6P
4.1.1.85
L-Xylulose-5P
5.1.3.22
L-Ribulose-5P
5.1.3.4
D-Xylulose-5P
3.1.1.-
L-Ascorbate-6P
2.7.1.69
2.7.1.53
3-Dehydro-L-gulonate
1.1.1.130
D-Galactaro-1,5-lactone
1.1.1.203
Amino sugar and nucleotide sugar metabolism
5.1.3.18
GDP-L-gulose
GDP-L-galactose
GDP-D-mannose
2.7.7.69
Fructose and mannose metabolism
L-Galactose-1P
3.1.3.93
L-Galactose
1.1.1.122
1.1.1.316
L-Galactono-1,4-lactone
1.3.2.3
1.3.3.12
L-Ascorbate
3-Dehydro-L-threonate
1.1.1.129
L-Threonate
1.1.1.11
1.14.-.-
1.6.5.4
Monodehydro-ascorbate
1.8.5.1
1.10.3.3
L-Galactonate
3.1.1.-
L-Dehydro-ascorbate
2,3-Diketo-L-gulonate
4.1.1.-
L-Lyxonate
L-Xylonate
1.1.1.365
D-Galacturonate
Pentose and glucuronate interconversions
Data on KEGG graph
Rendered by Pathview

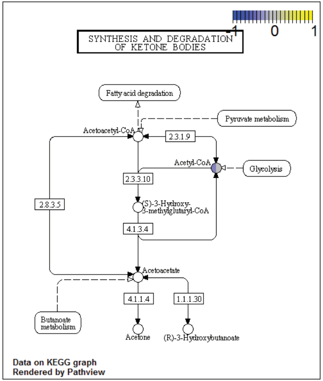

-1
0
1
SYNTHESIS AND DEGRADATION OF KETONE BODIES
Fatty acid degradation
Pyruvate metabolism
Acetoacetyl-CoA
2.3.1.9
Acetyl-CoA
Glycolysis
2.3.3.10
2.8.3.5
(S)-3-Hydroxy-3-methylglutaryl-CoA
4.1.3.4
Acetoacetate
4.1.1.4
1.1.1.30
Butanoate metabolism
Acetone
(R)-3-Hydroxybutanoate
Data on KEGG graph
Rendered by Pathview

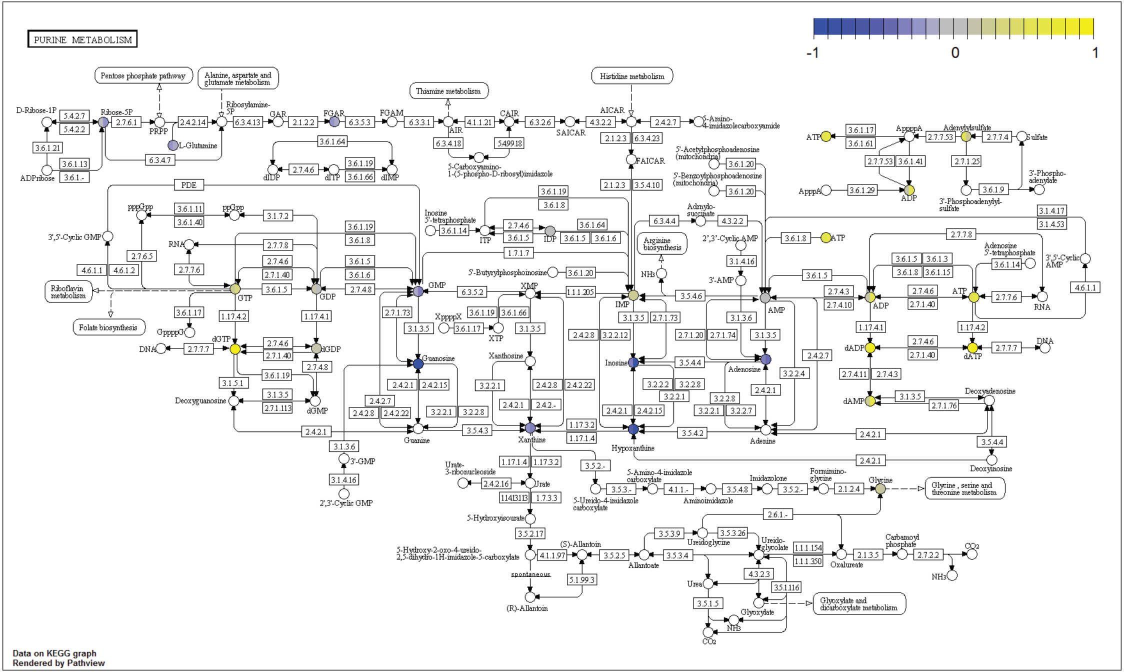
PURINE METABOLISM
-1
0
1
Pentose phosphate pathway
Alanine, aspartate and glutamate metabolism
Thiamine metabolism
Histidine metabolism
D-Ribose-1P
5.4.2.7
5.4.2.2
Ribose-5P
2.7.6.1
PRPP
2.4.2.14
Ribosylamine-5P
6.3.4.13
GAR
2.1.2.2
FGAR
6.3.5.3
FGAM
6.3.3.1
AIR
4.1.1.21
CAIR
6.3.2.6
SAICAR
4.3.2.2
AICAR
2.4.2.7
5-Amino-4-imidazolecarboxyamide
3.6.1.21
3.6.1.13
3.6.1.-
ADPribose
L-Glutamine
6.3.4.7
3.6.1.64
dIDP
2.7.4.6
dITP
3.6.1.19
3.6.1.66
dIMP
6.3.4.18
5.4.99.18
5-Carboxyamino-1-(5-phospho-D-ribosyl)imidazole
2.1.2.3
6.3.4.23
FAICAR
2.1.2.3
3.5.4.10
ATP
3.6.1.17
3.6.1.61
ApppA
2.7.7.53
Adenylylsulfate
2.7.7.4
Sulfate
2.7.7.53
3.6.1.41
2.7.1.25
AppppA
3.6.1.29
ADP
3'-Phosphoadenylyl-sulfate
3.6.1.9
3'-Phospho-adenylate
5'-Acetylphosphoadenosine (mitochondria)
3.6.1.20
5'-Benzoylphosphoadenosine (mitochondria)
3.6.1.20
PDE
ppGpp
3.6.1.11
3.6.1.40
pppGpp
3.1.7.2
3',5'-Cyclic GMP
2.7.6.5
4.6.1.1
4.6.1.2
RNA
2.7.7.8
2.7.7.6
2.7.4.6
2.7.1.40
3.6.1.5
GTP
GDP
3.6.1.19
3.6.1.8
3.6.1.5
3.6.1.6
2.7.4.8
GMP
Riboflavin metabolism
Folate biosynthesis
3.6.1.17
GppppG
1.17.4.2
1.17.4.1
DNA
2.7.7.7
dGTP
2.7.4.6
2.7.1.40
dGDP
2.7.4.8
3.6.1.19
3.1.5.1
3.1.3.5
2.7.1.113
Deoxyguanosine
dGMP
2.4.2.1
Inosine 5'-tetraphosphate
3.6.1.14
ITP
2.7.4.6
3.6.1.5
IDP
3.6.1.19
3.6.1.8
3.6.1.64
3.6.1.5
3.6.1.6
1.7.1.7
5'-Butyrylphosphoinosine
3.6.1.20
6.3.5.2
XMP
1.1.1.205
IMP
2.7.1.73
XppppX
3.6.1.19
3.6.1.66
3.6.1.17
XTP
3.1.3.5
3.1.3.5
Guanosine
Xanthosine
2.4.2.1
2.4.2.15
3.2.2.1
2.4.2.8
2.4.2.22
2.4.2.7
2.4.2.8
2.4.2.22
3.2.2.1
3.2.2.8
2.4.2.1
2.4.2.-
Guanine
3.5.4.3
Xanthine
1.17.3.2
1.17.1.4
3.1.3.6
3'-GMP
3.1.4.16
2',3'-Cyclic GMP
3.1.3.5
2.7.1.73
2.4.2.8
3.2.2.12
Inosine
3.5.4.4
3.2.2.2
3.2.2.8
3.2.2.1
2.4.2.1
2.4.2.15
Hypoxanthine
3.5.4.2
6.3.4.4
Adenylo-succinate
4.3.2.2
Arginine biosynthesis
2',3'-Cyclic AMP
3.1.4.16
NH3
3'-AMP
3.6.1.8
ATP
3.5.4.6
AMP
3.1.3.6
3.1.3.5
2.7.1.20
2.7.1.74
Adenosine
2.4.2.7
3.2.2.4
2.4.2.1
3.2.2.8
3.2.2.1
3.2.2.7
Adenine
3.1.4.17
3.1.4.53
2.7.7.8
3.6.1.5
3.6.1.3
3.6.1.8
3.6.1.15
Adenosine 5'-tetraphosphate
3.6.1.14
3',5'-Cyclic AMP
3.6.1.5
2.7.4.3
2.7.4.10
ADP
2.7.4.6
2.7.1.40
ATP
2.7.7.6
RNA
4.6.1.1
1.17.4.1
1.17.4.2
dADP
2.7.4.6
2.7.1.40
dATP
2.7.7.7
DNA
2.7.4.11
2.7.4.3
dAMP
3.1.3.5
2.7.1.76
Deoxyadenosine
2.4.2.1
3.5.4.4
2.4.2.1
Deoxyinosine
Urate-3-ribonucleoside
1.17.1.4
1.17.3.2
2.4.2.16
Urate
1.14.13.113
1.7.3.3
3.5.2.-
5-Amino-4-imidazole carboxylate
3.5.3.-
4.1.1.-
Imidazolone
3.5.4.8
3.5.2.-
Formimino-glycine
2.1.2.4
Glycine
Glycine , serine and threonine metabolism
5-Ureido-4-imidazole carboxylate
Aminoimidazole
2.6.1.-
5-Hydroxyisourate
3.5.2.17
5-Hydroxy-2-oxo-4-ureido-2,5-dihydro-1H-imidazole-5-carboxylate
4.1.1.97
(S)-Allantoin
3.5.2.5
Allantoate
3.5.3.4
3.5.3.9
Ureidoglycine
3.5.3.26
Ureido-glycolate
1.1.1.154
1.1.1.350
Oxalureate
2.1.3.5
Carbamoyl phosphate
2.7.2.2
CO_2
NH_3
spontaneous
5.1.99.3
(R)-Allantoin
4.3.2.3
Urea
3.5.1.116
Glyoxylate
Glyoxylate and dicarboxylate metabolism
3.5.1.5
NH_3
CO_2
Data on KEGG graph
Rendered by Pathview

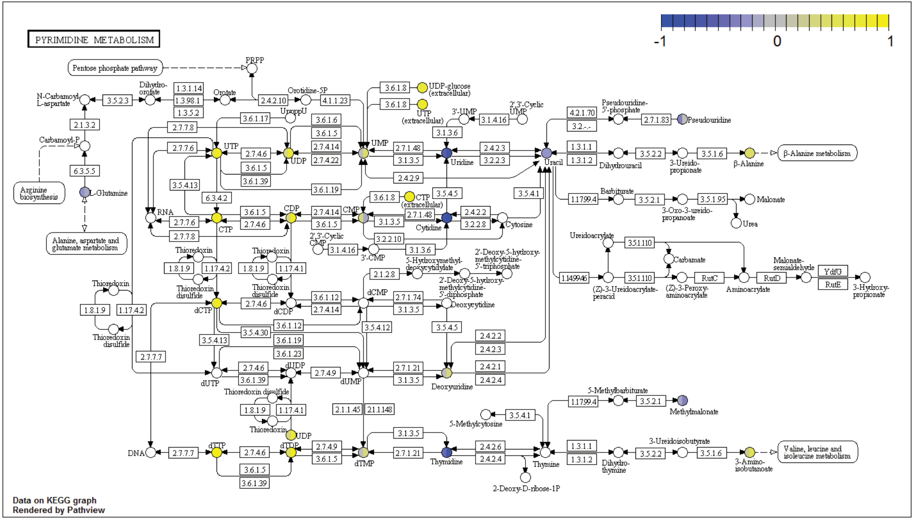
PYRIMIDINE METABOLISM
-1
0
1
Pentose phosphate pathway
PRPP
N-Carbamoyl-L-aspartate
3.5.2.3
Dihydro-orotate
1.3.1.14
1.3.98.1
1.3.5.2
Orotate
2.4.2.10
Orotidine-5P
4.1.1.23
2.1.3.2
Carbamoyl-P
6.3.5.5
Arginine biosynthesis
L-Glutamine
Alanine, aspartate and glutamate metabolism
3.6.1.17
UppppU
2.7.7.8
2.7.7.6
UTP
2.7.4.6
UDP
3.6.1.5
3.6.1.39
3.6.1.6
2.7.4.14
2.7.4.22
3.6.1.19
UMP
3.5.4.13
6.3.4.2
RNA
CTP
CDP
CMP
3.6.1.8
UDP-glucose (extracellular)
UTP (extracellular)
CTP (extracellular)
3'-UMP
3.1.4.16
2',3'-Cyclic UMP
3.1.3.6
2.7.1.48
3.1.3.5
Uridine
2.4.2.3
3.2.2.3
2.4.2.9
Uracil
3.5.4.5
3.5.4.1
2.4.2.2
3.2.2.8
Cytidine
Cytosine
3.2.2.10
2',3'-Cyclic CMP
3'-CMP
4.2.1.70
3.2.-.-
Pseudouridine-5'-phosphate
2.7.1.83
Pseudouridine
1.3.1.1
1.3.1.2
Dihydrouracil
3.5.2.2
3-Ureido-propionate
3.5.1.6
β-Alanine
β-Alanine metabolism
Barbiturate
1.17.99.4
3.5.2.1
3-Oxo-3-ureido-propanoate
3.5.1.95
Malonate
Urea
Ureidoacrylate
3.5.1.110
Carbamate
1.14.99.46
(Z)-3-Ureidoacrylate-peracid
(Z)-3-Peroxy-aminoacrylate
RutC
Aminoacrylate
RutD
Malonate-semialdehyde
YdfG
RutE
3-Hydroxy-propionate
Thioredoxin
Thioredoxin disulfide
1.8.1.9
1.17.4.2
1.17.4.1
dCTP
dCDP
dCMP
3.6.1.12
2.1.2.8
5-Hydroxymethyl-deoxycytidylate
2'-Deoxy-5-hydroxy-methylcytidine-5'-triphosphate
2'-Deoxy-5-hydroxy-methylcytidine-5'-diphosphate
2.7.1.74
Deoxycytidine
3.5.4.30
3.5.4.12
2.7.7.7
dUTP
dUDP
dUMP
2.7.4.9
3.6.1.23
2.7.1.21
Deoxyuridine
2.4.2.1
2.4.2.4
2.1.1.45
2.1.1.148
5-Methylbarbiturate
Methylmalonate
5-Methylcytosine
DNA
dTTP
dTDP
dTMP
Thymidine
2.4.2.6
Thymine
2-Deoxy-D-ribose-1P
Dihydro-thymine
3-Ureidoisobutyrate
3-Amino-isobutanoate
Valine, leucine and isoleucine metabolism
Data on KEGG graph
Rendered by Pathview

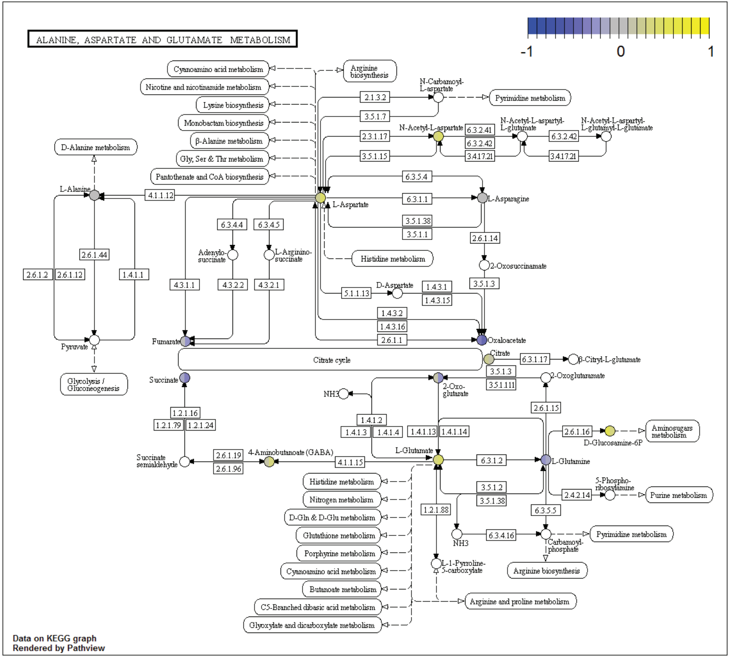

ALANINE, ASPARTATE AND GLUTAMATE METABOLISM
-1
0
1
Cyanoamino acid metabolism
Nicotine and nicotinamide metabolism
Lysine biosynthesis
Monobactam biosynthesis
β-Alanine metabolism
Gly, Ser & Thr metabolism
Pantothenate and CoA biosynthesis
Arginine biosynthesis
N-Carbamoyl-L-aspartate
Pyrimidine metabolism
2.1.3.2
3.5.1.7
2.3.1.17
3.5.1.15
N-Acetyl-L-aspartate
6.3.2.41
6.3.2.42
3.4.17.21
N-Acetyl-L-aspartyl-L-glutamate
6.3.2.42
3.4.17.21
N-Acetyl-L-aspartyl-L-glutamyl-L-glutamate
D-Alanine metabolism
L-Alanine
4.1.1.12
L-Aspartate
6.3.5.4
6.3.1.1
L-Asparagine
3.5.1.38
3.5.1.1
2.6.1.14
6.3.4.4
6.3.4.5
Adenylo-succinate
L-Argininosuccinate
Histidine metabolism
2-Oxosuccinamate
2.6.1.44
2.6.1.2
2.6.1.12
1.4.1.1
4.3.1.1
4.3.2.2
4.3.2.1
5.1.1.13
D-Aspartate
1.4.3.1
1.4.3.15
3.5.1.3
1.4.3.2
1.4.3.16
2.6.1.1
Oxaloacetate
Pyruvate
Fumarate
Citrate cycle
Citrate
6.3.1.17
β-Citryl-L-glutamate
Glycolysis / Gluconeogenesis
Succinate
3.5.1.3
3.5.1.111
2-Oxoglutaramate
2-Oxo-glutarate
NH3
1.2.1.16
1.2.1.79
1.2.1.24
1.4.1.2
1.4.1.3
1.4.1.4
1.4.1.13
1.4.1.14
2.6.1.15
2.6.1.16
D-Glucosamine-6P
Aminosugars metabolism
Succinate semialdehyde
2.6.1.19
2.6.1.96
4-Aminobutanoate (GABA)
4.1.1.15
L-Glutamate
6.3.1.2
L-Glutamine
Histidine metabolism
Nitrogen metabolism
D-Gln & D-Glu metabolism
Glutathione metabolism
Porphyrine metabolism
Cyanoamino acid metabolism
Butanoate metabolism
C5-Branched dibasic acid metabolism
Glyoxylate and dicarboxylate metabolism
3.5.1.2
3.5.1.38
5-Phospho-ribosylamine
2.4.2.14
Purine metabolism
1.2.1.88
6.3.5.5
NH3
6.3.4.16
Carbamoyl-phosphate
Pyrimidine metabolism
L-1-Pyrroline-5-carboxylate
Arginine biosynthesis
Arginine and proline metabolism
Data on KEGG graph
Rendered by Pathview

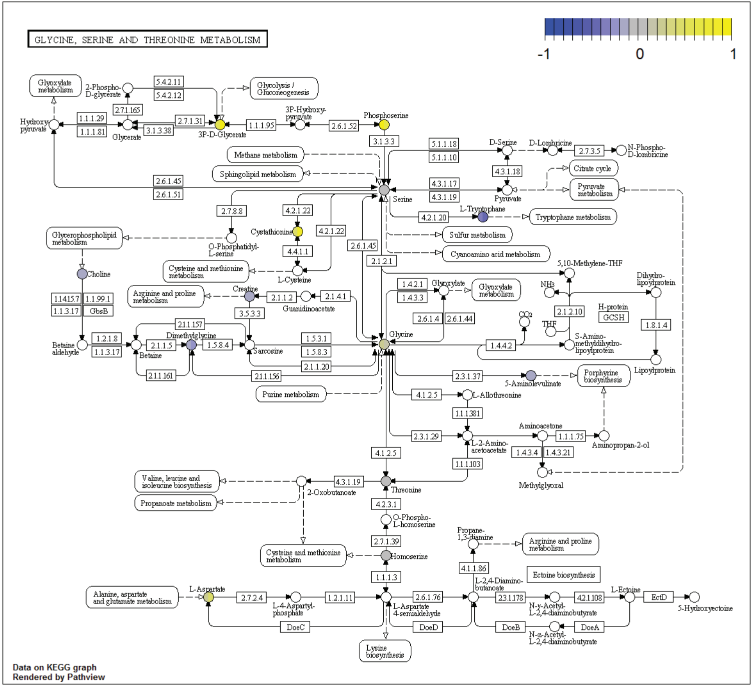
GLYCINE, SERINE AND THREONINE METABOLISM
-1
0
1
Glyoxylate metabolism
2-Phospho-D-glycerate
5.4.2.11
5.4.2.12
Glycolysis / Gluconeogenesis
2.7.1.165
Hydroxy-pyruvate
1.1.1.29
1.1.1.81
Glycerate
3.1.3.38
2.7.1.31
3P-D-Glycerate
1.1.1.95
3P-Hydroxy-pyruvate
2.6.1.52
Phosphoserine
3.1.3.3
5.1.1.18
5.1.1.10
D-Serine
D-Lombricine
2.7.3.5
N-Phospho-D-lombricine
Methane metabolism
Sphingolipid metabolism
4.3.1.18
Citrate cycle
2.6.1.45
2.6.1.51
Serine
4.3.1.17
4.3.1.19
Pyruvate
Pyruvate metabolism
2.7.8.8
4.2.1.22
L-Tryptophane
4.2.1.20
Tryptophane metabolism
Glycerophospholipid metabolism
Cystathionine
4.2.1.22
O-Phosphatidyl-L-serine
Sulfur metabolism
2.6.1.45
4.4.1.1
Cyanoamino acid metabolism
Choline
Cysteine and methionine metabolism
L-Cysteine
2.1.2.1
5,10-Methylene-THF
1.4.2.1
Glyoxylate
Glyoxylate metabolism
NH3
Dihydro-lipoylprotein
1.14.15.7
1.1.99.1
Arginine and proline metabolism
Creatine
2.1.1.2
Guanidinoacetate
2.1.4.1
1.4.3.3
1.1.3.17
GbsB
3.5.3.3
2.6.1.4
2.6.1.44
2.1.2.10
H-protein
GCSH
CO2
THF
1.8.1.4
2.1.1.157
Dimethylglycine
Glycine
Betaine aldehyde
1.2.1.8
1.1.3.17
2.1.1.5
Betaine
1.5.8.4
Sarcosine
1.5.3.1
1.5.8.3
2.1.1.20
1.4.4.2
S-Amino-methyldihydro-lipoylprotein
Lipoylprotein
2.1.1.161
2.1.1.156
2.3.1.37
5-Aminolevulinate
Porphyrine biosynthesis
Purine metabolism
4.1.2.5
L-Allothreonine
1.1.1.381
2.3.1.29
L-2-Amino-acetoacetate
Aminoacetone
1.1.1.75
Aminopropan-2-ol
4.1.2.5
1.4.3.4
1.4.3.21
1.1.1.103
Methylglyoxal
Valine, leucine and isoleucine biosynthesis
4.3.1.19
2-Oxobutanoate
Threonine
Propanoate metabolism
4.2.3.1
O-Phospho-L-homoserine
2.7.1.39
Cysteine and methionine metabolism
Homoserine
Propane-1,3-diamine
Arginine and proline metabolism
4.1.1.86
Ectoine biosynthesis
1.1.1.3
L-2,4-Diamino-butanoate
Alanine, aspartate and glutamate metabolism
L-Aspartate
2.7.2.4
L-4-Aspartyl-phosphate
1.2.1.11
L-Aspartate 4-semialdehyde
2.6.1.76
2.3.1.178
N-γ-Acetyl-L-2,4-diaminobutyrate
4.2.1.108
L-Ectoine
EctD
5-Hydroxyectoine
DoeC
DoeD
DoeB
N-α-Acetyl-L-2,4-diaminobutyrate
DoeA
Lysine biosynthesis
Data on KEGG graph
Rendered by Pathview

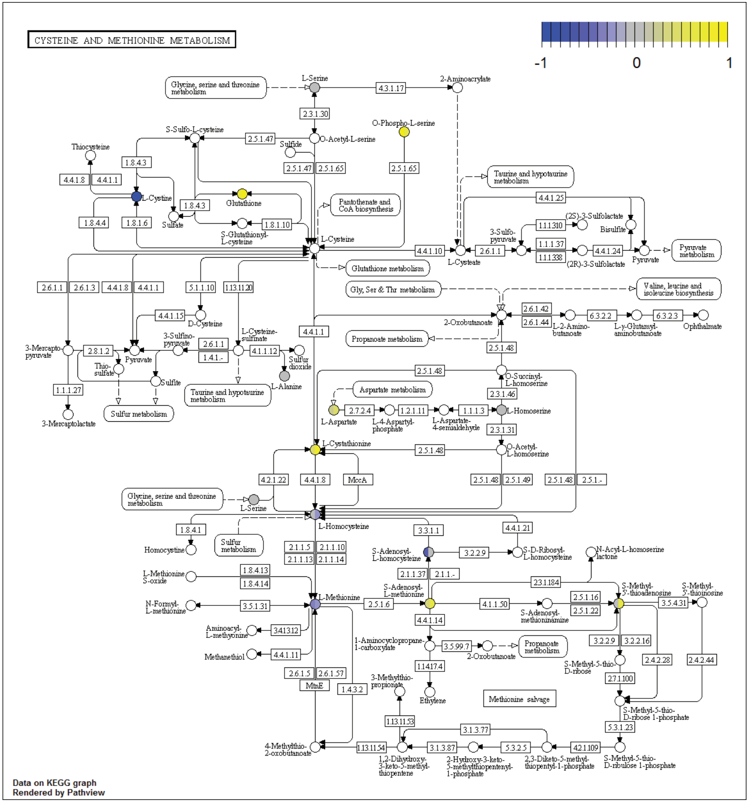
CYSTEINE AND METHIONINE METABOLISM
-1
0
1
Glycine, serine and threonine metabolism
L-Serine
4.3.1.17
2-Aminoacrylate
2.3.1.30
O-Phospho-L-serine
S-Sulfo-L-cysteine
2.5.1.47
O-Acetyl-L-serine
Sulfide
Thiocysteine
1.8.4.3
2.5.1.47
2.5.1.65
2.5.1.65
4.4.1.8
4.4.1.1
Taurine and hypotaurine metabolism
L-Cystine
Glutathione
1.8.4.3
Pantothenate and CoA biosynthesis
4.4.1.25
(2S)-3-Sulfolactate
1.1.1.310
Bisulfite
1.8.4.4
1.8.1.6
Sulfate
S-Glutathionyl-L-cysteine
1.8.1.10
L-Cysteine
3-Sulfo-pyruvate
1.1.1.37
4.4.1.10
2.6.1.1
4.4.1.24
Pyruvate
Pyruvate metabolism
L-Cysteate
1.1.1.338
(2R)-3-Sulfolactate
Glutathione metabolism
2.6.1.1
2.6.1.3
4.4.1.8
4.4.1.1
5.1.1.10
1.13.11.20
Gly, Ser & Thr metabolism
Valine, leucine and isoleucine biosynthesis
4.4.1.15
D-Cysteine
2.6.1.42
2.6.1.44
6.3.2.2
6.3.2.3
2-Oxobutanoate
L-2-Amino-butanoate
L-γ-Glutamyl-aminobutanoate
Ophthalmate
4.4.1.1
3-Sulfino-pyruvate
L-Cysteine-sulfinate
Propanoate metabolism
3-Mercapto-pyruvate
2.8.1.2
Pyruvate
2.6.1.1
1.4.1.-
4.1.1.12
Sulfur dioxide
2.5.1.48
Thio-sulfate
Sulfite
L-Alanine
O-Succinyl-L-homoserine
1.1.1.27
Taurine and hypotaurine metabolism
Aspartate metabolism
2.3.1.46
Sulfur metabolism
3-Mercaptolactate
2.7.2.4
1.2.1.11
1.1.1.3
L-Homoserine
L-Aspartate
L-4-Aspartyl-phosphate
L-Aspartate-4-semialdehyde
2.3.1.31
L-Cystathionine
2.5.1.48
O-Acetyl-L-homoserine
4.2.1.22
4.4.1.8
MccA
2.5.1.48
2.5.1.49
2.5.1.48
2.5.1.-
Glycine, serine and threonine metabolism
L-Serine
L-Homocysteine
1.8.4.1
Sulfur metabolism
3.3.1.1
4.4.1.21
Homocystine
2.1.1.5
2.1.1.10
2.1.1.13
2.1.1.14
S-Adenosyl-L-homocysteine
3.2.2.9
S-D-Ribosyl-L-homocysteine
N-Acyl-L-homoserine lactone
L-Methionine S-oxide
1.8.4.13
1.8.4.14
2.1.1.37
2.1.1.-
2.3.1.184
S-Adenosyl-L-methionine
S-Methyl-5'-thioadenosine
S-Methyl-5'-thioinosine
L-Methionine
2.5.1.6
4.1.1.50
2.5.1.16
2.5.1.22
3.5.4.31
N-Formyl-L-methionine
3.5.1.31
S-Adenosyl-methioninamine
4.4.1.14
Aminoacyl-L-methionine
3.4.13.12
1-Aminocyclopropane-1-carboxylate
3.5.99.7
2-Oxobutanoate
Propanoate metabolism
3.2.2.9
3.2.2.16
Methanethiol
4.4.1.11
1.14.17.4
S-Methyl-5-thio-D-ribose
2.4.2.28
2.4.2.44
2.6.1.5
2.6.1.57
3-Methylthio-propionate
2.7.1.100
MtnE
1.4.3.2
Ethylene
Methionine salvage
S-Methyl-5-thio-D-ribose 1-phosphate
1.13.11.53
3.1.3.77
5.3.1.23
4-Methylthio-2-oxobutanoate
1.13.11.54
3.1.3.87
5.3.2.5
4.2.1.109
1,2-Dihydroxy-3-keto-5-methyl-thiopentene
2-Hydroxy-3-keto-5-methylthiopentenyl-1-phosphate
2,3-Diketo-5-methyl-thiopentyl-1-phosphate
S-Methyl-5-thio-D-ribulose 1-phosphate
Data on KEGG graph
Rendered by Pathview

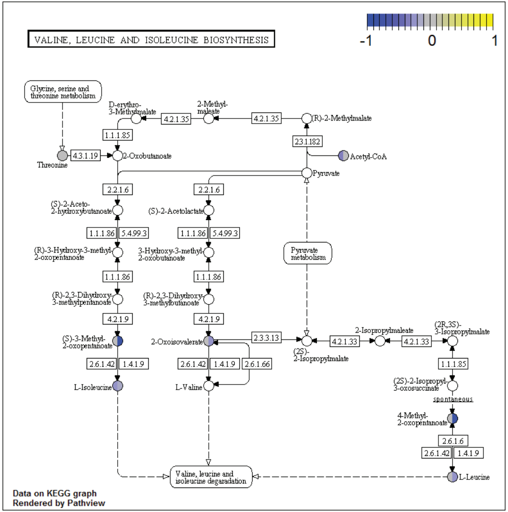

VALINE, LEUCINE AND ISOLEUCINE BIOSYNTHESIS
-1
0
1
Glycine, serine and threonine metabolism
D-erythro-3-Methylmalate
2-Methyl-maleate
(R)-2-Methylmalate
4.2.1.35
1.1.1.85
2.3.1.182
Acetyl-CoA
Threonine
4.3.1.19
2-Oxobutanoate
Pyruvate
2.2.1.6
(S)-2-Aceto-2-hydroxybutanoate
(S)-2-Acetolactate
1.1.1.86
5.4.99.3
(R)-3-Hydroxy-3-methyl-2-oxopentanoate
3-Hydroxy-3-methyl-2-oxobutanoate
Pyruvate metabolism
(R)-2,3-Dihydroxy-3-methylpentanoate
(R)-2,3-Dihydroxy-3-methylbutanoate
4.2.1.9
(S)-3-Methyl-2-oxopentanoate
2-Oxoisovalerate
2.3.3.13
(2S)-2-Isopropylmalate
4.2.1.33
2-Isopropylmaleate
(2R,3S)-3-Isopropylmalate
2.6.1.42
1.4.1.9
2.6.1.66
L-Isoleucine
L-Valine
(2S)-2-Isopropyl-3-oxosuccinate
spontaneous
4-Methyl-2-oxopentanoate
2.6.1.6
L-Leucine
Valine, leucine and isoleucine degaradation
Data on KEGG graph
Rendered by Pathview

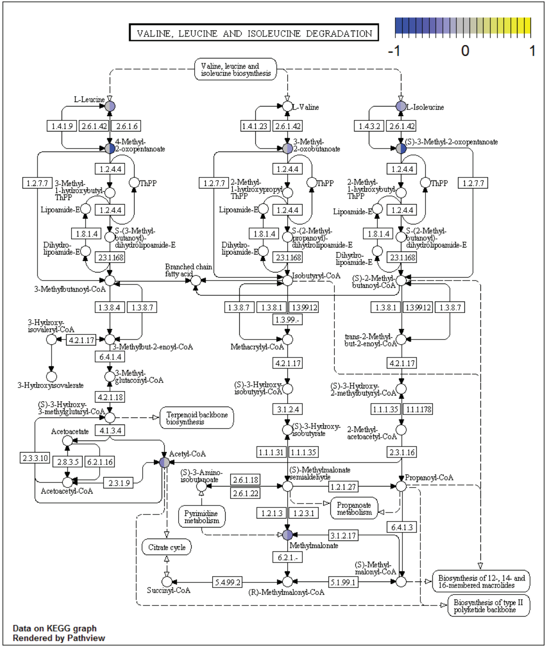

VALINE, LEUCINE AND ISOLEUCINE DEGRADATION
-1
0
1
Valine, leucine and isoleucine biosynthesis
L-Leucine
L-Valine
L-Isoleucine
1.4.1.9
2.6.1.42
2.6.1.6
1.4.1.23
1.4.3.2
4-Methyl-2-oxopentanoate
3-Methyl-2-oxobutanoate
(S)-3-Methyl-2-oxopentanoate
1.2.4.4
1.2.7.7
ThPP
3-Methyl-1-hydroxybutyl-ThPP
2-Methyl-1-hydroxypropyl-ThPP
2-Methyl-1-hydroxybutyl-ThPP
Lipoamide-E
1.8.1.4
Dihydro-lipoamide-E
S-(3-Methylbutanoyl)-dihydrolipoamide-E
S-(2-Methylpropanoyl)-dihydrolipoamide-E
S-(2-Methylbutanoyl)-dihydrolipoamide-E
2.3.1.168
Branched chain fatty acid
3-Methylbutanoyl-CoA
Isobutyryl-CoA
(S)-2-Methylbutanoyl-CoA
1.3.8.4
1.3.8.7
1.3.8.1
1.3.99.12
1.3.99.-
3-Hydroxy-isovaleryl-CoA
4.2.1.17
3-Methylbut-2-enoyl-CoA
Methacrylyl-CoA
trans-2-Methyl-but-2-enoyl-CoA
3-Hydroxyisovalerate
6.4.1.4
3-Methyl-glutaconyl-CoA
(S)-3-Hydroxy-isobutyryl-CoA
(S)-3-Hydroxy-2-methylbutyryl-CoA
4.2.1.18
(S)-3-Hydroxy-3-methylglutaryl-CoA
Terpenoid backbone biosynthesis
3.1.2.4
1.1.1.35
1.1.1.178
Acetoacetate
4.1.3.4
(S)-3-Hydroxy-isobutyrate
2-Methyl-acetoacetyl-CoA
2.3.3.10
2.8.3.5
6.2.1.16
Acetyl-CoA
1.1.1.31
2.3.1.16
(S)-3-Amino-isobutanoate
2.6.1.18
2.6.1.22
(S)-Methylmalonate semialdehyde
1.2.1.27
Propanoyl-CoA
Acetoacetyl-CoA
2.3.1.9
Propanoate metabolism
Pyrimidine metabolism
1.2.1.3
1.2.3.1
Methylmalonate
3.1.2.17
6.4.1.3
Citrate cycle
6.2.1.-
(S)-Methyl-malonyl-CoA
Succinyl-CoA
5.4.99.2
(R)-Methylmalonyl-CoA
5.1.99.1
Biosynthesis of 12-, 14- and 16-membered macrolides
Biosynthesis of type II polyketide backbone
Data on KEGG graph
Rendered by Pathview

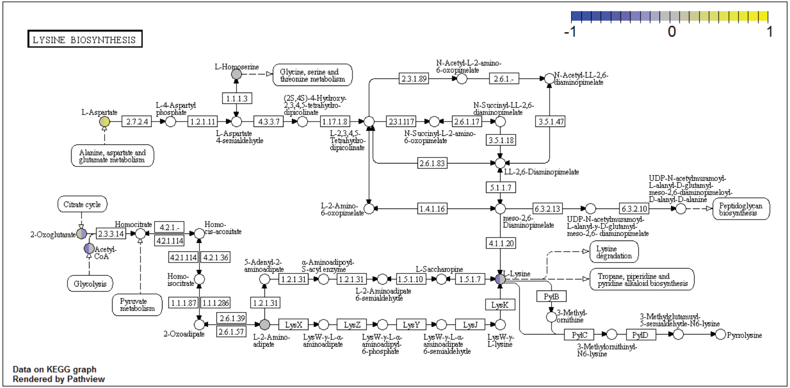

LYSINE BIOSYNTHESIS
-1
0
1
L-Homoserine
Glycine, serine and threonine metabolism
N-Acetyl-L-2-amino-6-oxopimelate
N-Acetyl-LL-2,6-diaminopimelate
L-Aspartate
L-4-Aspartyl phosphate
L-Aspartate 4-semialdehyde
(2S,4S)-4-Hydroxy-2,3,4,5-tetrahydro-dipicolinate
L-2,3,4,5-Tetrahydro-dipicolinate
N-Succinyl-LL-2,6-diaminopimelate
N-Succinyl-L-2-amino-6-oxopimelate
LL-2,6-Diaminopimelate
Alanine, aspartate and glutamate metabolism
Citrate cycle
2-Oxoglutarate
Acetyl-CoA
Homocitrate
Homo-cis-aconitate
Homo-isocitrate
2-Oxoadipate
Glycolysis
Pyruvate metabolism
L-2-Amino-6-oxopimelate
meso-2,6-Diaminopimelate
UDP-N-acetylmuramoyl-L-alanyl-D-glutamyl-meso-2,6-diaminopimeloyl-D-alanyl-D-alanine
UDP-N-acetylmuramoyl-L-alanyl-γ-D-glutamyl-meso-2,6-diaminopimelate
Peptidoglycan biosynthesis
Lysine degradation
Tropane, piperidine and pyridine alkaloid biosynthesis
5-Adenyl-2-aminoadipate
α-Aminoadipoyl-S-acyl enzyme
L-2-Aminoadipate 6-semialdehyde
L-Saccharopine
L-Lysine
L-2-Amino-adipate
LysW-γ-L-α-aminoadipate
LysW-γ-L-α-aminoadipyl-6-phosphate
LysW-γ-L-α-aminoadipate 6-semialdehyde
LysW-γ-L-lysine
3-Methyl-ornithine
3-Methylornithinyl-N6-lysine
3-Methylglutamuyl-5-semialdehyde-N6-lysine
Pyrrolysine
2.7.2.4
1.2.1.11
1.1.1.3
4.3.3.7
1.17.1.8
2.3.1.89
2.6.1.-
2.3.1.117
2.6.1.17
3.5.1.18
3.5.1.47
2.6.1.83
5.1.1.7
1.4.1.16
6.3.2.13
6.3.2.10
4.1.1.20
2.3.3.14
4.2.1.-
4.2.1.114
4.2.1.36
1.1.1.87
1.1.1.286
2.6.1.39
2.6.1.57
1.2.1.31
1.5.1.10
1.5.1.7
LysK
LysX
LysZ
LysY
LysJ
PylB
PylC
PylD
Data on KEGG graph
Rendered by Pathview

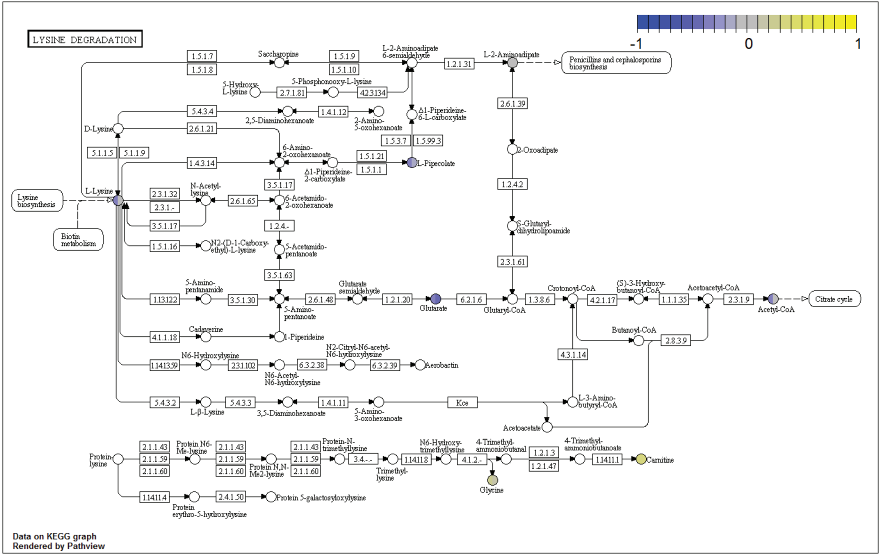
LYSINE DEGRADATION
-1
0
1
1.5.1.7
1.5.1.8
Saccharopine
1.5.1.9
1.5.1.10
L-2-Aminoadipate 6-semialdehyde
1.2.1.31
L-2-Aminoadipate
Penicillins and cephalosporins biosynthesis
5-Hydroxy-L-lysine
2.7.1.81
5-Phosphonooxy-L-lysine
4.2.3.134
5.4.3.4
2,5-Diaminohexanoate
1.4.1.12
2-Amino-5-oxohexanoate
Δ1-Piperideine-6-L-carboxylate
2.6.1.39
D-Lysine
2.6.1.21
6-Amino-2-oxohexanoate
1.5.3.7
1.5.99.3
2-Oxoadipate
5.1.1.5
5.1.1.9
1.4.3.14
Δ1-Piperideine-2-carboxylate
1.5.1.21
1.5.1.1
L-Pipecolate
3.5.1.17
1.2.4.2
Lysine biosynthesis
L-Lysine
2.3.1.32
2.3.1.-
N-Acetyl-lysine
2.6.1.65
6-Acetamido-2-oxohexanoate
3.5.1.17
1.2.4.-
S-Glutaryl-dihydrolipoamide
Biotin metabolism
1.5.1.16
N2-(D-1-Carboxyethyl)-L-lysine
5-Acetamido-pentanoate
3.5.1.63
2.3.1.61
1.13.12.2
5-Amino-pentanamide
3.5.1.30
5-Amino-pentanoate
2.6.1.48
Glutarate semialdehyde
1.2.1.20
Glutarate
6.2.1.6
Glutaryl-CoA
1.3.8.6
Crotonoyl-CoA
4.2.1.17
(S)-3-Hydroxy-butanoyl-CoA
1.1.1.35
Acetoacetyl-CoA
2.3.1.9
Acetyl-CoA
Citrate cycle
4.1.1.18
Cadaverine
1-Piperideine
Butanoyl-CoA
2.8.3.9
4.3.1.14
1.14.13.59
N6-Hydroxylysine
2.3.1.102
N6-Acetyl-N6-hydroxylysine
6.3.2.38
N2-Citryl-N6-acetyl-N6-hydroxylysine
6.3.2.39
Aerobactin
5.4.3.2
L-β-Lysine
5.4.3.3
3,5-Diaminohexanoate
1.4.1.11
5-Amino-3-oxohexanoate
Kce
L-3-Amino-butyryl-CoA
Acetoacetate
Protein lysine
2.1.1.43
2.1.1.59
2.1.1.60
Protein N6-Me-lysine
2.1.1.43
2.1.1.59
2.1.1.60
Protein N,N-Me2-lysine
2.1.1.43
2.1.1.59
2.1.1.60
Protein-N-trimethyllysine
3.4.-.-
Trimethyl-lysine
1.14.11.8
N6-Hydroxy-trimethyllysine
4.1.2.-
4-Trimethyl-ammoniobutanal
Glycine
1.2.1.3
1.2.1.47
4-Trimethyl-ammoniobutanoate
1.14.11.1
Carnitine
1.14.11.4
Protein erythro-5-hydroxylysine
2.4.1.50
Protein 5-galactosyloxylysine
Data on KEGG graph
Rendered by Pathview

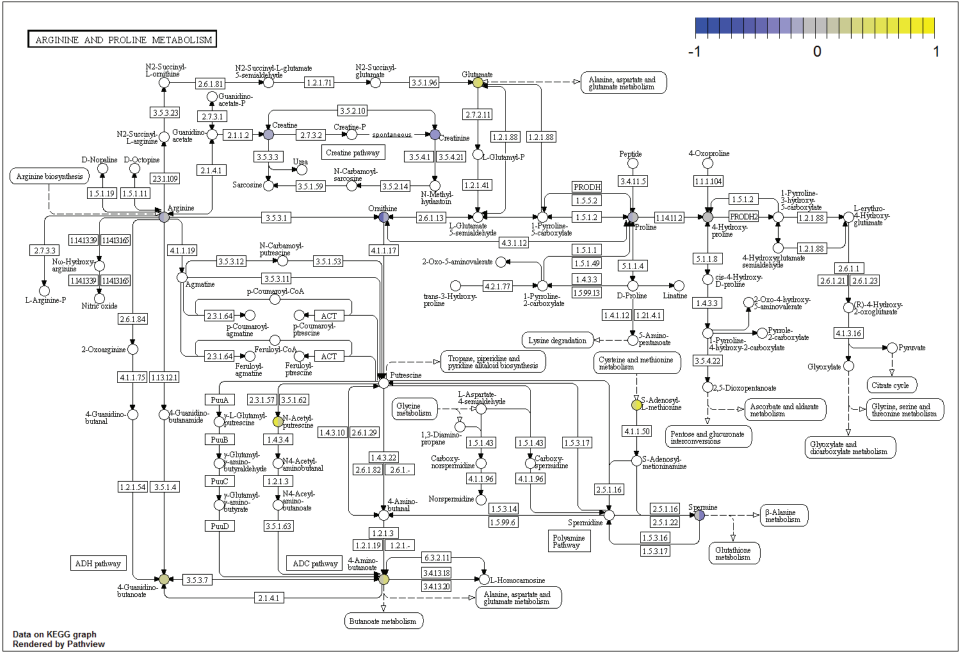
ARGININE AND PROLINE METABOLISM
-1
0
1
N2-Succinyl-L-ornithine
2.6.1.81
N2-Succinyl-L-glutamate 5-semialdehyde
1.2.1.71
N2-Succinyl-glutamate
3.5.1.96
Glutamate
Alanine, aspartate and glutamate metabolism
Guanidino-acetate-P
3.5.3.23
2.7.3.1
3.5.2.10
2.7.2.11
N2-Succinyl-L-arginine
Guanidino-acetate
2.1.1.2
Creatine
2.7.3.2
Creatine-P
spontaneous
Creatinine
1.2.1.88
1.2.1.88
3.5.3.3
Creatine pathway
3.5.4.1
3.5.4.21
L-Glutamyl-P
D-Nopaline
D-Octopine
Urea
2.1.4.1
N-Carbamoyl-sarcosine
Peptide
4-Oxoproline
Arginine biosynthesis
2.3.1.109
Sarcosine
3.5.1.59
3.5.2.14
1.2.1.41
3.4.11.5
1.1.1.104
1.5.1.19
1.5.1.11
N-Methyl-hydantoin
PRODH
1.5.1.2
1-Pyrroline-3-hydroxy-5-carboxylate
Arginine
Ornithine
1.5.5.2
L-erythro-4-Hydroxy-glutamate
3.5.3.1
2.6.1.13
1.5.1.2
1.14.11.2
PRODH2
1.2.1.88
L-Glutamate 5-semialdehyde
1-Pyrroline-5-carboxylate
Proline
4-Hydroxy-proline
2.7.3.3
1.14.13.39
1.14.13.165
4.3.1.12
4.1.1.19
N-Carbamoyl-putrescine
4.1.1.17
1.5.1.1
4-Hydroxyglutamate semialdehyde
1.2.1.88
Nω-Hydroxy-arginine
3.5.3.12
3.5.1.53
2-Oxo-5-aminovalerate
1.5.1.49
5.1.4
5.1.1.8
2.6.1.1
1.14.13.39
1.14.13.165
3.5.3.11
1.4.3.3
2.6.1.21
2.6.1.23
Agmatine
4.2.1.77
cis-4-Hydroxy-D-proline
L-Arginine-P
p-Coumaroyl-CoA
1.5.99.13
Linatine
Nitric oxide
trans-3-Hydroxy-proline
1-Pyrroline-2-carboxylate
D-Proline
1.4.3.3
2-Oxo-4-hydroxy-5-aminovalerate
(R)-4-Hydroxy-2-oxoglutarate
2.3.1.64
ACT
2.6.1.84
p-Coumaroyl-agmatine
p-Coumaroyl-ptrescine
1.4.1.12
1.21.4.1
Pyrrole-2-carboxylate
4.1.3.16
Lysine degradation
5-Amino-pentanoate
1-Pyrroline-4-hydroxy-2-carboxylate
2-Oxoarginine
Feruloyl-CoA
2.3.1.64
ACT
Pyruvate
Feruloyl-agmatine
Feruloyl-ptrescine
Tropane, piperidine and pyridine alkaloid biosynthesis
Cysteine and methionine metabolism
3.5.4.22
Glyoxylate
4.1.1.75
1.13.12.1
Putrescine
Citrate cycle
2,5-Dioxopentanoate
PuuA
2.3.1.57
3.5.1.62
L-Aspartate-4-semialdehyde
S-Adenosyl-L-methionine
4-Guanidino-butanal
4-Guanidino-butanamide
γ-L-Glutamyl-putrescine
N-Acetyl-putrescine
Glycine metabolism
Ascorbate and aldarate metabolism
Glycine, serine and threonine metabolism
1.4.3.10
2.6.1.29
1,3-Diamino-propane
Pentose and glucuronate interconversions
PuuB
1.4.3.4
1.5.1.43
1.5.1.43
1.5.3.17
4.1.1.50
Glyoxylate and dicarboxylate metabolism
γ-Glutamyl-γ-amino-butyraldehyde
N4-Acetyl-aminobutanal
1.4.3.22
Carboxy-norspermidine
Carboxy-spermidine
S-Adenosyl-methioninamine
PuuC
1.2.1.3
2.6.1.82
2.6.1.-
4.1.1.96
4.1.1.96
2.5.1.16
1.2.1.54
3.5.1.4
Norspermidine
γ-Glutamyl-γ-amino-butyrate
N4-Acetyl-amino-butanoate
4-Amino-butanal
1.5.3.14
2.5.1.16
Spermine
β-Alanine metabolism
1.5.99.6
Spermidine
2.5.1.22
PuuD
3.5.1.63
1.2.1.3
Polyamine Pathway
1.5.3.16
1.2.1.19
1.2.1.-
1.5.3.17
Glutathione metabolism
ADH pathway
ADC pathway
4-Amino-butanoate
6.3.2.11
3.4.13.18
3.5.3.7
L-Homocarnosine
3.4.13.20
4-Guanidino-butanoate
2.1.4.1
Alanine, aspartate and glutamate metabolism
Butanoate metabolism
Data on KEGG graph
Rendered by Pathview

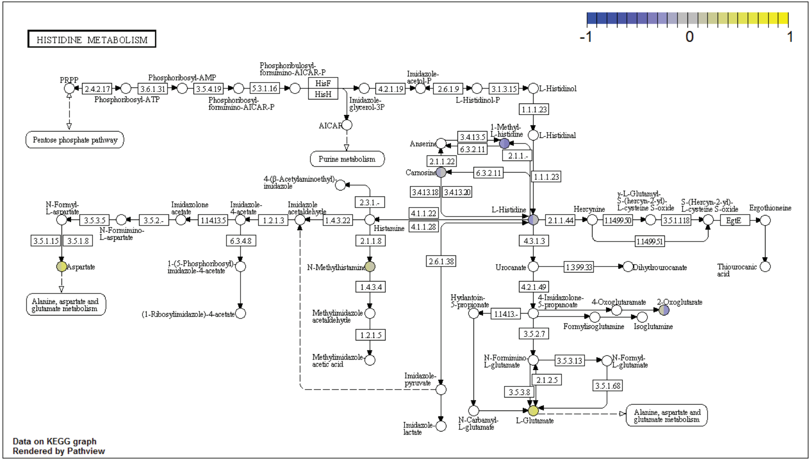
HISTIDINE METABOLISM
-1
0
1
PRPP
2.4.2.17
Phosphoribosyl-ATP
3.6.1.31
Phosphoribosyl-AMP
3.5.4.19
Phosphoribosyl-formimino-AICAR-P
5.3.1.16
Phosphoribulosyl-formimino-AICAR-P
HisF
HisH
AICAR
Purine metabolism
Imidazole-glycerol-3P
4.2.1.19
Imidazole-acetol-P
2.6.1.9
L-Histidinol-P
3.1.3.15
L-Histidinol
1.1.1.23
L-Histidinal
1.1.1.23
Pentose phosphate pathway
Anserine
3.4.13.5
6.3.2.11
1-Methyl-L-histidine
2.1.1.-
2.1.1.22
Carnosine
6.3.2.11
3.4.13.18
3.4.13.20
4-(β-Acetylaminoethyl)-imidazole
2.3.1.-
4.1.1.22
4.1.1.28
Histamine
L-Histidine
2.1.1.44
Hercynine
1.14.99.50
γ-L-Glutamyl-S-(hercyn-2-yl)-L-cysteine S-oxide
3.5.1.118
S-(Hercyn-2-yl)-L-cysteine S-oxide
EgtE
Ergothioneine
1.14.99.51
Thiourocanic acid
N-Formyl-L-aspartate
3.5.3.5
N-Formimino-L-aspartate
3.5.2.-
Imidazolone acetate
1.14.13.5
Imidazole-4-acetate
1.2.1.3
Imidazole acetaldehyde
1.4.3.22
3.5.1.15
3.5.1.8
6.3.4.8
2.1.1.8
Aspartate
1-(5-Phosphoribosyl)-imidazole-4-acetate
N-Methylhistamine
Alanine, aspartate and glutamate metabolism
(1-Ribosylimidazole)-4-acetate
1.4.3.4
Methylimidazole acetaldehyde
1.2.1.5
Methylimidazole-acetic acid
2.6.1.38
4.3.1.3
Urocanate
1.3.99.33
Dihydrourocanate
4.2.1.49
Hydantoin-5-propionate
1.14.13.-
4-Imidazolone-5-propanoate
4-Oxoglutaramate
2-Oxoglutarate
Formylisoglutamine
Isoglutamine
3.5.2.7
N-Formimino-L-glutamate
3.5.3.13
N-Formyl-L-glutamate
2.1.2.5
3.5.3.8
3.5.1.68
Imidazole-pyruvate
Imidazole-lactate
N-Carbamyl-L-glutamate
L-Glutamate
Alanine, aspartate and glutamate metabolism
Data on KEGG graph
Rendered by Pathview

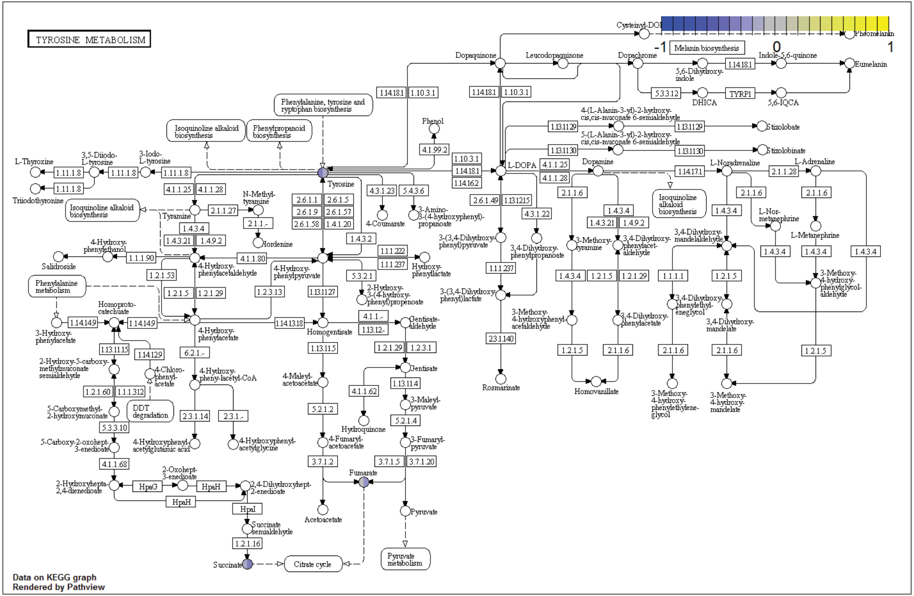
TYROSINE METABOLISM
-1
0
1
Cysteinyl-DOPA
Pheomelanin
Melanin biosynthesis
Dopaquinone
Leucodopaquinone
Dopachrome
Indole-5,6-quinone
Eumelanin
5,6-Dihydroxy-indole
1.14.18.1
5.3.3.12
DHICA
TYRP1
5,6-IQCA
1.14.18.1
1.10.3.1
Phenylalanine, tyrosine and tryptophan biosynthesis
Isoquinoline alkaloid biosynthesis
Phenylpropanoid biosynthesis
Phenol
4.1.99.2
4-(L-Alanin-3-yl)-2-hydroxy-cis,cis-muconate 6-semialdehyde
1.13.11.29
Stizolobate
5-(L-Alanin-3-yl)-2-hydroxy-cis,cis-muconate 6-semialdehyde
1.13.11.30
Stizolobinate
L-Thyroxine
3,5-Diiodo-L-tyrosine
3-Iodo-L-tyrosine
1.11.1.8
Triiodothyronine
Tyrosine
1.10.3.1
1.14.18.1
1.14.16.2
L-DOPA
4.1.1.25
4.1.1.28
Dopamine
1.14.17.1
L-Noradrenaline
2.1.1.28
L-Adrenaline
N-Methyl-tyramine
2.1.1.27
Tyramine
2.1.1.-
Hordenine
2.6.1.1
2.6.1.5
2.6.1.9
2.6.1.57
2.6.1.58
1.4.1.20
4.3.1.23
5.4.3.6
4-Coumarate
3-Amino-3-(4-hydroxyphenyl)-propanoate
2.6.1.49
1.13.12.15
4.3.1.22
2.1.1.6
1.4.3.4
1.4.3.21
1.4.9.2
Isoquinoline alkaloid biosynthesis
L-Nor-metanephrine
L-Metanephrine
4-Hydroxy-phenylethanol
Salidroside
1.1.1.90
4-Hydroxy-phenylacetaldehyde
4.1.1.80
4-Hydroxy-phenylpyruvate
1.4.3.2
1.1.1.222
1.1.1.237
Hydroxy-phenyllactate
3-(3,4-Dihydroxy-phenyl)pyruvate
3,4-Dihydroxy-phenylpropanoate
3-Methoxy-tyramine
3,4-Dihydroxy-phenylacet-aldehyde
3,4-Dihydroxy-mandelaldehyde
1.2.1.53
5.3.2.1
2-Hydroxy-3-(4-hydroxy-phenyl)propenoate
Phenylalanine metabolism
1.2.1.5
1.2.1.29
1.2.3.13
1.13.11.27
3-(3,4-Dihydroxy-phenyl)lactate
1.1.1.1
3-Methoxy-4-hydroxy-phenylglycol-aldehyde
3,4-Dihydroxy-phenylethyl-eneglycol
3,4-Dihydroxy-mandelate
Homoproto-catechuate
3-Hydroxy-phenylacetate
1.14.14.9
4-Hydroxy-phenylacetate
1.14.13.18
Homogentisate
4.1.1.-
1.13.12.-
Gentisate-aldehyde
3-Methoxy-4-hydroxyphenyl-acetaldehyde
3,4-Dihydroxy-phenylacetate
2.3.1.140
1.13.11.15
1.14.12.9
6.2.1.-
1.13.11.5
1.2.1.29
1.2.3.1
Gentisate
2-Hydroxy-5-carboxy-methylmuconate semialdehyde
4-Chloro-phenyl-acetate
4-Hydroxy-pheny-lacetyl-CoA
4-Maleyl-acetoacetate
4.1.1.62
1.13.11.4
Rosmarinate
Homovanillate
3-Methoxy-4-hydroxy-phenylethylene-glycol
3-Methoxy-4-hydroxy-mandelate
1.2.1.60
1.13.11.2
DDT degradation
5-Carboxymethyl-2-hydroxymuconate
2.3.1.14
2.3.1.-
5.2.1.2
Hydroquinone
3-Maleyl-pyruvate
5.2.1.4
5.3.3.10
5-Carboxy-2-oxohept-3-enedioate
4-Hydroxyphenyl-acetylglutamic acid
4-Hydroxyphenyl-acetylglycine
4-Fumaryl-acetoacetate
3-Fumaryl-pyruvate
4.1.1.68
3.7.1.2
3.7.1.5
3.7.1.20
2-Oxohept-3-enedioate
Fumarate
2-Hydroxyhepta-2,4-dienedioate
HpaG
HpaH
2,4-Dihydroxyhept-2-enedioate
HpaH
HpaI
Acetoacetate
Pyruvate
Succinate semialdehyde
1.2.1.16
Succinate
Citrate cycle
Pyruvate metabolism
Data on KEGG graph
Rendered by Pathview

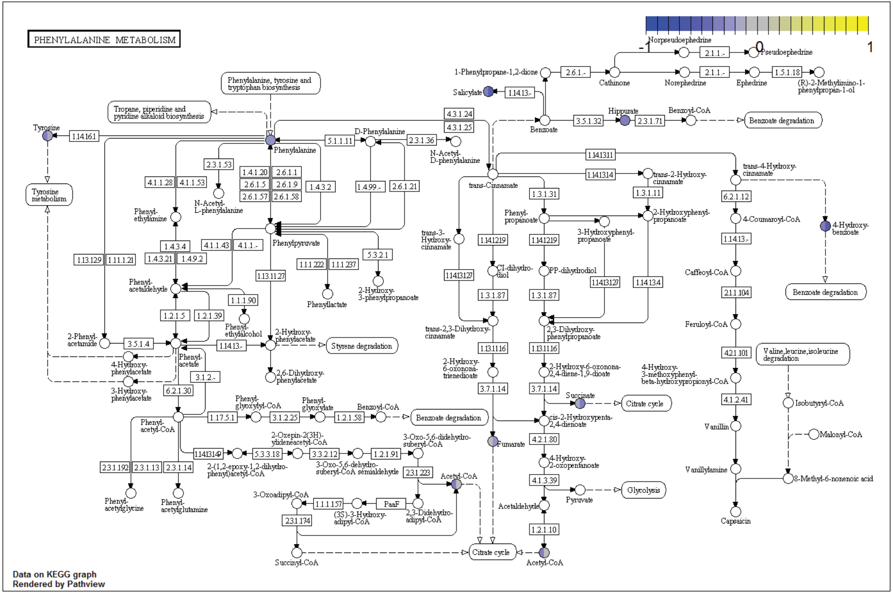
PHENYLALANINE METABOLISM
-1
0
1
Norpseudoephedrine
2.1.1.-
Pseudoephedrine
1-Phenylpropane-1,2-dione
2.6.1.-
Cathinone
2.1.1.-
Norephedrine
Ephedrine
1.5.1.18
(R)-2-Methylimino-1-phenylpropan-1-ol
Salicylate
1.14.13.-
Benzoate
3.5.1.32
Hippurate
2.3.1.71
Benzoyl-CoA
Benzoate degradation
Phenylalanine, tyrosine and tryptophan biosynthesis
Tropane, piperidine and pyridine alkaloid biosynthesis
Tyrosine
1.14.16.1
Tyrosine metabolism
4.3.1.24
4.3.1.25
D-Phenylalanine
5.1.1.11
2.3.1.36
N-Acetyl-D-phenylalanine
Phenylalanine
2.3.1.53
N-Acetyl-L-phenylalanine
4.1.1.28
4.1.1.53
1.4.1.20
2.6.1.1
2.6.1.5
2.6.1.9
2.6.1.57
2.6.1.58
1.4.3.2
1.4.99.-
2.6.1.21
Phenylethylamine
1.4.3.4
1.4.3.21
1.4.9.2
1.13.12.9
1.11.1.21
4.1.1.43
4.1.1.-
Phenylpyruvate
1.1.1.222
1.1.1.237
5.3.2.1
Phenyllactate
2-Hydroxy-3-phenylpropanoate
1.13.11.27
Phenylacetaldehyde
1.1.1.90
Phenylethylalcohol
1.2.1.5
1.2.1.39
2-Phenylacetamide
3.5.1.4
1.14.13.-
2-Hydroxyphenylacetate
Styrene degradation
4-Hydroxyphenylacetate
Phenylacetate
3.1.2.-
2,6-Dihydroxyphenylacetate
3-Hydroxyphenylacetate
6.2.1.30
Phenylglyoxylyl-CoA
Phenylglyoxylate
Benzoyl-CoA
Phenylacetyl-CoA
1.17.5.1
3.1.2.25
1.2.1.58
Benzoate degradation
2-Oxepin-2(3H)-ylideneacetyl-CoA
3-Oxo-5,6-didehydrosuberyl-CoA
1.14.13.149
5.3.3.18
3.3.2.12
1.2.1.91
2-(1,2-epoxy-1,2-dihydrophenyl)acetyl-CoA
3-Oxo-5,6-dehydrosuberyl-CoA semialdehyde
2.3.1.223
Acetyl-CoA
2.3.1.192
2.3.1.13
2.3.1.14
Phenylacetylglycine
Phenylacetylglutamine
3-Oxoadipyl-CoA
1.1.1.157
PaaF
(3S)-3-Hydroxyadipyl-CoA
2,3-Didehydroadipyl-CoA
2.3.1.174
Succinyl-CoA
Citrate cycle
1.14.13.11
1.14.13.14
trans-2-Hydroxycinnamate
trans-4-Hydroxycinnamate
trans-Cinnamate
1.3.1.31
1.3.1.11
6.2.1.12
Phenylpropanoate
2-Hydroxyphenylpropanoate
4-Coumaroyl-CoA
3-Hydroxyphenylpropanoate
4-Hydroxybenzoate
trans-3-Hydroxycinnamate
1.14.12.19
1.14.12.19
1.14.13.-
Caffeoyl-CoA
1.14.13.127
CI-dihydrodiol
PP-dihydrodiol
1.14.13.127
1.14.13.4
Benzoate degradation
2.1.1.104
1.3.1.87
1.3.1.87
trans-2,3-Dihydroxycinnamate
2,3-Dihydroxyphenylpropanoate
Feruloyl-CoA
1.13.11.16
1.13.11.16
4.2.1.101
Valine, leucine, isoleucine degradation
2-Hydroxy-6-oxonona-trienedioate
2-Hydroxy-6-oxonona-2,4-diene-1,9-dioate
4-Hydroxy-3-methoxyphenyl-beta-hydroxypropionyl-CoA
3.7.1.14
3.7.1.14
Succinate
Citrate cycle
4.1.2.41
Isobutyryl-CoA
cis-2-Hydroxypenta-2,4-dienoate
Vanillin
Fumarate
4.2.1.80
Malonyl-CoA
4-Hydroxy-2-oxopentanoate
Vanillylamine
4.1.3.39
8-Methyl-6-nonenoic acid
Pyruvate
Glycolysis
Acetaldehyde
Capsaicin
1.2.1.10
Citrate cycle
Acetyl-CoA
Data on KEGG graph
Rendered by Pathview

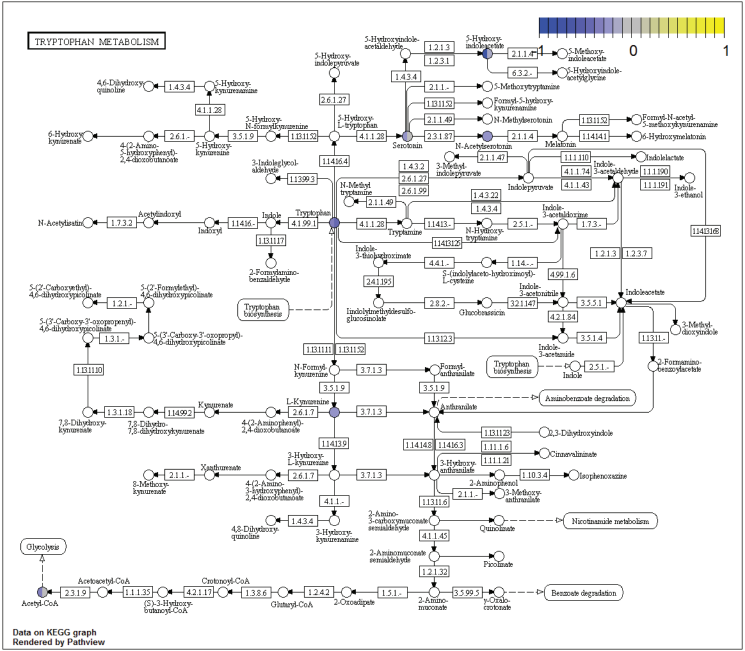

TRYPTOPHAN METABOLISM
-1
0
1
Tryptophan
Serotonin
N-Acetylserotonin
Melatonin
5-Hydroxy-L-tryptophan
5-Hydroxyindoleacetate
5-Hydroxyindole-acetaldehyde
5-Methoxyindoleacetate
5-Hydroxyindole-acetylglycine
5-Methoxytryptamine
Formyl-5-hydroxy-kynurenamine
N-Methylserotonin
Formyl-N-acetyl-5-methoxykynurenamine
6-Hydroxymelatonin
5-Hydroxy-indolepyruvate
4,6-Dihydroxy-quinoline
5-Hydroxy-kynurenamine
5-Hydroxy-N-formylkynurenine
5-Hydroxy-kynurenine
6-Hydroxy-kynurenate
4-(2-Amino-5-hydroxyphenyl)-2,4-dioxobutanoate
3-Indoleglycol-aldehyde
Indole
Indoxyl
Acetylindoxyl
N-Acetylisatin
2-Formylamino-benzaldehyde
N-Methyltryptamine
Tryptamine
N-Hydroxy-tryptamine
Indole-3-acetaldoxime
Indole-3-acetaldehyde
Indolepyruvate
3-Methyl-indolepyruvate
Indolelactate
Indole-3-ethanol
Indoleacetate
Indole-3-thiohydroximate
S-(indolylaceto-hydroximoyl)-L-cysteine
Indolylmethyldesulfo-glucosinolate
Glucobrassicin
Indole-3-acetonitrile
Indole-3-acetamide
3-Methyl-dioxyindole
2-Formamino-benzoylacetate
Tryptophan biosynthesis
5-(2'-Carboxyethyl)-4,6-dihydroxypicolinate
5-(2'-Formylethyl)-4,6-dihydroxypicolinate
5-(3'-Carboxy-3'-oxopropenyl)-4,6-dihydroxypicolinate
5-(3'-Carboxy-3'-oxopropyl)-4,6-dihydroxypicolinate
N-Formyl-kynurenine
Formyl-anthranilate
L-Kynurenine
Kynurenate
7,8-Dihydro-7,8-dihydroxykynurenate
7,8-Dihydroxy-kynurenate
4-(2-Aminophenyl)-2,4-dioxobutanoate
Anthranilate
Aminobenzoate degradation
2,3-Dihydroxyindole
Cinnavalininate
3-Hydroxy-anthranilate
2-Aminophenol
Isophenoxazine
3-Methoxy-anthranilate
3-Hydroxy-L-kynurenine
Xanthurenate
8-Methoxy-kynurenate
4-(2-Amino-3-hydroxyphenyl)-2,4-dioxobutanoate
4,8-Dihydroxy-quinoline
3-Hydroxy-kynurenamine
2-Amino-3-carboxymuconate semialdehyde
Quinolinate
Nicotinamide metabolism
2-Aminomuconate semialdehyde
Picolinate
2-Amino-muconate
γ-Oxalo-crotonate
Benzoate degradation
2-Oxoadipate
Glutaryl-CoA
Crotonoyl-CoA
(S)-3-Hydroxy-butanoyl-CoA
Acetoacetyl-CoA
Acetyl-CoA
Glycolysis
Data on KEGG graph
Rendered by Pathview

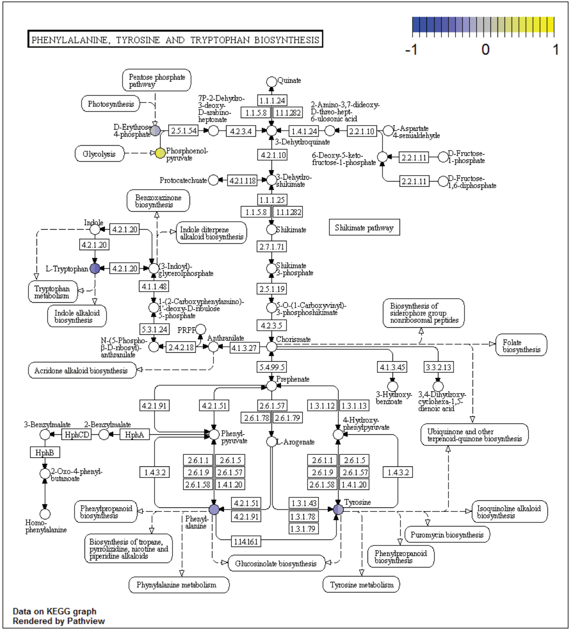

PHENYLALANINE, TYROSINE AND TRYPTOPHAN BIOSYNTHESIS
-1
0
1
Pentose phosphate pathway
Photosynthesis
D-Erythrose 4-phosphate
Glycolysis
Phosphoenol-pyruvate
2.5.1.54
4.2.3.4
7P-2-Dehydro-3-deoxy-D-arabino-heptonate
Quinate
1.1.1.24
1.1.5.8
1.1.1.282
3-Dehydroquinate
1.4.1.24
2-Amino-3,7-dideoxy-D-threo-hept-6-ulosonic acid
2.2.1.10
L-Aspartate 4-semialdehyde
4.2.1.10
6-Deoxy-5-keto-fructose-1-phosphate
2.2.1.11
D-Fructose-1-phosphate
D-Fructose-1,6-diphosphate
Protocatechuate
4.2.1.118
3-Dehydro-shikimate
1.1.1.25
Shikimate
Shikimate pathway
2.7.1.71
Shikimate 3-phosphate
2.5.1.19
5-O-(1-Carboxyvinyl)-3-phosphoshikimate
4.2.3.5
Chorismate
Benzoxazinone biosynthesis
Indole diterpene alkaloid biosynthesis
Indole
4.2.1.20
L-Tryptophan
(3-Indoyl)-glycerolphosphate
Tryptophan metabolism
Indole alkaloid biosynthesis
4.1.1.48
1-(2-Carboxyphenylamino)-1'-deoxy-D-ribulose 5-phosphate
5.3.1.24
PRPP
N-(5-Phospho-β-D-ribosyl)-anthranilate
2.4.2.18
Anthranilate
4.1.3.27
Acridone alkaloid biosynthesis
Biosynthesis of siderophore group nonribosomal peptides
Folate biosynthesis
5.4.99.5
4.1.3.45
3.3.2.13
3-Hydroxy-benzoate
3,4-Dihydroxy-cyclohexa-1,5-dienoic acid
Prephenate
4.2.1.91
4.2.1.51
2.6.1.57
2.6.1.78
2.6.1.79
1.3.1.12
1.3.1.13
4-Hydroxy-phenylpyruvate
Ubiquinone and other terpenoid-quinone biosynthesis
3-Benzylmalate
2-Benzylmalate
HphCD
HphA
HphB
Phenyl-pyruvate
L-Arogenate
2-Oxo-4-phenyl-butanoate
Homo-phenylalanine
1.4.3.2
2.6.1.1
2.6.1.5
2.6.1.9
2.6.1.58
1.4.1.20
1.3.1.43
1.3.1.78
1.3.1.79
Tyrosine
Phenyl-alanine
1.14.16.1
Phenylpropanoid biosynthesis
Biosynthesis of tropane, pyrrolizidine, nicotine and piperidine alkaloids
Glucosinolate biosynthesis
Isoquinoline alkaloid biosynthesis
Puromycin biosynthesis
Phynylalanine metabolism
Tyrosine metabolism
Data on KEGG graph
Rendered by Pathview

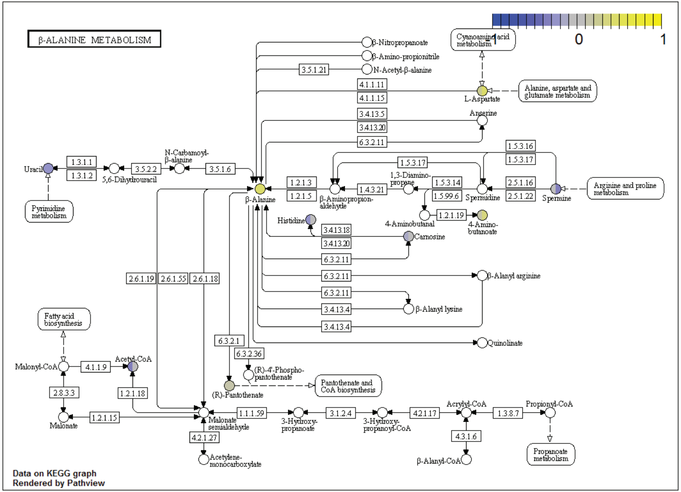
β-ALANINE METABOLISM
-1
0
1
β-Nitropropanoate
β-Amino-propionitrile
N-Acetyl-β-alanine
3.5.1.21
Cyanoamino acid metabolism
4.1.1.11
4.1.1.15
L-Aspartate
Alanine, aspartate and glutamate metabolism
3.4.13.5
3.4.13.20
6.3.2.11
Anserine
1.5.3.16
1.5.3.17
Uracil
1.3.1.1
1.3.1.2
5,6-Dihydrouracil
3.5.2.2
N-Carbamoyl-β-alanine
3.5.1.6
β-Alanine
1.2.1.3
1.2.1.5
β-Aminopropion-aldehyde
1.4.3.21
1,3-Diamino-propane
1.5.3.14
1.5.99.6
Spermidine
2.5.1.16
2.5.1.22
Spermine
Arginine and proline metabolism
Pyrimidine metabolism
4-Aminobutanal
1.2.1.19
4-Amino-butanoate
Histidine
3.4.13.18
3.4.13.20
Carnosine
6.3.2.11
6.3.2.11
β-Alanyl arginine
6.3.2.11
β-Alanyl lysine
3.4.13.4
3.4.13.4
Quinolinate
2.6.1.19
2.6.1.55
2.6.1.18
Fatty acid biosynthesis
6.3.2.1
6.3.2.36
(R)-4'-Phospho-pantothenate
Pantothenate and CoA biosynthesis
(R)-Pantothenate
Malonyl-CoA
4.1.1.9
Acetyl-CoA
2.8.3.3
1.2.1.18
Malonate
1.2.1.15
Malonate semialdehyde
1.1.1.59
3-Hydroxy-propanoate
3.1.2.4
3-Hydroxy-propanoyl-CoA
4.2.1.17
Acrylyl-CoA
1.3.8.7
Propionyl-CoA
4.2.1.27
4.3.1.6
Acetylene-monocarboxylate
β-Alanyl-CoA
Propanoate metabolism
Data on KEGG graph
Rendered by Pathview

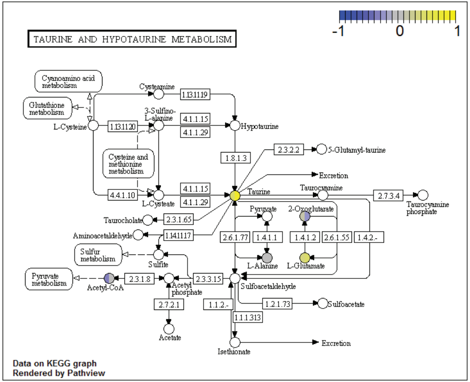
TAURINE AND HYPOTAURINE METABOLISM
-1
0
1
Cyanoamino acid metabolism
Glutathione metabolism
Cysteine and methionine metabolism
Sulfur metabolism
Pyruvate metabolism
Cysteamine
3-Sulfino-L-alanine
L-Cysteine
Hypotaurine
L-Cysteate
Taurine
Taurocyamine
Taurocyamine phosphate
5-Glutamyl-taurine
Excretion
Taurocholate
Aminoacetaldehyde
Sulfite
Pyruvate
2-Oxoglutarate
L-Alanine
L-Glutamate
Acetyl-CoA
Acetyl phosphate
Acetate
Sulfoacetaldehyde
Sulfoacetate
Isethionate
Excretion
1.13.11.19
1.13.11.20
4.1.1.15
4.1.1.29
4.4.1.10
4.1.1.15
4.1.1.29
1.8.1.3
2.3.2.2
2.7.3.4
2.3.1.65
1.14.11.17
2.6.1.77
1.4.1.1
1.4.1.2
2.6.1.55
1.4.2.-
2.3.1.8
2.3.3.15
2.7.2.1
1.1.2.-
1.1.1.313
1.2.1.73
Data on KEGG graph
Rendered by Pathview

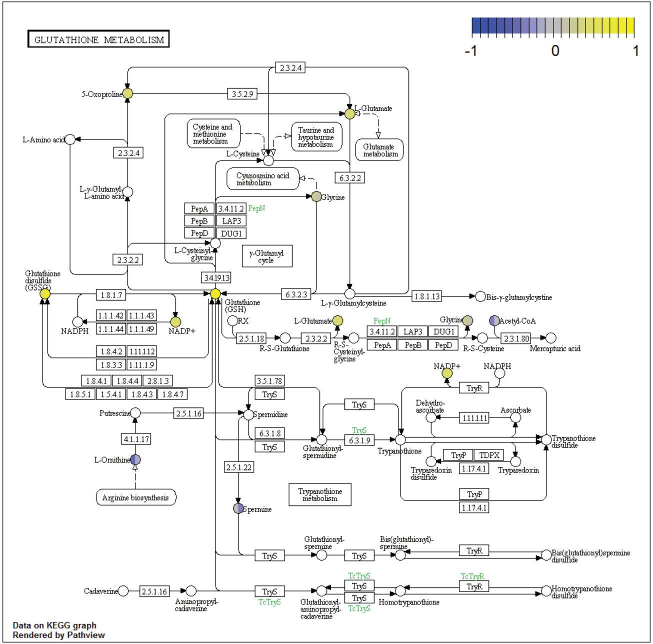
GLUTATHIONE METABOLISM
-1
0
1
5-Oxoproline
L-Glutamate
L-Amino acid
L-γ-Glutamyl-L-amino acid
L-Cysteine
Glycine
L-Cysteinyl-glycine
Glutathione disulfide (GSSG)
Glutathione (GSH)
NADPH
NADP+
L-γ-Glutamylcysteine
Bis-γ-glutamylcystine
RX
R-S-Glutathione
R-S-Cysteinyl-glycine
R-S-Cysteine
Acetyl-CoA
Mercapturic acid
Cysteine and methionine metabolism
Taurine and hypotaurine metabolism
Glutamate metabolism
Cyanoamino acid metabolism
γ-Glutamyl cycle
Putrescine
Spermidine
L-Ornithine
Arginine biosynthesis
Spermine
Glutathionyl-spermidine
Trypanothione
Trypanothione disulfide
Dehydro-ascorbate
Ascorbate
Tryparedoxin disulfide
Tryparedoxin
Trypanothione metabolism
Glutathionyl-spermine
Bis(glutathionyl)-spermine
Bis(glutathionyl)spermine disulfide
Cadaverine
Aminopropyl-cadaverine
Glutathionyl-aminopropyl-cadaverine
Homotrypanothione
Homotrypanothione disulfide
2.3.2.4
3.5.2.9
6.3.2.2
6.3.2.3
3.4.19.13
2.3.2.2
3.4.11.2
PepN
PepA
PepB
PepD
LAP3
DUG1
1.8.1.7
1.8.1.13
1.1.1.42
1.1.1.43
1.1.1.44
1.1.1.49
1.8.4.2
1.11.1.12
1.8.3.3
1.11.1.9
1.8.4.1
1.8.4.4
2.8.1.3
1.8.5.1
1.5.4.1
1.8.4.3
1.8.4.7
2.5.1.18
2.3.1.80
3.5.1.78
TryS
TcTryS
TryR
TcTryR
TryP
TDPX
1.11.1.11
1.17.4.1
2.5.1.16
4.1.1.17
6.3.1.8
6.3.1.9
2.5.1.22
Data on KEGG graph
Rendered by Pathview

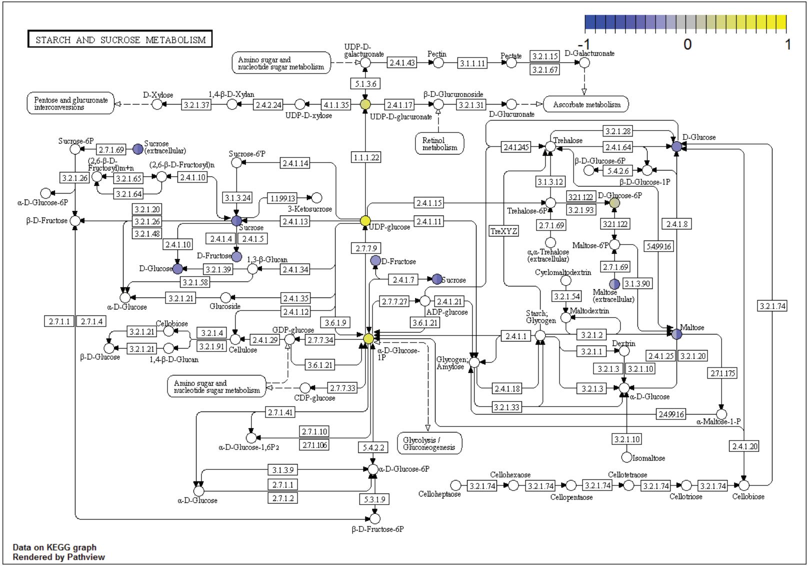
STARCH AND SUCROSE METABOLISM
-1
0
1
Amino sugar and nucleotide sugar metabolism
UDP-D-galacturonate
2.4.1.43
Pectin
3.1.1.11
Pectate
3.2.1.15
3.2.1.67
D-Galacturonate
5.1.3.6
Pentose and glucuronate interconversions
D-Xylose
3.2.1.37
1,4-β-D-Xylan
2.4.2.24
4.1.1.35
UDP-D-xylose
2.4.1.17
β-D-Glucuronoside
3.2.1.31
D-Glucuronate
UDP-D-glucuronate
Ascorbate metabolism
Retinol metabolism
1.1.1.22
Sucrose-6P
2.7.1.69
Sucrose (extracellular)
(2,6-β-D-Fructosyl)m+n
(2,6-β-D-Fructosyl)n
3.2.1.26
3.2.1.65
2.4.1.10
3.2.1.64
α-D-Glucose-6P
Sucrose-6'P
2.4.1.14
3.1.3.24
1.1.99.13
3-Ketosucrose
β-D-Fructose
3.2.1.20
3.2.1.26
3.2.1.48
Sucrose
2.4.1.13
2.4.1.4
2.4.1.5
2.4.1.10
D-Fructose
D-Glucose
3.2.1.39
1,3-β-Glucan
2.4.1.34
3.2.1.58
α-D-Glucose
3.2.1.21
Glucoside
2.4.1.35
2.4.1.12
2.7.1.1
2.7.1.4
Cellobiose
3.2.1.21
3.2.1.21
β-D-Glucose
1,4-β-D-Glucan
3.2.1.4
3.2.1.91
Cellulose
2.4.1.29
GDP-glucose
2.7.7.34
3.6.1.21
3.6.1.9
UDP-glucose
2.4.1.15
2.4.1.11
2.7.7.9
D-Fructose
2.4.1.7
Sucrose
2.7.7.27
ADP-glucose
2.4.1.21
3.6.1.21
α-D-Glucose-1P
Amino sugar and nucleotide sugar metabolism
CDP-glucose
2.7.7.33
2.7.1.41
2.7.1.10
2.7.1.106
α-D-Glucose-1,6P2
5.4.2.2
Glycolysis / Gluconeogenesis
α-D-Glucose-6P
3.1.3.9
2.7.1.1
2.7.1.2
α-D-Glucose
5.3.1.9
β-D-Fructose-6P
2.4.1.245
Trehalose
3.2.1.28
2.4.1.64
D-Glucose
β-D-Glucose-6P
5.4.2.6
β-D-Glucose-1P
3.1.3.12
Trehalose-6P
3.2.1.122
3.2.1.93
D-Glucose-6P
TreXYZ
2.7.1.69
α,α-Trehalose (extracellular)
3.2.1.122
Maltose-6'P
2.4.1.8
5.4.99.16
2.7.1.69
Cyclomaltodextrin
3.2.1.54
Maltose (extracellular)
3.1.3.90
Maltodextrin
Starch; Glycogen
2.4.1.1
3.2.1.2
Maltose
3.2.1.1
Dextrin
2.4.1.25
3.2.1.20
Glycogen; Amylose
2.4.1.18
3.2.1.3
3.2.1.10
3.2.1.3
α-D-Glucose
3.2.1.33
2.7.1.175
2.4.99.16
α-Maltose-1-P
3.2.1.74
3.2.1.10
Isomaltose
2.4.1.20
Celloheptaose
3.2.1.74
Cellohexaose
3.2.1.74
Cellopentaose
3.2.1.74
Cellotetraose
3.2.1.74
Cellotriose
3.2.1.74
Cellobiose
Data on KEGG graph
Rendered by Pathview

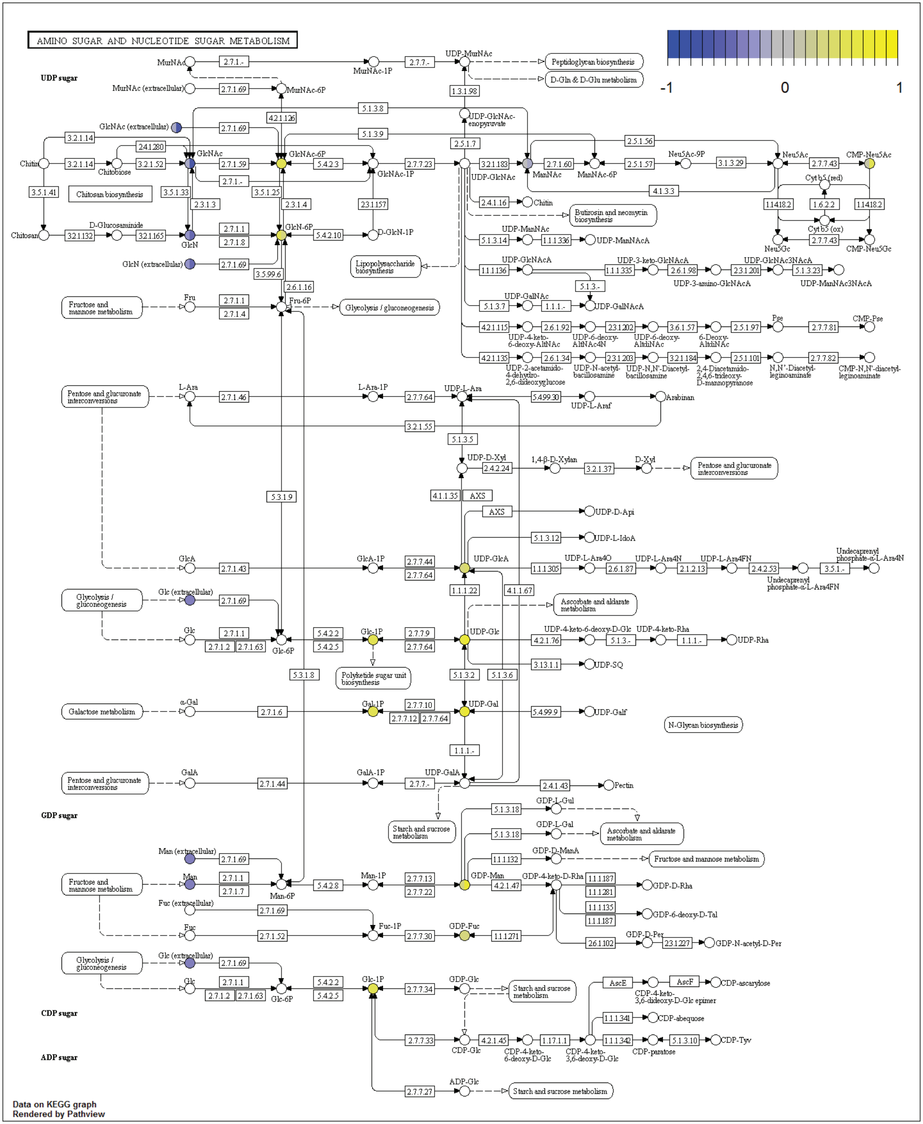
AMINO SUGAR AND NUCLEOTIDE SUGAR METABOLISM
-1
0
1
UDP sugar
GDP sugar
CDP sugar
ADP sugar
MurNAc
2.7.1.-
MurNAc-1P
2.7.7.-
UDP-MurNAc
Peptidoglycan biosynthesis
D-Gln & D-Glu metabolism
MurNAc (extracellular)
2.7.1.69
MurNAc-6P
1.3.1.98
5.1.3.8
4.2.1.126
UDP-GlcNAc-enopyruvate
GlcNAc (extracellular)
3.2.1.14
2.4.1.280
5.1.3.9
2.5.1.7
2.5.1.56
Chitin
Chitobiose
3.2.1.52
GlcNAc
2.7.1.59
GlcNAc-6P
5.4.2.3
GlcNAc-1P
2.7.7.23
UDP-GlcNAc
3.2.1.183
ManNAc
2.7.1.60
ManNAc-6P
2.5.1.57
Neu5Ac-9P
3.1.3.29
Neu5Ac
2.7.7.43
CMP-Neu5Ac
4.1.3.3
Cyt b5 (red)
1.14.18.2
1.6.2.2
Cyt b5 (ox)
Neu5Gc
CMP-Neu5Gc
Chitosan biosynthesis
3.5.1.41
3.5.1.33
3.5.1.25
2.3.1.3
2.3.1.4
2.3.1.157
2.4.1.16
Butirosin and neomycin biosynthesis
D-Glucosaminide
Chitosan
3.2.1.132
3.2.1.165
GlcN
2.7.1.8
GlcN-6P
5.4.2.10
D-GlcN-1P
UDP-ManNAc
5.1.3.14
1.1.1.336
UDP-ManNAcA
Lipopolysaccharide biosynthesis
UDP-GlcNAcA
1.1.1.136
UDP-3-keto-GlcNAcA
1.1.1.335
2.6.1.98
2.3.1.201
UDP-GlcNAc3NAcA
5.1.3.23
UDP-3-amino-GlcNAcA
UDP-ManNAc3NAcA
GlcN (extracellular)
3.5.99.6
2.6.1.16
5.1.3.-
Fructose and mannose metabolism
Fru
2.7.1.1
2.7.1.4
Fru-6P
Glycolysis / gluconeogenesis
UDP-GalNAc
5.1.3.7
1.1.1.-
UDP-GalNAcA
4.2.1.115
2.6.1.92
2.3.1.202
3.6.1.57
2.5.1.97
Pse
2.7.7.81
CMP-Pse
UDP-4-keto-6-deoxy-AltNAc
UDP-6-deoxy-AltNAc4N
UDP-6-deoxy-AltdiNAc
6-Deoxy-AltdiNAc
4.2.1.135
2.6.1.34
2.3.1.203
3.2.1.184
2.5.1.101
2.7.7.82
UDP-2-acetamido-4-dehydro-2,6-dideoxyglucose
UDP-N-acetyl-bacillosamine
UDP-N,N'-Diacetyl-bacillosamine
2,4-Diacetamido-2,4,6-trideoxy-D-mannopyranose
N,N'-Diacetyl-legionaminate
CMP-N,N'-diacetyl-legionaminate
Pentose and glucuronate interconversions
L-Ara
2.7.1.46
L-Ara-1P
2.7.7.64
UDP-L-Ara
5.4.99.30
UDP-L-Araf
Arabinan
3.2.1.55
5.1.3.5
UDP-D-Xyl
2.4.2.24
1,4-β-D-Xylan
3.2.1.37
D-Xyl
5.3.1.9
4.1.1.35
AXS
UDP-D-Api
5.1.3.12
UDP-L-IdoA
GlcA
2.7.1.43
GlcA-1P
2.7.7.44
UDP-GlcA
1.1.1.305
UDP-L-Ara4O
2.6.1.87
UDP-L-Ara4N
2.1.2.13
UDP-L-Ara4FN
2.4.2.53
Undecaprenyl phosphate-α-L-Ara4N
3.5.1.-
Undecaprenyl phosphate-α-L-Ara4FN
1.1.1.22
4.1.1.67
Ascorbate and aldarate metabolism
Glc (extracellular)
Glc
2.7.1.2
2.7.1.63
Glc-6P
5.4.2.2
5.4.2.5
Glc-1P
2.7.7.9
UDP-Glc
UDP-4-keto-6-deoxy-D-Glc
4.2.1.76
UDP-4-keto-Rha
UDP-Rha
3.13.1.1
UDP-SQ
5.3.1.8
Polyketide sugar unit biosynthesis
5.1.3.2
5.1.3.6
Galactose metabolism
α-Gal
2.7.1.6
Gal-1P
2.7.7.10
2.7.7.12
UDP-Gal
5.4.99.9
UDP-Galf
N-Glycan biosynthesis
GalA
2.7.1.44
GalA-1P
UDP-GalA
2.4.1.43
Pectin
GDP-L-Gul
5.1.3.18
GDP-L-Gal
Starch and sucrose metabolism
GDP-D-ManA
1.1.1.132
Man (extracellular)
Man
2.7.1.7
Man-6P
5.4.2.8
Man-1P
2.7.7.13
2.7.7.22
GDP-Man
4.2.1.47
GDP-4-keto-D-Rha
1.1.1.187
1.1.1.281
GDP-D-Rha
GDP-6-deoxy-D-Tal
Fuc (extracellular)
Fuc
2.7.1.52
Fuc-1P
2.7.7.30
GDP-Fuc
1.1.1.271
GDP-D-Per
2.6.1.102
2.3.1.227
GDP-N-acetyl-D-Per
2.7.7.34
GDP-Glc
AscE
AscF
CDP-ascarylose
CDP-4-keto-3,6-dideoxy-D-Glc epimer
1.1.1.341
CDP-abequose
2.7.7.33
CDP-Glc
4.2.1.45
CDP-4-keto-6-deoxy-D-Glc
1.17.1.1
CDP-4-keto-3,6-dideoxy-D-Glc
1.1.1.342
CDP-paratose
5.1.3.10
CDP-Tyv
ADP-Glc
2.7.7.27
Data on KEGG graph
Rendered by Pathview

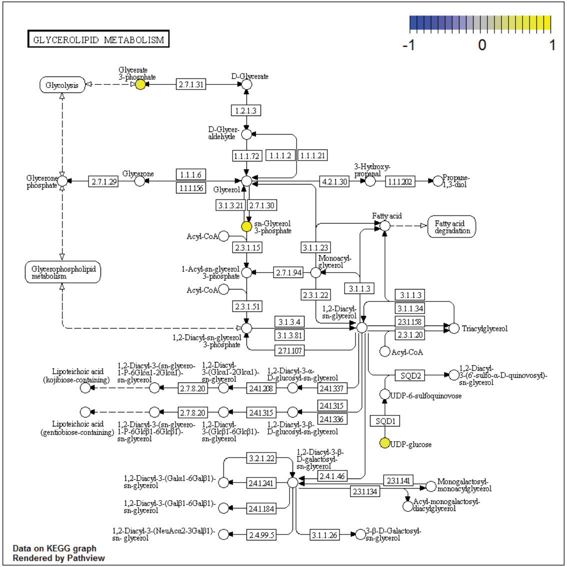
GLYCEROLIPID METABOLISM
-1
0
1
Glycolysis
Glycerate 3-phosphate
2.7.1.31
D-Glycerate
1.2.1.3
D-Glycer-aldehyde
1.1.1.72
1.1.1.2
1.1.1.21
Glycerone phosphate
2.7.1.29
Glycerone
1.1.1.6
1.1.1.156
Glycerol
4.2.1.30
3-Hydroxy-propanal
1.1.1.202
Propane-1,3-diol
3.1.3.21
2.7.1.30
sn-Glycerol 3-phosphate
Fatty acid
Fatty acid degradation
Acyl-CoA
2.3.1.15
3.1.1.23
Glycerophospholipid metabolism
1-Acyl-sn-glycerol 3-phosphate
2.7.1.94
Monoacyl-glycerol
Acyl-CoA
2.3.1.22
3.1.1.3
3.1.1.3
2.3.1.51
1,2-Diacyl-sn-glycerol
3.1.1.34
3.1.3.4
2.3.1.158
3.1.3.81
2.3.1.20
Triacylglycerol
1,2-Diacyl-sn-glycerol 3-phosphate
2.7.1.107
Acyl-CoA
Lipoteichoic acid (kojibiose-containing)
1,2-Diacyl-3-(sn-glycero-1-P-6Glcα1-2Glcα1)-sn-glycerol
1,2-Diacyl-3-(Glcα1-2Glcα1)-sn-glycerol
1,2-Diacyl-3-α-D-glucosyl-sn-glycerol
SQD2
1,2-Diacyl-3-(6'-sulfo-α-D-quinovosyl)-sn-glycerol
2.7.8.20
2.4.1.208
2.4.1.337
UDP-6-sulfoquinovose
2.4.1.315
2.7.8.20
2.4.1.315
2.4.1.336
SQD1
Lipoteichoic acid (gentiobiose-containing)
1,2-Diacyl-3-(sn-glycero-1-P-6Glcβ1-6Glcβ1)-sn-glycerol
1,2-Diacyl-3-(Glcβ1-6Glcβ1)-sn-glycerol
1,2-Diacyl-3-β-D-glucosyl-sn-glycerol
UDP-glucose
3.2.1.22
1,2-Diacyl-3-β-D-galactosyl-sn-glycerol
2.4.1.46
2.3.1.141
1,2-Diacyl-3-(Galα1-6Galβ1)-sn-glycerol
2.4.1.241
Monogalactosyl-monoacylglycerol
2.3.1.134
Acyl-monogalactosyl-diacylglycerol
1,2-Diacyl-3-(Galβ1-6Galβ1)-sn-glycerol
2.4.1.184
1,2-Diacyl-3-(NeuAcα2-3Galβ1)-sn- glycerol
2.4.99.5
3.1.1.26
3-β-D-Galactosyl-sn-glycerol
Data on KEGG graph
Rendered by Pathview

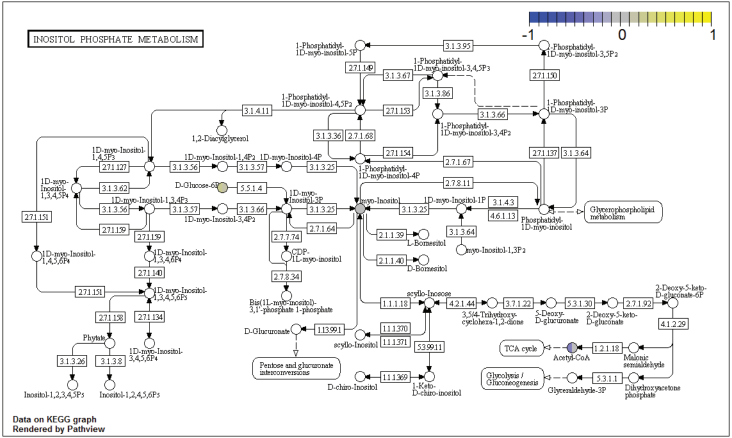
INOSITOL PHOSPHATE METABOLISM
-1
0
1
1-Phosphatidyl-1D-myo-inositol-5P
3.1.3.95
1-Phosphatidyl-1D-myo-inositol-3,5P2
2.7.1.149
3.1.3.67
1-Phosphatidyl-1D-myo-inositol-3,4,5P3
2.7.1.150
3.1.3.86
1-Phosphatidyl-1D-myo-inositol-4,5P2
3.1.4.11
2.7.1.153
3.1.3.66
1-Phosphatidyl-1D-myo-inositol-3P
1,2-Diacylglycerol
3.1.3.36
2.7.1.68
1-Phosphatidyl-1D-myo-inositol-3,4P2
1D-myo-Inositol-1,4,5P3
2.7.1.154
2.7.1.137
3.1.3.64
2.7.1.67
2.7.1.127
3.1.3.56
1D-myo-Inositol-1,4P2
3.1.3.57
1D-myo-Inositol-4P
3.1.3.25
1-Phosphatidyl-1D-myo-inositol-4P
1D-myo-Inositol-1,3,4,5P4
3.1.3.62
D-Glucose-6P
5.5.1.4
2.7.8.11
1D-myo-Inositol-3P
myo-Inositol
1D-myo-Inositol-1P
3.1.4.3
1D-myo-Inositol-1,3,4P3
3.1.3.56
3.1.3.57
3.1.3.66
3.1.3.25
3.1.3.25
4.6.1.13
Phosphatidyl-1D-myo-inositol
Glycerophospholipid metabolism
2.7.1.151
2.7.1.159
1D-myo-Inositol-3,4P2
2.7.1.159
2.7.7.74
2.7.1.64
2.1.1.39
L-Bornesitol
3.1.3.64
myo-Inositol-1,3P2
1D-myo-Inositol-1,4,5,6P4
1D-myo-Inositol-1,3,4,6P4
CDP-1L-myo-inositol
2.1.1.40
D-Bornesitol
2.7.1.140
2.7.8.34
2.7.1.151
1D-myo-Inositol-1,3,4,5,6P5
Bis(1L-myo-inositol)-3,1'-phosphate 1-phosphate
scyllo-Inosose
1.1.1.18
4.2.1.44
3.7.1.22
5.3.1.30
2.7.1.92
2-Deoxy-5-keto-D-gluconate-6P
3,5/4-Trihydroxy-cyclohexa-1,2-dione
5-Deoxy-D-glucuronate
2-Deoxy-5-keto-D-gluconate
2.7.1.158
2.7.1.134
D-Glucuronate
1.13.99.1
1.1.1.370
scyllo-Inositol
1.1.1.371
4.1.2.29
Phytate
1D-myo-Inositol-3,4,5,6P4
5.3.99.11
TCA cycle
Acetyl-CoA
1.2.1.18
Malonic semialdehyde
3.1.3.26
3.1.3.8
Pentose and glucuronate interconversions
D-chiro-Inositol
1.1.1.369
1-Keto-D-chiro-inositol
Glycolysis / Gluconeogenesis
Glyceraldehyde-3P
5.3.1.1
Dihydroxyacetone phosphate
Inositol-1,2,3,4,5P5
Inositol-1,2,4,5,6P5
Data on KEGG graph
Rendered by Pathview

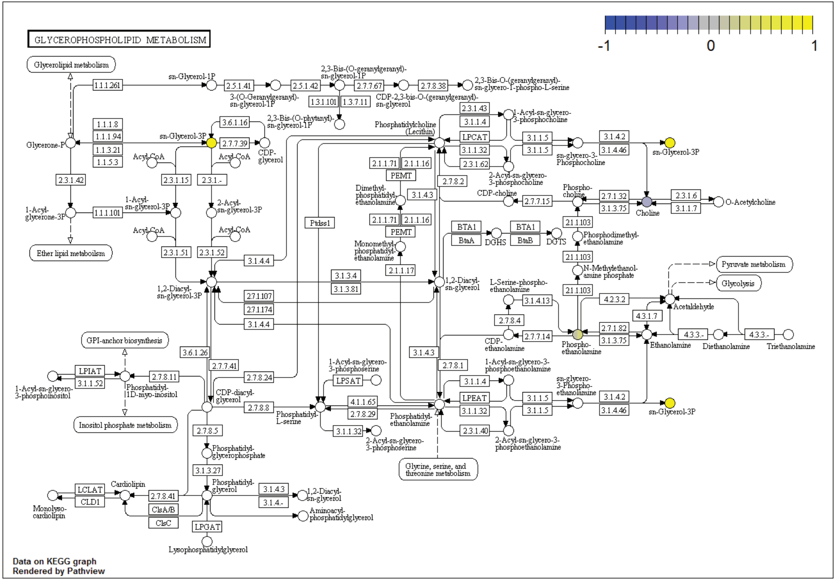

GLYCEROPHOSPHOLIPID METABOLISM
-1
0
1
Glycerolipid metabolism
Ether lipid metabolism
GPI-anchor biosynthesis
Inositol phosphate metabolism
Glycine, serine, and threonine metabolism
Pyruvate metabolism
Glycolysis
Glycerone-P
1-Acyl-glycerone-3P
sn-Glycerol-1P
3-(O-Geranylgeranyl)-sn-glycerol-1P
2,3-Bis-(O-geranylgeranyl)-sn-glycerol-1P
CDP-2,3-bis-O-(geranylgeranyl)-sn-glycerol
2,3-Bis-O-(geranylgeranyl)-sn-glycero-1-phospho-L-serine
2,3-Bis-(O-phytanyl)-sn-glycerol-1P
sn-Glycerol-3P
CDP-glycerol
Acyl-CoA
1-Acyl-sn-glycerol-3P
2-Acyl-sn-glycerol-3P
1,2-Diacyl-sn-glycerol-3P
Phosphatidylcholine (Lecithin)
1-Acyl-sn-glycero-3-phosphocholine
2-Acyl-sn-glycero-3-phosphocholine
sn-glycero-3-Phosphocholine
Dimethyl-phosphatidyl-ethanolamine
Monomethyl-phosphatidyl-ethanolamine
CDP-choline
Phospho-choline
Choline
O-Acetylcholine
DGHS
DGTS
Phosphodimethyl-ethanolamine
N-Methylethanol-amine phosphate
1,2-Diacyl-sn-glycerol
L-Serine-phospho-ethanolamine
Acetaldehyde
CDP-ethanolamine
Phospho-ethanolamine
Ethanolamine
Diethanolamine
Triethanolamine
1-Acyl-sn-glycero-3-phosphoethanolamine
sn-glycero-3-Phospho-ethanolamine
2-Acyl-sn-glycero-3-phosphoethanolamine
Phosphatidyl-ethanolamine
1-Acyl-sn-glycero-3-phosphoserine
2-Acyl-sn-glycero-3-phosphoserine
Phosphatidyl-L-serine
1-Acyl-sn-glycero-3-phosphoinositol
Phosphatidyl-1D-myo-inositol
CDP-diacyl-glycerol
Phosphatidyl-glycerophosphate
Phosphatidyl-glycerol
1,2-Diacyl-sn-glycerol
Aminoacyl-phosphatidylglycerol
Lysophosphatidylglycerol
Cardiolipin
Monolyso-cardiolipin
1.1.1.261
2.5.1.41
2.5.1.42
2.7.7.67
2.7.8.38
1.3.1.101
1.3.7.11
1.1.1.8
1.1.1.94
1.1.3.21
1.1.5.3
3.6.1.16
2.7.7.39
2.3.1.42
2.3.1.15
2.3.1.-
1.1.1.101
2.3.1.51
2.3.1.52
3.1.4.4
Ptdss1
2.7.1.107
2.7.1.174
3.1.3.4
3.1.3.81
3.1.4.4
2.3.1.43
3.1.1.4
LPCAT
3.1.1.32
2.3.1.62
3.1.1.5
3.1.1.5
3.1.4.2
3.1.4.46
2.1.1.71
2.1.1.16
PEMT
3.1.4.3
2.7.8.2
2.7.7.15
2.7.1.32
3.1.3.75
2.3.1.6
3.1.1.7
2.1.1.71
2.1.1.16
PEMT
2.1.1.17
BTA1
BtaA
BTA1
BtaB
2.1.1.103
2.1.1.103
2.1.1.103
3.1.4.13
2.7.8.4
4.2.3.2
4.3.1.7
2.7.1.82
3.1.3.75
2.7.7.14
4.3.3.-
4.3.3.-
3.1.4.3
2.7.8.1
3.6.1.26
2.7.7.41
2.7.8.24
LPIAT
3.1.1.52
2.7.8.11
LPSAT
4.1.1.65
2.7.8.29
3.1.1.32
3.1.1.4
LPEAT
3.1.1.32
2.3.1.40
3.1.1.5
3.1.1.5
3.1.4.2
3.1.4.46
2.7.8.8
2.7.8.5
3.1.3.27
LCLAT
CLD1
2.7.8.41
ClsA/B
ClsC
3.1.4.3
3.1.4.-
LPGAT
Data on KEGG graph
Rendered by Pathview

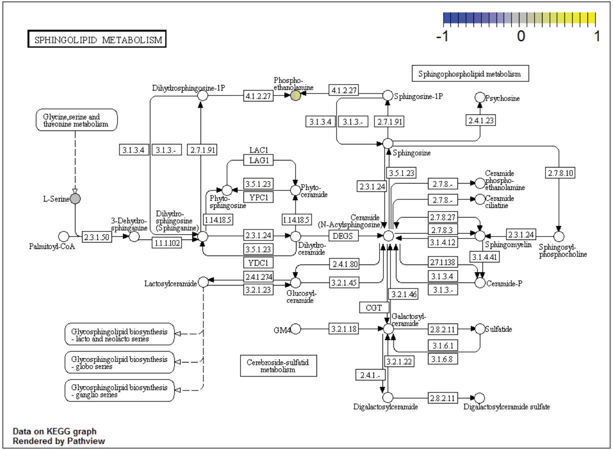
SPHINGOLIPID METABOLISM
-1
0
1
Sphingophospholipid metabolism
Glycine,serine and threonine metabolism
Dihydrosphingosine-1P
Phospho-ethanolamine
4.1.2.27
4.1.2.27
Sphingosine-1P
Psychosine
3.1.3.4
3.1.3.-
2.7.1.91
2.4.1.23
Sphingosine
3.1.3.4
3.1.3.-
2.7.1.91
LAC1
LAG1
3.5.1.23
YPC1
Phyto-ceramide
Phyto-sphingosine
L-Serine
3-Dehydro-sphinganine
Dihydro-sphingosine (Sphinganine)
1.14.18.5
1.14.18.5
3.5.1.23
2.3.1.24
2.7.8.10
Ceramide phospho-ethanolamine
2.7.8.-
2.7.8.-
Ceramide ciliatine
Ceramide (N-Acylsphingosine)
2.7.8.27
2.7.8.3
3.1.4.12
2.3.1.24
Sphingomyelin
Sphingosyl-phosphocholine
Palmitoyl-CoA
2.3.1.50
1.1.1.102
2.3.1.24
3.5.1.23
YDC1
DEGS
Dihydro-ceramide
2.4.1.80
2.7.1.138
3.1.4.41
3.1.3.4
3.1.3.-
Ceramide-P
Lactosylceramide
2.4.1.274
3.2.1.23
3.2.1.45
Glucosyl-ceramide
3.2.1.46
CGT
Galactosyl-ceramide
GM4
3.2.1.18
2.8.2.11
Sulfatide
3.1.6.1
3.1.6.8
3.2.1.22
2.4.1.-
Glycosphingolipid biosynthesis - lacto and neolacto series
Glycosphingolipid biosynthesis - globo series
Glycosphingolipid biosynthesis - ganglio series
Cerebroside-sulfatid metabolism
Digalactosylceramide
2.8.2.11
Digalactosylceramide sulfate
Data on KEGG graph
Rendered by Pathview

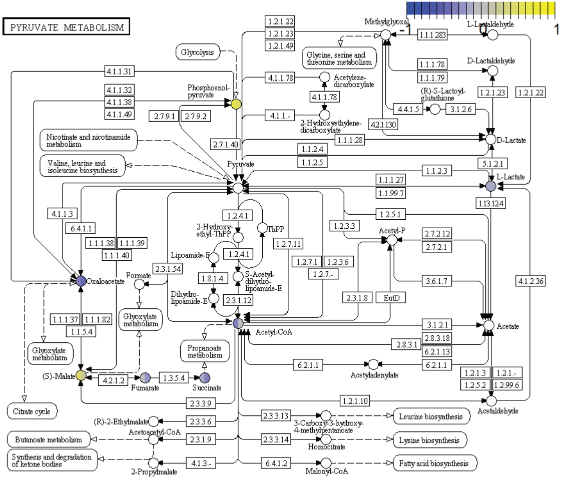

Data on KEGG graph
Rendered by Pathview

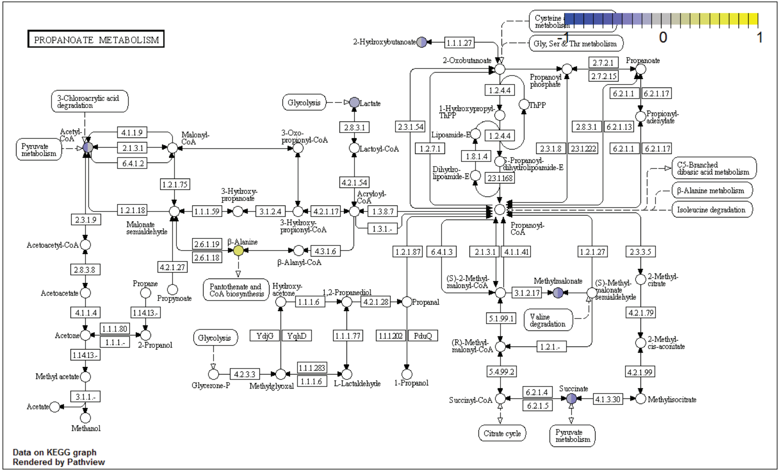

PROPANOATE METABOLISM
-1
0
1
Cysteine metabolism
Gly, Ser & Thr metabolism
2-Hydroxybutanoate
1.1.1.27
2-Oxobutanoate
2.7.2.1
2.7.2.15
Propanoate
Propanoyl phosphate
1.2.4.4
ThPP
1-Hydroxypropyl-ThPP
Lipoamide-E
1.2.4.4
1.8.1.4
S-Propanoyl-dihydrolipoamide-E
Dihydro-lipoamide-E
2.3.1.168
6.2.1.1
6.2.1.17
Propionyl-adenylate
2.8.3.1
6.2.1.13
2.3.1.8
2.3.1.222
6.2.1.1
6.2.1.17
2.3.1.54
1.2.7.1
C5-Branched dibasic acid metabolism
β-Alanine metabolism
Isoleucine degradation
Propanoyl-CoA
3-Chloroacrylic acid degradation
Pyruvate metabolism
Acetyl-CoA
4.1.1.9
2.1.3.1
6.4.1.2
Malonyl-CoA
Glycolysis
Lactate
2.8.3.1
Lactoyl-CoA
3-Oxo-propionyl-CoA
1.2.1.75
1.2.1.18
3-Hydroxy-propanoate
1.1.1.59
3.1.2.4
4.2.1.17
4.2.1.54
Acryloyl-CoA
1.3.8.7
1.3.1.-
Malonate semialdehyde
3-Hydroxy-propionyl-CoA
2.3.1.9
Acetoacetyl-CoA
2.6.1.19
2.6.1.18
β-Alanine
4.3.1.6
β-Alanyl-CoA
4.2.1.27
2.8.3.8
Acetoacetate
Propane
Propynoate
Pantothenate and CoA biosynthesis
Hydroxy-acetone
1.1.1.6
1,2-Propanediol
4.2.1.28
Propanal
1.2.1.87
6.4.1.3
2.1.3.1
4.1.1.41
1.2.1.27
2.3.3.5
4.1.1.4
1.14.13.-
Acetone
1.1.1.80
1.1.1.-
2-Propanol
1.14.13.-
Methyl acetate
3.1.1.-
Acetate
Methanol
Glycolysis
Glycerone-P
4.2.3.3
Methylglyoxal
YdjG
YqhD
1.1.1.283
1.1.1.6
1.1.1.77
L-Lactaldehyde
1.1.1.202
PduQ
1-Propanol
(S)-2-Methyl-malonyl-CoA
3.1.2.17
Methylmalonate
(S)-Methyl-malonate semialdehyde
2-Methyl-citrate
Valine degradation
5.1.99.1
4.2.1.79
(R)-Methyl-malonyl-CoA
1.2.1.-
2-Methyl-cis-aconitate
5.4.99.2
4.2.1.99
Succinyl-CoA
6.2.1.4
6.2.1.5
Succinate
4.1.3.30
Methylisocitrate
Citrate cycle
Pyruvate metabolism
Data on KEGG graph
Rendered by Pathview

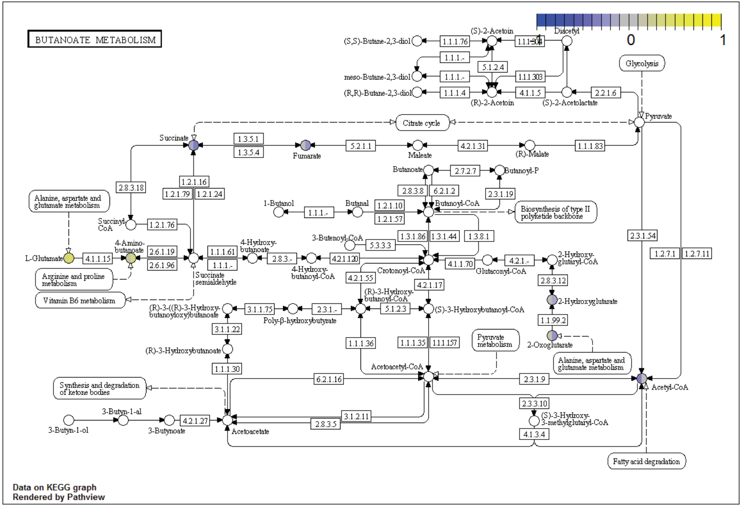

BUTANOATE METABOLISM
-1
0
1
(S,S)-Butane-2,3-diol
meso-Butane-2,3-diol
(R,R)-Butane-2,3-diol
(S)-2-Acetoin
(R)-2-Acetoin
Diacetyl
(S)-2-Acetolactate
1.1.1.76
1.1.1.-
1.1.1.-
1.1.1.4
5.1.2.4
1.1.1.303
4.1.1.5
2.2.1.6
Glycolysis
Pyruvate
Citrate cycle
Succinate
Fumarate
Maleate
(R)-Malate
1.3.5.1
1.3.5.4
5.2.1.1
4.2.1.31
1.1.1.83
Butanoate
Butanoyl-P
2.7.2.7
2.8.3.8
6.2.1.2
2.3.1.19
Butanoyl-CoA
Biosynthesis of type II polyketide backbone
1-Butanol
Butanal
1.1.1.-
1.2.1.10
1.2.1.57
2.8.3.18
1.2.1.16
1.2.1.79
1.2.1.24
Alanine, aspartate and glutamate metabolism
Succinyl-CoA
1.2.1.76
L-Glutamate
4.1.1.15
4-Amino-butanoate
2.6.1.19
2.6.1.96
Succinate semialdehyde
1.1.1.61
1.1.1.-
4-Hydroxy-butanoate
2.8.3.-
4-Hydroxy-butanoyl-CoA
4.2.1.120
3-Butenoyl-CoA
5.3.3.3
1.3.1.86
1.3.1.44
1.3.8.1
Crotonoyl-CoA
4.1.1.70
Glutaconyl-CoA
4.2.1.-
2-Hydroxy-glutaryl-CoA
2.8.3.12
2-Hydroxyglutarate
1.1.99.2
2-Oxoglutarate
Alanine, aspartate and glutamate metabolism
Arginine and proline metabolism
Vitamin B6 metabolism
4.2.1.55
4.2.1.17
(R)-3-Hydroxy-butanoyl-CoA
5.1.2.3
(S)-3-Hydroxybutanoyl-CoA
(R)-3-((R)-3-Hydroxy-butanoyloxy)butanoate
3.1.1.75
2.3.1.-
Poly-β-hydroxybutyrate
3.1.1.22
(R)-3-Hydroxybutanoate
1.1.1.30
1.1.1.36
1.1.1.35
1.1.1.157
Pyruvate metabolism
2.3.1.54
1.2.7.1
1.2.7.11
Acetoacetyl-CoA
2.3.1.9
Acetyl-CoA
6.2.1.16
Synthesis and degradation of ketone bodies
2.3.3.10
(S)-3-Hydroxy-3-methylglutaryl-CoA
4.1.3.4
3.1.2.11
2.8.3.5
3-Butyn-1-ol
3-Butyn-1-al
3-Butynoate
4.2.1.27
Acetoacetate
Fatty acid degradation
Data on KEGG graph
Rendered by Pathview

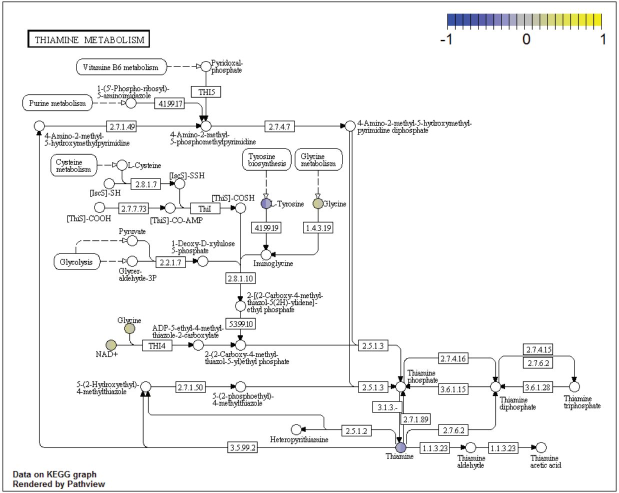
THIAMINE METABOLISM
-1
0
1
Vitamine B6 metabolism
Pyridoxal-phosphate
THI5
Purine metabolism
1-(5'-Phospho-ribosyl)-5-aminoimidazole
4.1.99.17
2.7.1.49
2.7.4.7
4-Amino-2-methyl-5-hydroxymethylpyrimidine diphosphate
4-Amino-2-methyl-5-hydroxymethylpyrimidine
4-Amino-2-methyl-5-phosphomethylpyrimidine
Cysteine metabolism
L-Cysteine
Tyrosine biosynthesis
Glycine metabolism
[IscS]-SSH
[IscS]-SH
2.8.1.7
[ThiS]-COSH
[ThiS]-COOH
2.7.7.73
ThiI
[ThiS]-CO-AMP
L-Tyrosine
Glycine
4.1.99.19
1.4.3.19
Pyruvate
1-Deoxy-D-xylulose 5-phosphate
Glycolysis
2.2.1.7
Glyceraldehyde-3P
Iminoglycine
2.8.1.10
2-[(2-Carboxy-4-methylthiazol-5(2H)-ylidene]-ethyl phosphate
5.3.99.10
Glycine
ADP-5-ethyl-4-methylthiazole-2-carboxylate
NAD+
THI4
2-(2-Carboxy-4-methylthiazol-5-yl)ethyl phosphate
2.5.1.3
2.7.4.16
2.7.4.15
2.7.6.2
Thiamine phosphate
5-(2-Hydroxyethyl)-4-methylthiazole
2.7.1.50
5-(2-phosphoethyl)-4-methylthiazole
2.5.1.3
3.6.1.15
3.6.1.28
Thiamine diphosphate
Thiamine triphosphate
3.1.3.-
2.7.1.89
2.5.1.2
2.7.6.2
Heteropyrithiamine
3.5.99.2
Thiamine
1.1.3.23
Thiamine aldehyde
1.1.3.23
Thiamine acetic acid
Data on KEGG graph
Rendered by Pathview

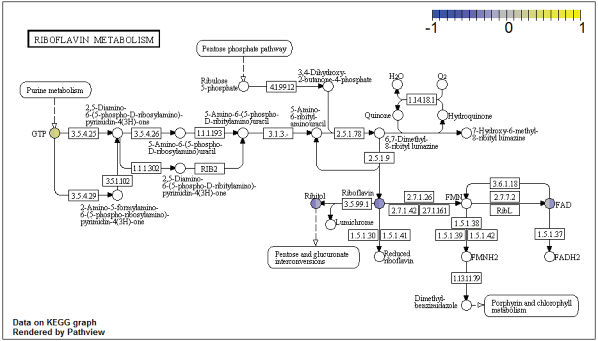

-1
0
1
RIBOFLAVIN METABOLISM
Pentose phosphate pathway
Purine metabolism
Ribulose 5-phosphate
4.1.99.12
3,4-Dihydroxy-2-butanone-4-phosphate
H2O
O2
1.14.18.1
Quinone
Hydroquinone
GTP
3.5.4.25
2,5-Diamino-6-(5-phospho-D-ribosylamino)-pyrimidin-4(3H)-one
3.5.4.26
1.1.1.193
5-Amino-6-(5-phospho-D-ribitylamino)uracil
3.1.3.-
5-Amino-6-ribityl-aminouracil
2.5.1.78
6,7-Dimethyl-8-ribityl lumazine
7-Hydroxy-6-methyl-8-ribityl lumazine
5-Amino-6-(5-phospho-D-ribosylamino)uracil
1.1.1.302
RIB2
2,5-Diamino-6-(5-phospho-D-ribitylamino)-pyrimidin-4(3H)-one
3.5.1.102
3.5.4.29
2-Amino-5-formylamino-6-(5-phospho-ribosylamino)-pyrimidin-4(3H)-one
2.5.1.9
Ribitol
Riboflavin
3.5.99.1
Lumichrome
2.7.1.26
2.7.1.42
2.7.1.161
FMN
3.6.1.18
2.7.7.2
RibL
FAD
1.5.1.38
1.5.1.39
1.5.1.42
1.5.1.30
1.5.1.41
1.5.1.37
Reduced riboflavin
FMNH2
FADH2
Pentose and glucuronate interconversions
1.13.11.79
Dimethyl-benzimidazole
Porphyrin and chlorophyll metabolism
Data on KEGG graph
Rendered by Pathview

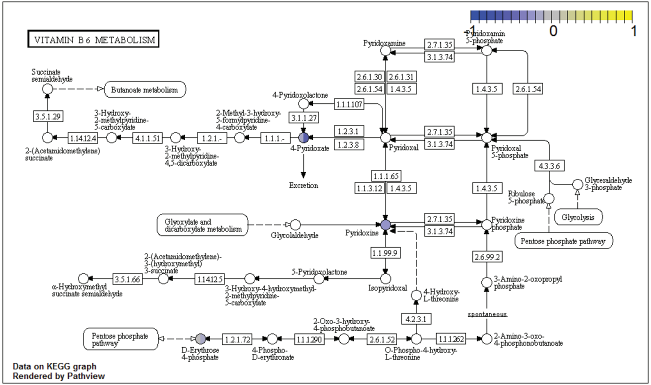

VITAMIN B6 METABOLISM
-1
0
1
Succinate semialdehyde
Butanoate metabolism
3.5.1.29
2-(Acetamidomethylene) succinate
1.14.12.4
3-Hydroxy-2-methylpyridine-5-carboxylate
4.1.1.51
3-Hydroxy-2-methylpyridine-4,5-dicarboxylate
1.2.1.-
2-Methyl-3-hydroxy-5-formylpyridine-4-carboxylate
1.1.1.-
4-Pyridoxolactone
1.1.1.107
3.1.1.27
4-Pyridoxate
Excretion
1.2.3.1
1.2.3.8
Pyridoxamine
2.7.1.35
3.1.3.74
Pyridoxamine 5-phosphate
2.6.1.30
2.6.1.31
2.6.1.54
1.4.3.5
1.4.3.5
2.6.1.54
Pyridoxal
2.7.1.35
3.1.3.74
Pyridoxal 5-phosphate
4.3.3.6
Glyceraldehyde 3-phosphate
Ribulose 5-phosphate
Glycolysis
Pentose phosphate pathway
1.1.1.65
1.1.3.12
1.4.3.5
1.4.3.5
Glyoxylate and dicarboxylate metabolism
Glycolaldehyde
Pyridoxine
2.7.1.35
3.1.3.74
Pyridoxine phosphate
1.1.99.9
2.6.99.2
2-(Acetamidomethylene)-3-(hydroxymethyl)3-succinate
α-Hydroxymethyl succinate semialdehyde
3.5.1.66
1.14.12.5
3-Hydroxy-4-hydroxymethyl-2-methylpyridine-5-carboxylate
5-Pyridoxolactone
Isopyridoxal
4-Hydroxy-L-threonine
3-Amino-2-oxopropyl phosphate
spontaneous
4.2.3.1
2-Oxo-3-hydroxy-4-phosphobutanoate
Pentose phosphate pathway
D-Erythrose 4-phosphate
1.2.1.72
4-Phospho-D-erythronate
1.1.1.290
2.6.1.52
O-Phospho-4-hydroxy-L-threonine
1.1.1.262
2-Amino-3-oxo-4-phosphonobutanoate
Data on KEGG graph
Rendered by Pathview

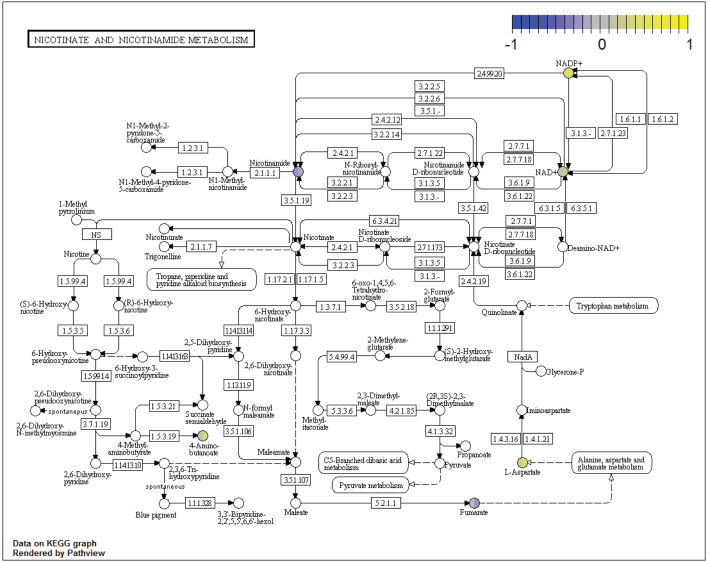
NICOTINATE AND NICOTINAMIDE METABOLISM
-1
0
1
NADP+
2.4.99.20
3.2.2.5
3.2.2.6
3.5.1.-
2.4.2.12
3.2.2.14
1.6.1.1
1.6.1.2
3.1.3.-
2.7.1.23
2.7.7.1
2.7.7.18
N1-Methyl-2-pyridone-5-carboxamide
1.2.3.1
1.2.3.1
N1-Methyl-4-pyridone-5-carboxamide
N1-Methyl-nicotinamide
2.1.1.1
Nicotinamide
2.4.2.1
N-Ribosyl-nicotinamide
3.2.2.1
3.2.2.3
2.7.1.22
Nicotinamide D-ribonucleotide
3.1.3.5
3.1.3.-
NAD+
3.6.1.9
3.6.1.22
3.5.1.19
3.5.1.42
6.3.1.5
6.3.5.1
1-Methyl pyrrolinium
NS
Nicotinurate
2.1.1.7
Trigonelline
Nicotinate
6.3.4.21
Nicotinate D-ribonucleoside
2.4.2.1
2.7.1.173
3.2.2.3
3.1.3.5
3.1.3.-
Nicotinate D-ribonucleotide
Deamino-NAD+
Nicotine
1.5.99.4
1.5.99.4
Tropane, piperidine and pyridine alkaloid biosynthesis
1.17.2.1
1.17.1.5
2.4.2.19
(S)-6-Hydroxy-nicotine
(R)-6-Hydroxy-nicotine
6-Hydroxy-nicotinate
1.3.7.1
6-oxo-1,4,5,6-Tetrahydro-nicotinate
3.5.2.18
2-Formyl-glutarate
Quinolinate
Tryptophan metabolism
1.5.3.5
1.5.3.6
1.14.13.114
1.17.3.3
1.1.1.291
2,5-Dihydroxy-pyridine
6-Hydroxy-pseudooxynicotine
1.14.13.163
6-Hydroxy-3-succinoylpyridine
2-Methylene-glutarate
5.4.99.4
(S)-2-Hydroxy-methylglutarate
NadA
Glycerone-P
1.5.99.14
2,6-Dihydroxy-nicotinate
1.13.11.9
2,6-Dihydroxy-pseudooxynicotine
spontaneous
1.5.3.21
Succinate semialdehyde
N-formyl maleamate
2,3-Dimethyl-maleate
(2R,3S)-2,3-Dimethylmalate
5.3.3.6
4.2.1.85
Iminoaspartate
Methyl-itaconate
2,6-Dihydroxy-N-methylmyosmine
3.7.1.19
4-Methyl-aminobutyrate
1.5.3.19
4-Amino-butanoate
3.5.1.106
4.1.3.32
Propanoate
1.4.3.16
1.4.1.21
Maleamate
C5-Branched dibasic acid metabolism
Pyruvate
L-Aspartate
Alanine, aspartate and glutamate metabolism
2,6-Dihydroxy-pyridine
1.14.13.10
2,3,6-Tri-hydroxypyridine
3.5.1.107
Pyruvate metabolism
spontaneous
Blue pigment
1.1.13.28
3,3'-Bipyridine-2,2',5,5',6,6'-hexol
Maleate
5.2.1.1
Fumarate
Data on KEGG graph
Rendered by Pathview

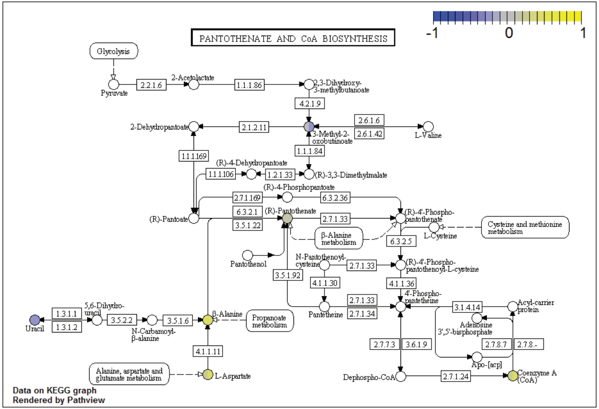

PANTOTHENATE AND CoA BIOSYNTHESIS
-1
0
1
Glycolysis
Pyruvate
2.2.1.6
2-Acetolactate
1.1.1.86
2,3-Dihydroxy-3-methylbutanoate
4.2.1.9
2-Dehydropantoate
2.1.2.11
3-Methyl-2-oxobutanoate
2.6.1.6
2.6.1.42
L-Valine
1.1.1.169
1.1.1.84
(R)-4-Dehydropantoate
1.1.1.106
1.2.1.33
(R)-3,3-Dimethylmalate
(R)-4-Phosphopantoate
2.7.1.169
6.3.2.36
6.3.2.1
(R)-Pantothenate
2.7.1.33
(R)-4'-Phosphopantothenate
(R)-Pantoate
3.5.1.22
β-Alanine metabolism
Cysteine and methionine metabolism
L-Cysteine
6.3.2.5
Pantothenol
N-Pantothenoyl-cysteine
2.7.1.33
(R)-4'-Phospho-pantothenoyl-L-cysteine
3.5.1.92
4.1.1.30
4.1.1.36
4'-Phospho-pantetheine
2.7.1.33
2.7.1.34
Pantetheine
3.1.4.14
Acyl-carrier protein
Adenosine 3',5'-bisphosphate
Uracil
1.3.1.1
1.3.1.2
5,6-Dihydro-uracil
3.5.2.2
N-Carbamoyl-β-alanine
3.5.1.6
β-Alanine
Propanoate metabolism
4.1.1.11
Alanine, aspartate and glutamate metabolism
L-Aspartate
2.7.7.3
3.6.1.9
2.7.8.7
2.7.8.-
Apo-[acp]
Dephospho-CoA
2.7.1.24
Coenzyme A (CoA)
Data on KEGG graph
Rendered by Pathview

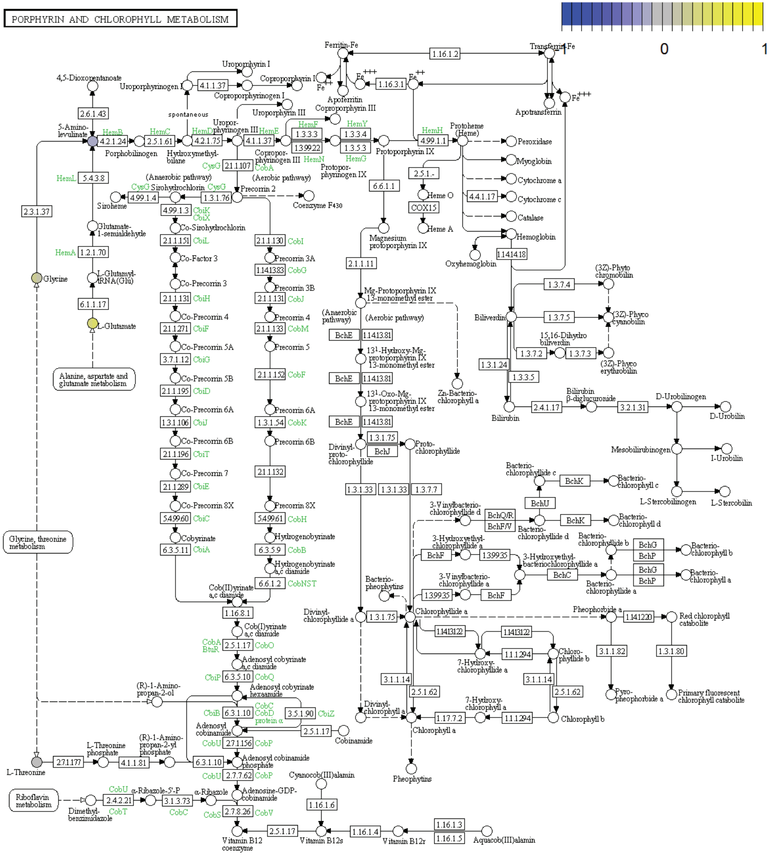

Data on KEGG graph
Rendered by Pathview

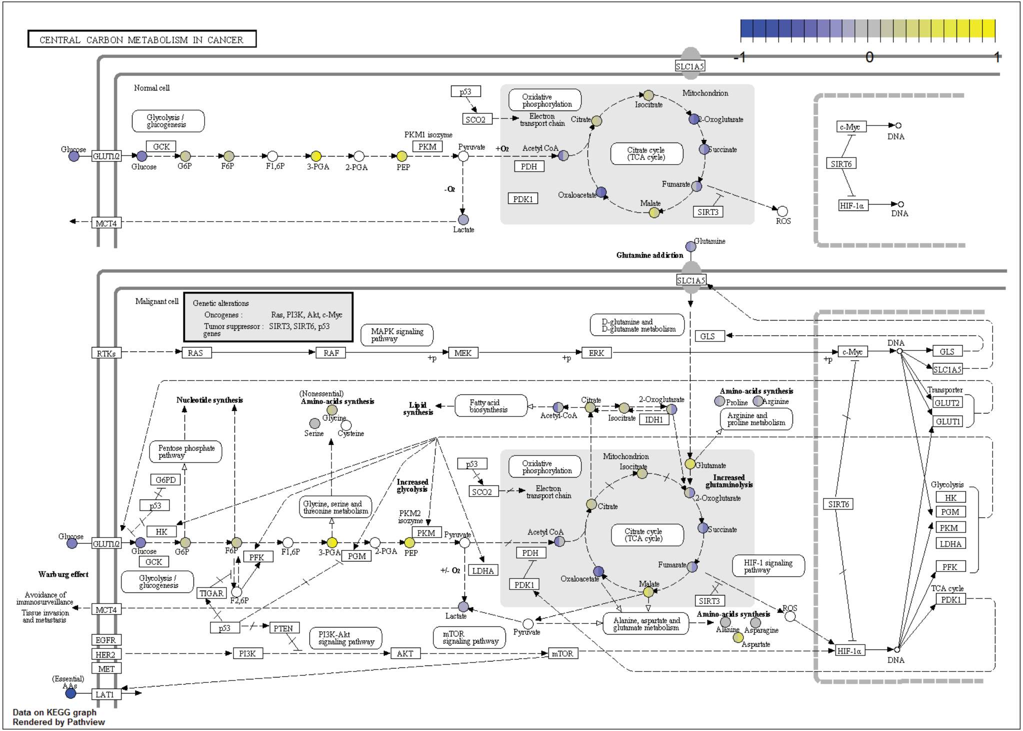
CENTRAL CARBON METABOLISM IN CANCER
-1
0
1
SLC1A5
Normal cell
Glycolysis / glucogenesis
Glucose
GLUT1/2
GCK
Glucose
G6P
F6P
F1,6P
3-PGA
2-PGA
PEP
PKM1 isozyme
PKM
Pyruvate
+O2
-O2
Lactate
MCT4
p53
SCO2
Electron transport chain
Oxidative phosphorylation
Mitochondrion
Acetyl CoA
PDH
PDK1
Citrate
Isocitrate
2-Oxoglutarate
Succinate
Fumarate
Malate
Oxaloacetate
Citrate cycle (TCA cycle)
SIRT3
ROS
c-Myc
DNA
SIRT6
HIF-1α
Glutamine
Glutamine addiction
Malignant cell
Genetic alterations
Oncogenes : Ras, PI3K, Akt, c-Myc
Tumor suppressor genes : SIRT3, SIRT6, p53
MAPK signaling pathway
D-glutamine and D-glutamate metabolism
RTKs
RAS
RAF
MEK
ERK
+p
GLS
GLUT2
GLUT1
Transporter
Nucleotide synthesis
(Nonessential) Amino-acids synthesis
Glycine
Serine
Cysteine
Lipid synthesis
Fatty acid biosynthesis
Acetyl-CoA
IDH1
Amino-acids synthesis
Proline
Arginine
Arginine and proline metabolism
Pentose phosphate pathway
G6PD
HK
Glycine, serine and threonine metabolism
Increased glycolysis
PKM2 isozyme
Increased glutaminolysis
Glutamate
Glycolysis
PGM
PKM
LDHA
PFK
TCA cycle
Warburg effect
Avoidance of immnosurveillance
Tissue invasion and metastasis
PFK
TIGAR
F2,6P
+/- O2
LDHA
HIF-1 signaling pathway
Alanine, aspartate and glutamate metabolism
Alanine
Asparagine
Aspartate
PTEN
PI3K-Akt signaling pathway
mTOR signaling pathway
EGFR
HER2
MET
PI3K
AKT
mTOR
(Essential) AAs
LAT1
Data on KEGG graph
Rendered by Pathview
